# Optimizing twin prime editing components for scalable genome editing and therapy in spinocerebellar ataxia type 3

**DOI:** 10.1016/j.omtn.2026.102988

**Published:** 2026-06-17

**Authors:** Lee Wha Gwon, Jung Bae Seong, Hyeon-Gu Yeo, Yeounsun Oh, Junghyung Park, Jinyoung Won, Sang Je Park, Young-Hyun Kim, Jae-won Huh, Aryun Kim, Kwang-Hyun Park, Youngjeon Lee, Seung Hwan Lee

**Affiliations:** 1 Department of Life Science, Chung-Ang University, Seoul 06974, Republic of Korea; 2 National Primate Research Center (NPRC), Korea Research Institute of Bioscience and Biotechnology (KRIBB), Cheongju 28116, Republic of Korea; 3 KRIBB School of Bioscience, University of Science and Technology (UST), Daejeon 34113, Republic of Korea; 4 Department of Functional Genomics, KRIBB School of Bioscience, University of Science and Technology (UST), Daejeon 34113, Republic of Korea; 5 Department of Neurology, College of Medicine, Chungbuk National University, Cheongju, Republic of Korea; 6 Department of Neurology, Chungbuk National University Hospital, Cheongju, Republic of Korea; 7 KRIBB School of Bioscience, Korea University of Science and Technology, Daejeon 34113, Republic of Korea; 8 Critical Diseases Diagnostics Convergence Research Center, Korea Research Institute of Bioscience and Biotechnology (KRIBB), Daejeon 34141, Republic of Korea

**Keywords:** MT: RNA/DNA Editing, highly efficient, precise, twin prime editing, scalable, gene editing, SCA3, therapy

## Abstract

Recent advances in prime editing technologies using CRISPR modules fused with reverse transcriptase (RT) have enabled efficient and precise reprogramming of target genomic sequences. Twin prime editing using two coordinated prime editor complexes is a promising strategy for inducing extensive genomic modifications via reverse-transcribed complementary templates. However, current twin prime editing systems still require improvements in editing efficiency, accuracy, and intended edit predictability. Here, efficiency and precision of twin prime editing were enhanced via engineering and optimizing conventional SpCas9(H840A)-RT-based prime editor (twin-PE) components. A La-domain-fused prime editor (La-twin-PE) and optimized prime editing guide RNAs (pegRNAs) were developed, achieving a 1.75 ± 0.21-fold increase in gene editing efficiency at multiple genomic loci in human-derived cell lines without increasing unintended indel or inaccurate editing. La-twin-PE facilitated efficient ∼2.8 kb GFP transgene knockin at target loci and eliminated the expanded polyQ tract in *ATXN3* in an engineered human-derived mutant cell line modeling spinocerebellar ataxia type 3. The optimized twin prime editing platform facilitates highly efficient and scalable genomic engineering through streamlined pegRNA design, offering substantial potential for a broad spectrum of biotechnological and therapeutic applications.

## Graphical abstract

**Figure:**
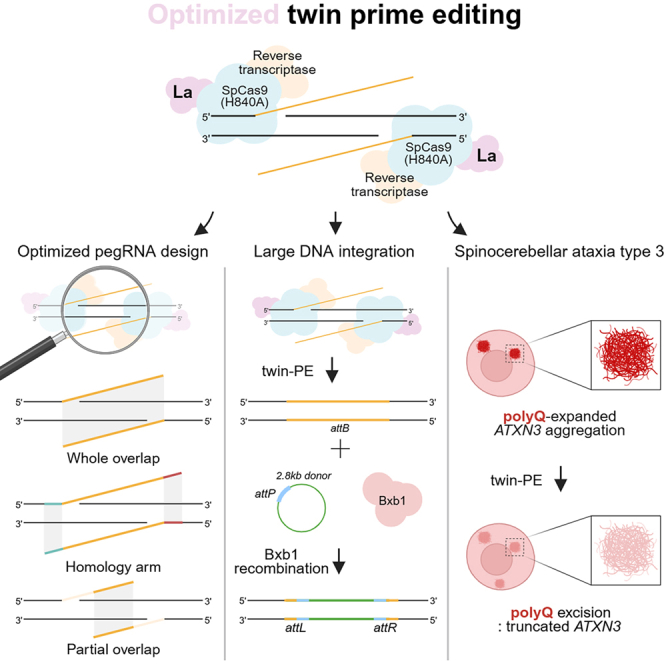

## Teaser

Gwon and colleagues enhance twin prime editing efficiency by combining La-domain fusion with optimized paired pegRNA designs. This strategy improves precise sequence installation, enables Bxb1-mediated donor DNA insertion, and provides proof-of-concept editing in human-derived mutant HEK293FT-*ATXN3*-Q84 cells.

## Introduction

CRISPR technology enables targeted DNA editing based on reprogrammed guide RNAs.^1^^,^^2^ It has rapidly evolved into advanced forms enabling high target-specific gene editing, from prime editing (PE)-based universal single-base gene editing^3^^,^^4^^,^^5^^,^^6^ to large-scale DNA editing.^7^^,^^8^^,^^9^ PE technology fuses a reverse transcriptase (RT) to a CRISPR-Cas9 module targeting specific DNA to induce versatile gene editing using a target DNA-matching prime editing guide RNA (pegRNA).^4^^,^^10^ Recently, engineered versions of the prime editing module, PE2 or PE3, incorporate function-specific domains to improve gene editing accuracy and efficiency.^11^^,^^12^^,^^13^^,^^14^ A major limitation of this prime editing technology lies in difficulty of integrating long edited DNA flaps into target sites after RT copies pegRNA, making it challenging to edit regions longer than a few base pairs.^5^ This twin prime editing (twin-PE) approach uses two prime editor modules to create extended complementary DNA flaps for inducing deletion, replacement, or insertion, enabling efficient large-scale DNA editing that a single prime editor cannot handle.^15^^,^^16^^,^^17^^,^^18^^,^^19^^,^^20^ Twin-PE currently shows minimal off-target editing and is an effective gene editing strategy, particularly when combined with other recombinases.^7^^,^^8^^,^^9^ This study aimed to improve twin-PE efficiency and precision by optimizing pegRNAs, components of the prime editor, and attaching the functional La protein domain to enable high-efficiency large-scale editing. In this study, we demonstrate that the twin-PE strategy, utilizing fully matched pegRNAs developed herein and the La-SpCas9(H840A)-RT (La-twin-PE) module, achieves superior genome editing efficiency and precision compared to conventional systems employing SpCas9(H840A)-RT (twin-PE). Across multiple genomic loci in human-derived cell lines, this optimized twin-PE system exhibited significantly enhanced editing efficiency while minimizing unintended insertions and deletions (indels), thereby improving editing fidelity. Notably, sequential application of twin-PE and the Bxb1 recombinase enabled precise insertion of large gene cassettes. These findings collectively establish a robust and accurate genome engineering framework capable of inducing large-scale genetic modifications in human cells, thereby advancing the translational potential of this platform for therapeutic applications *in vivo*. The proof-of-concept study in the SCA3 model suggests this platform could make a substantial contribution to gene therapy, particularly in the correction of complex and extensive genetic mutations, such as those found in hemophilia^21^^,^^22^^,^^23^ and Huntington disease,^24^^,^^25^ that are challenging to address using conventional genome editing technologies.

## Results

### Improvement of twin prime editing efficiency in human-derived cell lines via optimization of prime editor components and targeting strategies

**Figure 1:**
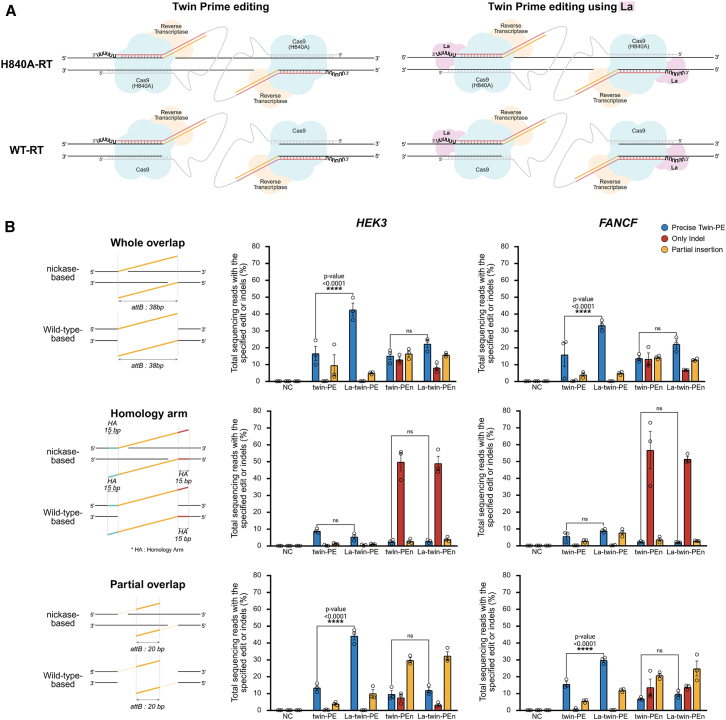
Optimization of prime editor components and gene targeting strategies for effective twin prime editing in human-derived cells (A) Schematic of twin prime editing using conventional prime editors based on nickase (H840A) or wild-type (WT) SpCas9 modules or prime editors engineered with the La domain. RT: reverse transcriptase, La: La domain (1–194 aa), SpCas9(H840A): SpCas9 nickase, SpCas9(WT): SpCas9 wild-type. (B) Comparative experiment assessing the efficiency of 38 bp *attB* sequence insertion using twin prime editing at target gene loci (*HEK3* and *FANCF*) in human-derived cells (HEK293FT). The prime editor was combined with three types of pegRNA overlap strategies based on whether SpCas9 was nickase (H840A) or wild-type (WT) and whether the La domain (1–194 aa) was fused to SpCas9. SpCas9(H840A/WT)-RT: prime editor based on the nickase (H840A) or wild-type (WT) SpCas9 module; La-SpCas9(H840A/WT)-RT: prime editor based on the nickase (H840A) or wild-type (WT) SpCas9 module fused with La domain; whole overlap pegRNA pair: the entire sequence to be inserted into target DNA is fully complementary to both pegRNAs; homology arm pegRNA pair: both pegRNAs include the sequence to be inserted alongside homology arm regions corresponding to target DNA; partial overlap pair: only 20 bp of the sequence to be inserted into target DNA is complementarily shared between the two pegRNAs. Twin prime editing efficiency and pattern analysis were classified into three forms: precise twin-PE (%), only indel (%), and partial insertion (%). The frequency (%) of each was measured (Figure S3). Each histogram represents the mean ± standard error of the mean (SEM) from three independent measurements. *p* values were calculated using two-way ANOVA and Dunnett’s test (ns: not significant; ∗*p* = 0.0332, ∗∗*p* = 0.0021, ∗∗∗*p* = 0.0002, ∗∗∗∗*p* < 0.0001). Precise twin-PE (%): the proportion of precisely edited alleles reflecting the intended twin prime editing outcome. Only indel (%): the frequency of unintended editing events resulting solely in insertions or deletions without intended sequence insertion. Partial insertion (%): the rate of imprecise editing events characterized by incomplete incorporation of the target insertion sequence. NC, negative control; twin-PE: SpCas9(H840A)-RT-based twin-PE; La-twin-PE: La-SpCas9(H840A)-RT-based twin-PE; twin-PEn: SpCas9(WT)-RT-based twin-PE; La-twin-PEn: La-SpCas9(WT)-RT-based twin-PE.

In this study, various strategic approaches were explored to improve twin-PE efficiency and precision (Figure 1). First, protein-level engineering was introduced to enhance the efficiency of each prime editor (Figure 1A**)**. Conventional prime editors^4^^,^^26^ were constructed by fusing a reverse transcriptase (RT) to SpCas9 in its nickase (H840A) or wild-type form, resulting in twin-PE and SpCas9(WT)-RT (twin-PEn). Here, engineered La-twin-PE and La-SpCas9(WT)-RT (La-twin-PEn) were developed via La domain fusion,^13^ and these four protein module combinations were used to induce twin-PE (Figure S1). Second, pegRNA PBS and reverse transcription template (RTT) region length and complementarity between the target DNA and pegRNA pair were adjusted to induce optimized twin-PE outcomes (Figure 1B**)**. Twin-PE at the three genomic loci (*HEK3, FANCF*, and *AAVS1*) in human-derived cell lines (HEK293FT) (Figure S2) yielded highly consistent editing results. Notably, twin-PE showed higher accuracy in inserting *attB* or *attP* sequences into target DNA compared with twin-PEn (Figures 1B, S3, and S4). Gene editing patterns included complete insertion of *attB* or *attP* sequences (precise twin-PE) and partial insertion of *attB*/*attP* sequences or complete indels (Figure S3). Partial insertion and complete indel rates were higher when twin-PE was induced with twin-PEn than with twin-PE (Figures 1B**,** S3, and S4). When designing pegRNAs for each twin prime editor, three distinct types were employed: fully complementary (whole overlap), homology arm-forming (homology arm), and partially complementary (partial overlap) (Figures 1B and S4, left). Across all target loci, pegRNA designs based on either the “whole overlap” or “partial overlap” strategy consistently yielded higher-fidelity twin-PE than the “homology arm” design, regardless of whether twin-PE or twin-PEn was used (Figures 1B and S4, right). Notably, when twin-PE was induced using the twin-PEn module in combination with pegRNAs, whose complementary binding regions formed homology arms with the target DNA, a substantial portion of the editing outcomes consisted of precisely generated indels (Figures 1B and S4, middle row). Among all twin prime editor combinations, precise editing efficiency significantly increased (1.40 ± 0.26-fold increase) when La-domain-fused La-twin-PE or La-twin-PEn modules were designed with fully or partially complementary pegRNAs (Figure 1B**,** top and bottom row). However, no increase was observed in any of the three genomic sites (*HEK3*, *FANCF*, and *AAVS1*) in designs forming homology arms at the same target loci (Figure 1B**,** middle row). When different sequences (*attB* or *attP*) were inserted into the target gene (*AAVS1*), similar editing trends were observed across all four twin-PE combinations (Figure S4). Additionally, compared with PRIME-Del,^19^ which has a similar design based on homology arms, La-twin-PE significantly increased overall precise *attB* insertion efficiency in both HEK293FT and HeLa cells (2.43 ± 0.62-fold and 5.45 ± 1.40-fold increases, respectively) across five genomic sites (*HEK3*, *VEGFA*, *DAPK1*, *CCR5*, and *PIN1*) (Figure S5). La-twin-PE was selected as the optimized twin-PE module, and all subsequent experiments were conducted using pegRNAs designed in the “whole overlap” configuration, with comparative analyses performed against twin-PE.

### Comparison of the efficiency of the optimized La-domain-conjugated twin-PE and the epegRNA-based twin-PE system

**Figure 2:**
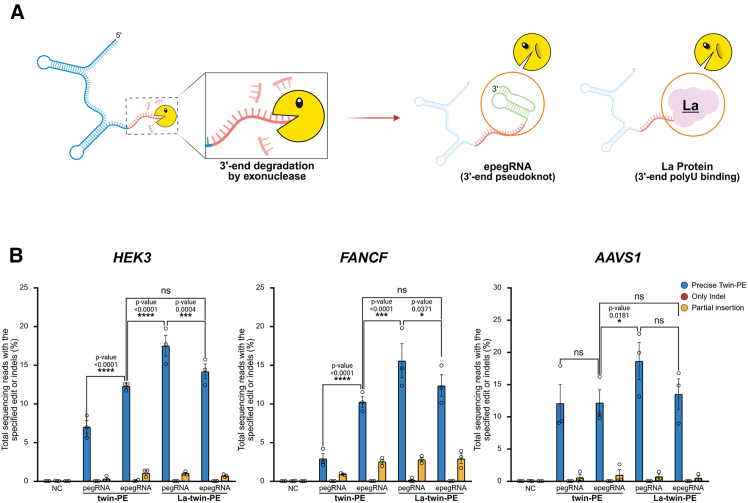
Comparative analysis of epegRNA-guided and La-fused twin prime editing system (A) Schematic illustrating the protection of the pegRNA 3′-end from exonucleolytic degradation by employing either an epegRNA architecture or a prime editor conjugated to the La domain. (B) Comparative experiment of the insertion efficiency of *attB* (*HEK3* and *FANCF*) and *attP* (*AAVS1*) sequences using twin-PE with conventional pegRNAs, epegRNAs, or a combined system integrating the twin prime editor with the La domain (1–194 aa). Each histogram represents the mean ± standard error of the mean (SEM) from three independent measurements. *p* values were calculated using two-way ANOVA and Dunnett’s test (ns: not significant; ∗*p* = 0.0332, ∗∗*p* = 0.0021, ∗∗∗*p* = 0.0002, ∗∗∗∗*p* < 0.0001). Precise twin-PE (%): the proportion of precisely edited alleles reflecting the intended twin prime editing outcome. Only indel (%): the frequency of unintended editing events resulting solely in insertions or deletions without intended sequence insertion. Partial insertion (%): the rate of imprecise editing events characterized by incomplete incorporation of the target insertion sequence. NC, negative control; twin-PE: SpCas9(H840A)-RT-based twin-PE; La-twin-PE: La-SpCas9(H840A)-RT-based twin-PE; pegRNA: conventional pegRNA; epegRNA: 3′-end pseudoknot-conjugated enhanced pegRNA.

Among the foundational efforts to enhance the efficiency of prime editing, the engineered pegRNA (epegRNA)-based system, which incorporates a 3′-end RNA structural stabilization element, has demonstrated markedly improved editing outcomes.^14^ In this study, we further investigated how the optimized La-twin-PE system compares to the previously engineered epegRNA-based twin-PE system with respect to gene editing performance (Figure 2). Across multiple endogenous loci in human-derived HEK293FT cells (*HEK3, FANCF*, and *AAVS1*), we delivered combinations of pegRNA or epegRNA together with either twin-PE or the optimized La-twin-PE module to induce twin-PE (Figure 2A**)**. As expected, twin-PE exhibited enhanced editing efficiencies when used in conjunction with epegRNAs (Figure 2B**)**. In contrast, the simultaneous use of epegRNAs with the La-twin-PE module consistently resulted in suppressed editing efficiency, indicating a distinct and reproducible inhibitory interaction. Notably, for all three endogenous sites tested, the optimized La-twin-PE module combined with conventional pegRNAs yielded substantially higher editing efficiencies (1.49 ± 0.11-fold) compared with those achieved by the previously reported epegRNA-based twin-PE system, underscoring the superior activity and technical robustness of the optimized La-domain-conjugated twin-PE architecture.

### Efficient induction of twin prime editing across multiple genomic loci in human-derived cells

**Figure 3:**
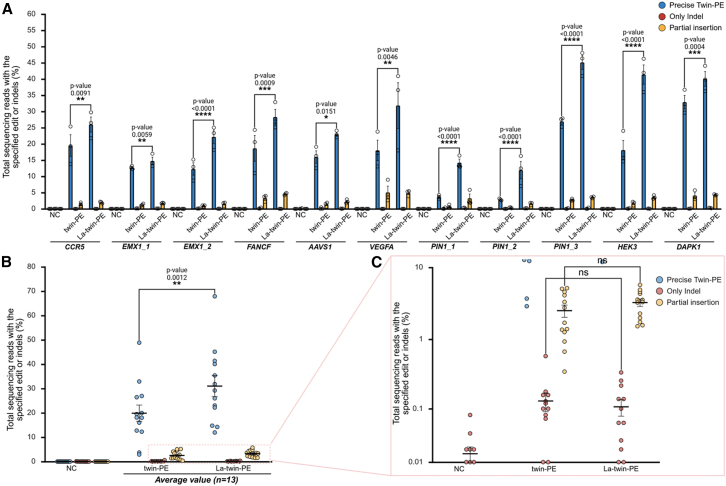
Verification of optimized twin prime editing efficiency targeting various endogenous genes in human-derived cells (A) Comparison of twin prime editing efficiency (%) based on twin-PE or La-twin-PE targeting 11 endogenous loci in human-derived cells (HEK293FT). (B) Histogram showing the average twin prime editing efficiency at 13 endogenous loci (*n* = 13, including the final two loci presented in Figure 5) in human-derived cells. (C) The inset provides enlarged views of the representative “only indel” and “partial insertion” patterns generated by twin prime editing, as identified in panel (B). Each histogram represents the mean ± standard error of the mean (SEM) from three independent experiments. *p* values were calculated using two-way ANOVA and Dunnett’s test (ns: not significant; ∗*p* = 0.0332, ∗∗*p* = 0.0021, ∗∗∗*p* = 0.0002, ∗∗∗∗*p* < 0.0001). NC, negative control; twin-PE: SpCas9(H840A)-RT-based twin-PE; La-twin-PE: La-SpCas9(H840A)-RT-based twin-PE. Precise twin-PE (%): the proportion of precisely edited alleles reflecting the intended twin prime editing outcome. Only indel (%): the frequency of unintended editing events resulting solely in insertions or deletions without intended sequence insertion. Partial insertion (%): the rate of imprecise editing events characterized by incomplete incorporation of the target insertion sequence.

Based on previous experimental results, additional validation experiments were conducted using human-derived cells to induce precise sequence insertion into target DNA via twin-PE (Figure 3). Twin-PE using the nickase (H840A) form shows more precise gene editing than that using the wild-type (WT) form (Figures 1 and S4). Therefore, we compared twin-PE efficiency and precision at 13 genomic loci using the conventional twin-PE and the engineered, optimized La-twin-PE modules (Figure 3A**)**. The results showed that using the La-twin-PE module causes a significant increase (average 1.75 ± 0.21-fold) in precise gene editing efficiency over twin-PE-based twin-PE (Figure 3B**)**. As anticipated, in contrast to twin-PE employing the wild-type module, both the twin-PE and La-twin-PE modules exhibited minimal unintended indel formation. Importantly, a low frequency of partial insertions, presumably resulting from the malfunction of a single prime editor, was detected as a byproduct of the twin-PE process (Figure 3C**)**. Nevertheless, La-twin-PE did not show a statistically significant increase in unintended indel formation (average 0.11% ± 0.03%) relative to twin-PE (average 0.13% ± 0.04%). Taken together, the La-twin-PE module achieved a markedly higher ratio of precise gene insertion to total aberrant outcomes (precise twin-PE/partial insertion + indel only), while maintaining a comparable unintended indel frequency across multiple genomic loci in human-derived cells (Figures 3B and 3C**)**.

### Comparative validation of off-target effects using the optimized twin prime editing system

**Figure 4:**
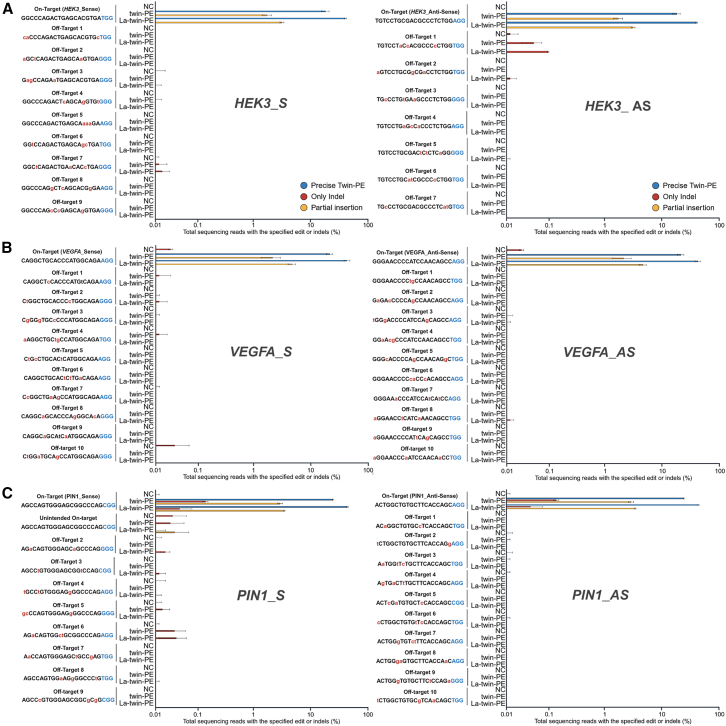
Off-target effect analysis of optimized twin prime editing in human-derived cells Twin prime editing used twin-PE or La-twin-PE at three endogenous gene loci [*HEK3* (A), *VEGFA* (B), *PIN1* (C)] in human-derived cells (HEK293FT). Off-target candidate sequences predicted *in silico* for each pegRNA pair (S-/AS-strand targeting) were analyzed using targeted amplicon sequencing. Sequences listed on the left side of each histogram represent on-target sequences used for twin prime editing and off-target sequences predicted *in silico* based on those on-targets. Blue letters indicate the PAM (NGG) sequence recognized by SpCas9. Red lowercase letters indicate mismatched bases relative to the on-target sequence. S, sense; AS, antisense; NC, negative control; twin-PE: SpCas9(H840A)-RT-based twin-PE; La-twin-PE: La-SpCas9(H840A)-RT-based twin-PE. Precise twin-PE (%): the proportion of precisely edited alleles reflecting the intended twin prime editing outcome. Only indel (%): the frequency of unintended editing events resulting solely in insertions or deletions without intended sequence insertion. Partial insertion (%): the rate of imprecise editing events characterized by incomplete incorporation of the target insertion sequence.

Next, to compare and analyze the accuracy of La-twin-PE-induced twin-PE with those of the conventional twin-PE module, *attB* sequence insertion was examined at various genomic loci (*HEK3*, *VEGFA*, and *PIN1*) (Figure 4). To simultaneously confirm off-target editing at genomic loci sequence with similarity to the targets, targeted amplicon sequencing was conducted on candidate sites obtained via *in silico* prediction,^27^ allowing up to three mismatches, and the top 10 prioritized predicted sites for each pegRNA pair were selected for targeted analysis. For each pegRNA pair used in twin-PE, off-target candidates on both the sense (Figure 4, left) and antisense (Figure 4, right) strands were individually detected with high resolution (indel frequency >0.1%), corresponding to the typical background error rate of amplicon sequencing, and were comparable to those observed in untreated control samples. Subsequently, targeted amplicons were generated for each sample edited with either the optimized La-twin-PE module or the conventional twin-PE module, followed by NGS-based sequencing and comparative analysis. Consequently, except for a minimal signal (indel frequency ∼0.1%) detected at an off-target candidate (off-target 1) corresponding to the *HEK3* antisense pegRNA (Figure 4, top, right), no significant off-target editing was observed for any of the pegRNA pairs at the three tested genomic loci. Additionally, we evaluated a *PIN1* sense pegRNA target sequence present at a different genomic locus (unintended on-target site). Although paired pegRNAs induced approximately 45% *attB* insertion at the intended target site, ∼0.03% *attB* insertion was detected on average at the unintended on-target site (Figure 4, bottom, left). These results suggest that efficient twin-PE requires coordinated paired pegRNA activity and may reduce unintended editing arising from isolated single-pegRNA activity and indicate that using the optimized La-twin-PE module maximizes editing efficiency without increasing off-target effects. Specifically, the twin-PE mechanism based on complementary pegRNA pairing using the La-twin-PE module demonstrated high efficiency and safety, suggesting its potential applicability to human gene therapy.

### Induction of large-scale gene insertion using the optimized twin prime editing system

**Figure 5:**
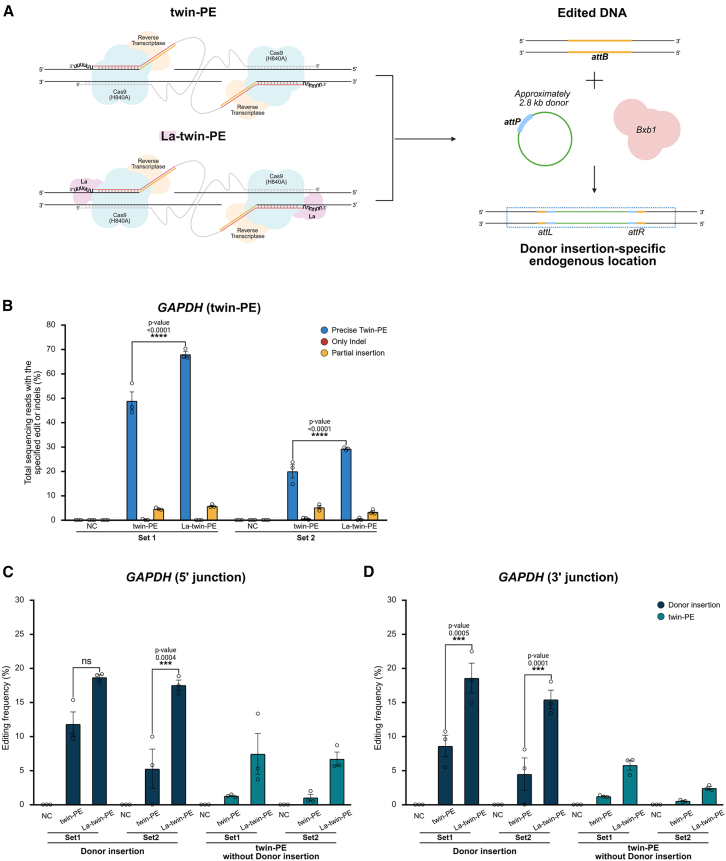
Quantification of Bxb1-mediated donor DNA insertion following optimized twin prime editing in human-derived cells (A) Schematic of large DNA insertion using sequential reactions of twin prime editing and Bxb1 recombinase in human-derived cells (HEK293FT). After inducing *attB* sequence insertion at a genomic locus using twin-PE or La-twin-PE, donor DNA containing an *attP* sequence was recombined via Bxb1 to insert *GFP*. (B) Comparison of twin prime editing efficiency at the *GAPDH* locus in human-derived cells using twin-PE or La-twin-PE. Sets 1 and 2 represent the respective combinations of pegRNAs used for twin prime editing at the *GAPDH* locus. Precise twin-PE (%): the proportion of precisely edited alleles reflecting the intended twin prime editing outcome. Only indel (%): the frequency of unintended editing events resulting solely in insertions or deletions without intended sequence insertion. Partial insertion (%): the rate of imprecise editing events characterized by incomplete incorporation of the target insertion sequence. (C and D) Comparison of donor DNA insertion efficiency following sequential twin prime editing and Bxb1-mediated recombination. NGS-based quantification of donor insertion and remaining non-converted twin-PE products at the 5′ and 3′ junctions of *GAPDH* locus in cells treated with each prime editor and the Bxb1 recombinase. Donor DNA insertion (%) = donor-specific read count/(donor-specific read count + NC read count) × 100. Each histogram represents mean ± SEM from three independent experiments. *p* values were calculated by two-way ANOVA and Dunnett’s test (ns: not significant, ∗*p* = 0.0332, ∗∗*p* = 0.0021, ∗∗∗*p* = 0.0002, ∗∗∗∗*p* < 0.0001). NC, negative control; twin-PE: SpCas9(H840A)-RT-based twin-PE; La-twin-PE: La-SpCas9(H840A)-RT-based twin-PE.

Large-scale genomic regions within target DNA can be edited using twin-PE.^15^^,^^19^ Thus, we investigated whether efficient large-scale genome editing could be induced in human-derived cells using the La-twin-PE module, which has been validated for both efficiency and safety in this study (Figure 5). In recently developed technologies such as PASSIGE^8^ or PASTE,^9^ donor sequence insertion is facilitated via Bxb1 recombinase using *attB* sequences inserted into target genes using single or dual prime editor modules. Consequently, we hypothesized that if high-efficiency *attB* insertion into target DNA is achieved via twin-PE using the La-twin-PE module, the resulting difference in twin-PE efficiency improves donor sequence insertion via Bxb1 within an effective range. To test this hypothesis, GFP insertion efficiency into target DNA using both twin-PE and La-twin-PE modules was compared (Figure 5A**)**. The donor vector was designed to be approximately 2.8 kb and included a recombinase recognition sequence (*attP*) along with the GFP coding sequence. Sequential *attB* and donor vector insertion into the target DNA (*GAPDH* locus) via twin-PE was confirmed feasible (Figure S6A). Therefore, the La-twin-PE module significantly increased precise *attB* insertion efficiency via twin-PE compared to twin-PE, without substantially changing unintended indel formation (Figure 5B**)**. When two pairs of pegRNAs (sets 1 and 2) were used to target the *GAPDH* locus via twin-PE and the *PIN1* locus as previously shown in Figure 4, a similar trend of increased editing efficiency with La-twin-PE was observed. Donor insertion efficiencies at both the 5′ and 3′ junctions were additionally quantified by NGS-based analysis through comparison with control (NC) reads (Figure S6B). Similar donor insertion frequencies were observed between the 5′ and 3′ junctions at both the *GAPDH* (Figures 5C and 5D**)** and *PIN1* loci (Figure S6C), and significant large-scale donor insertion was induced when using La-twin-PE (average 1.60 ± 0.57-fold and 1.48 ± 0.59-fold increase at the 5′ and 3′ junctions, respectively). Therefore, twin-PE using two pegRNA pairs was conducted, followed by Bxb1-mediated donor vector insertion. These results supported the original hypothesis that La-twin-PE is more effective than twin-PE and. Furthermore, *attB* sequence insertion via twin-PE enabled efficient recombination with an *attP*-GFP donor vector.

### Therapeutic application of the optimized twin prime editing system in SCA3

**Figure 6:**
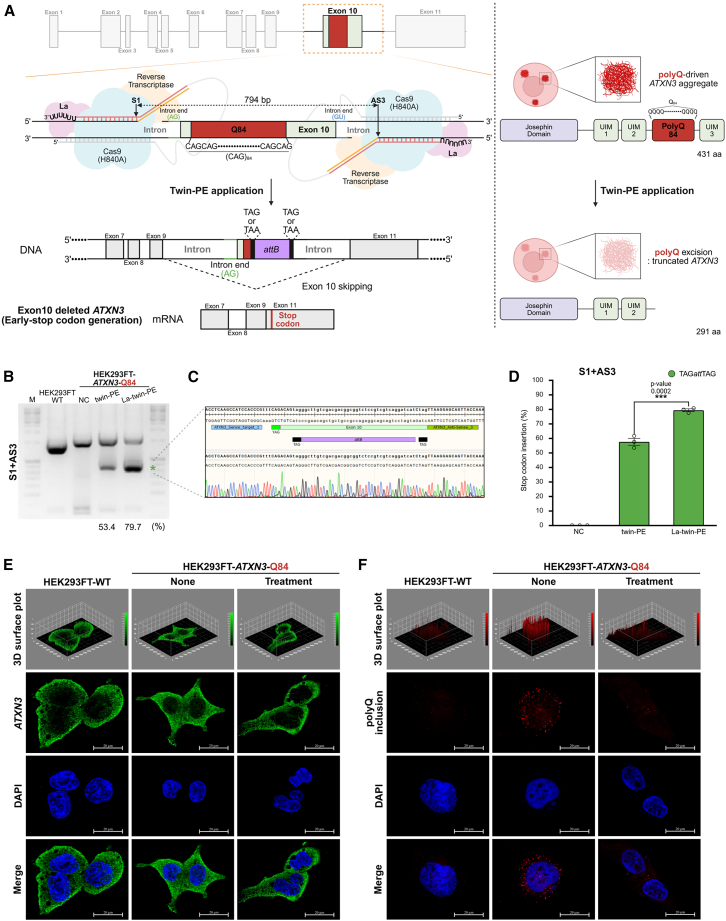
Validation of a therapeutic concept using optimized twin prime editing targeting the polyQ repeat in SCA3 model cell line (A) Schematic illustrating the strategy for insertion of an early stop codon (TAA or TAG) and removal of polyQ repeats in *ATXN3* of a human-derived SCA3 disease model cell line using twin prime editing. The left panel shows that each pegRNA pair targets the upstream region (intron between exon 9 and exon 10) and downstream region (intron between exon 10 and exon 11) of polyQ in exon 10 of *ATXN3*, leading to a stop codon insertion upstream of the polyQ sequence and expression of a truncated *ATXN3* protein. The figure on the right compares aberrant *ATXN3* protein aggregation in the mutant HEK293FT-*ATXN3*-Q84 cell line with the expression of a truncated *ATXN3* protein lacking the polyQ tract, generated via exon 10 skipping induced by twin prime editing. (B) Comparison of twin prime editing efficiency in the *ATXN3* polyQ region of SCA3 model cells using twin-PE or La-twin-PE. Downward shift of the lower band in the gel indicates successful stop codon insertion. (C) Sanger sequencing confirming correct stop codon insertion from the lower-shifted bands in (B). (D) Histogram quantifying twin prime editing efficiency, calculated as stop codon insertion (%) = size-normalized bottom band intensity/size-normalized total band intensity. Each histogram represents the mean ± SEM of three independent experiments. Statistical difference was evaluated using one-way ANOVA and Dunnett’s test (ns: not significant; ∗*p* = 0.0332, ∗∗*p* = 0.0021, ∗∗∗*p* = 0.0002, ∗∗∗∗*p* < 0.0001). NC, negative control; twin-PE: SpCas9(H840A)-RT-based twin-PE; La-twin-PE: La-SpCas9(H840A)-RT-based twin-PE. (E and F) Confocal microscopy images obtained using an *ATXN3* protein-specific antibody (E) or a polyQ-specific antibody (F) in wild-type HEK293FT cells, mutant HEK293FT-*ATXN3*-Q84 cells, and mutant HEK293FT-*ATXN3*-Q84 cells treated with La-twin-PE. The first and second rows depict the quantification and intracellular distribution of the *ATXN3* protein (E) and polyQ inclusions (F). The third row shows DAPI-stained images of each cell, and the fourth row presents merged images of the second and third rows. HEK293FT-WT: wild-type HEK293FT cell line; HEK293FT-*ATXN3*-Q84: mutant SCA3 model cell line; None: untreated; treatment: cells treated with La-twin-PE and the pegRNA pair for twin prime editing. Scale bar, 20 *μ*m.

Finally, to establish a conceptual basis for gene therapy in human disease using the La-twin-PE module, a proof-of-concept study targeting spinocerebellar ataxia type 3 (SCA3) was conducted (Figure 6). Spinocerebellar ataxia is a genetic disorder caused by *ATXN3* mutations and manifests in an autosomal dominant manner.^28^ Among various spinocerebellar ataxia types, SCA3 has the highest incidence in humans and is a well-characterized genetic disorder with a clear phenotypic correlation owing to polyQ expansion within a single gene (*ATXN3*).^28^^,^^29^ To date, no clinically approved and effective treatments exist for SCA3, though gene-editing-based strategies for polyQ deletion and normal-length replacement have gained increasing attention.^28^ Accordingly, we aimed to conceptually demonstrate an effective therapeutic strategy for SCA3 by applying twin-PE using the La-twin-PE module developed in this study. The twin-PE system was designed to remove the polyQ repeat by inducing splicing signal substitution-triggered exon skipping and activating an in-frame stop codon in the downstream region of the mutation, enabling the expression of a C-terminally truncated form of the *ATXN3* protein (known to exhibit reduced cytotoxicity in human cells) (Figure 6A**)**.^30^ To test this hypothesis, a human-derived mutant HEK293FT-*ATXN3*-Q84 cell line was generated (Figure S7). A donor DNA vector containing a polyQ ×84 repeat and homology arms was designed to target the polyQ ×13 region of exon 10 in *ATXN3*, and CRISPR-Cas9 was used to induce polyQ ×84 insertion (Figure S7A). The cells were subsequently cultured and selected at the single-cell level to isolate clones with precisely inserted polyQ × 84 sequences (Figure S7B). The isolated single-cell clone, HEK293FT-*ATXN3*-Q84, was validated by detecting expression of a larger *ATXN3* protein than the wild type via western blotting (Figure S7C) and confirming accurate polyQ × 84 insertion in *ATXN3* through TP-PCR (Figure S7D) and Sanger sequencing (Figure S7E). To enable efficient and precise twin-PE in the HEK293FT-*ATXN3*-Q84 cell line, various combinations of sense and antisense pegRNAs targeting the upstream and downstream regions flanking the polyQ × 84 sequence in *ATXN3* were tested (S1-AS1 to S1-AS5 and S2-AS1 to S2-AS5), and high-efficiency candidate pairs were selected (Figure S8A). Consequently, the S1-AS1 or S1-AS3 combinations showed the highest efficiency in inducing the intended gene editing (Figure S8B), and the selected combination of pegRNA and the La-twin-PE module was delivered into the mutant HEK293FT-*ATXN3*-Q84 cell line (Figures 6A and S9). When polyQ-repeat-targeted twin-PE (dual-stop-codon TAA-*attB*-TAA or TAG-*attB*-TAG insertion for early stop generation) was induced in the HEK293FT-*ATXN3*-Q84 cell line, both constructs achieved effective gene editing. Compared to the twin-PE module, La-twin-PE-based twin-PE resulted in a 1.38 ± 0.10-fold average increase in precise gene editing efficiency (Figures 6B–6D, S9A, and S9B). Following efficient twin-PE at the DNA level, elimination of polyQ repeats was confirmed at both RNA and protein levels in accordance with the newly introduced encoded genetic information. In the La-twin-PE-treated group of mutant HEK293FT-*ATXN3*-Q84 cells, where editing of the polyQ region was induced, total RNA was extracted, reverse-transcribed into cDNA, and analyzed via sequencing. As expected, exon skipping was precisely induced via removal of the splicing signal at the intron 10–11 junction (Figure 6A**)**, leading to direct joining of exon 9 to exon 11 and formation of a premature stop codon (Figures S9C–S9F). Furthermore, compared with both untreated mutant and wild-type HEK293FT cells, La-twin-PE-treated mutant HEK293FT-*ATXN3*-Q84 cells showed a marked reduction in *ATXN3* protein aggregates, confirming effective polyQ repeat elimination at the protein level (Figures 6E and 6F**)**. To extend our earlier strategy of generating non-toxic *ATXN3* variants through precise C-terminal truncation using twin-PE, we additionally pursued a polyQ replacement approach aimed at restoring expression of a full-length *ATXN3* protein harboring a physiologically normal polyQ tract (∼13 repeat length) (Figure S10). Using the optimized La-twin-PE module together with a whole-overlap form pegRNA, we attempted the insertion of polyQ tracts of varying lengths into the mutant HEK293FT-*ATXN3*-Q84 cell line (Figure S10A). Sequencing analysis revealed that, in successfully edited alleles, the donor-acceptor splice signals at the intron 9–10 and intron 10–11 junctions of the *ATXN3* gene remained intact, and shortened polyQ sequences were precisely integrated (Figures S10A and S10B). These results indicated that La-twin-PE-based twin-PE developed in this study represents an effective and fundamental therapeutic strategy for genetic disorders caused by pathogenic polyQ expansion mutations.

## Discussion

In this study, to address the limitations of the conventional twin-PE-based twin-PE system^15^ and improve large-scale genome editing efficiency and accuracy, pegRNA design was optimized within the twin-PE system, and an engineered La-twin-PE module was developed, which was comparatively validated across various genomic loci in human-derived cell lines. The conventional twin-PE system based on dual twin-PE modules has been limited by inefficiency and imprecision, primarily due to the inherent complementary ambiguity of the 3′ flap structures generated via RT-mediated pegRNA extension at each gene-specific target site.^15^^,^^18^ In contrast, the optimized twin-PE strategy verified in this study overcomes these limitations by (1) employing optimally designed complementary flap structures and (2) utilizing a dual La domain-fused prime editor module, thereby enabling efficient and precise large-scale genome editing across a broad range of genomic loci. Notably, editing outcomes were strongly influenced by the degree of complementarity between pegRNAs, regardless of the target sequence type at the editing site. In this study, “whole overlap” and “partial overlap” pegRNA designs demonstrated significantly higher editing efficiencies than those containing extended homology arms. When combined with twin-PEn or La-twin-PEn modules that induce DNA double-strand breaks, these pegRNAs substantially reduced random insertion and deletion (indel) frequency. In twin-PE experiments comparing the three pegRNA types (whole overlap, homology arm, and partial overlap), the whole overlap design enabled high-efficiency and high-precision editing with both twin-PE and twin-PEn modules, demonstrating the importance of pegRNA design tailored to each DNA target. The improved twin-PE technology using the La-domain-fused twin-PE module showed enhanced large-scale genome editing efficiency based on pegRNA complementarity compared with the conventional twin-PE system. These differences are likely attributable to variations in the recruitment of DNA repair proteins during twin-PE initiated by either the twin-PEn or twin-PE modules, as well as to distinct 3′-end structures formed depending on the complementarity between paired pegRNAs. Together, these factors may differentially influence repair pathway engagement and ultimately lead to the observed discrepancies in editing outcomes. Notably, application of the La-twin-PE module to 13 genomic target sites in human-derived cell lines such as HEK293FT resulted in a large fold increase in precise editing efficiency, with unintended indel formation frequency comparable to that of the conventional twin-PE module, ensuring predictable gene editing outcomes. The effect of the La domain on twin-PE efficiency was clearly validated through comparative experiments with epegRNAs. The observation that the enhancement provided by the La domain is diminished when epegRNAs are used suggests that the 3′ pseudoknot of the epegRNA and the La-domain binding to the 3′-end of the pegRNA exert competing influences. Both appear to function through similar mechanisms involving protection of the 3′ terminus from exonucleolytic degradation, thereby leading to mutually antagonistic effects on editing efficiency. The results obtained in this study suggest that the La domain may contribute to the function or stability of the individual prime editor components,^13^ improving overall twin-PE efficiency and highlighting the importance of structural improvements via prime editor engineering for efficient large-scale genome editing. In addition to enhanced editing properties, La-twin-PE-module-induced twin-PE achieved high-precision gene editing without meaningful off-target effects. The improved gene editing precision relative to stochastic DNA misediting events observed in other engineered CRISPR-Cas modules^31^^,^^32^^,^^33^ possibly stems from the inherent property of twin-PE, requiring complementary binding between two pegRNAs to initiate editing.^15^^,^^16^ Based on the enhanced La-twin-PE module, we induced successful insertion of approximately 2.8 kb of GFP into target DNA using La-twin-PE-based twin-PE for efficient *attB* insertion, followed by Bxb1-recombinase-mediated integration. Compared with existing CRISPR-based technologies, this approach yielded greater precision and large-scale gene insertion efficiency, enhancing one-stop genome editing via twin-PE -recombinase integration. In particular, during large donor vector insertion via Bxb1 following *attB* insertion based on twin-PE, the La-twin-PE module maintained stable insertion efficiency without increasing unintended indel formation compared to the conventional twin-PE module, demonstrating superior genome integrity. Furthermore, this study demonstrated the potential of gene therapy using twin-PE via a proof-of-concept experiment in SCA3 (HEK293FT-*ATXN3*-Q84) cell lines. In conventional SCA3 models, pathogenesis is driven by polyQ repeat expansion in *ATXN3*.^28^^,^^29^ This study demonstrated precise elimination of the excessively expanded polyQ tract by inducing exon 10 skipping and activating an in-frame stop codon (downstream of the polyQ region in exon 11) to generate truncated form of *ATXN3* protein without donor DNA. The La-twin-PE module yielded a notable increase in editing efficiency compared with twin-PE and exhibited a high proportion of precise editing. These findings indicated that the optimized La-twin-PE-based twin-PE system can effectively eliminate pathogenic repeat mutation sequences at the DNA level, mitigating mutation-induced cytotoxicity and offering a fundamental therapeutic strategy for genetic disorders caused by single-gene mutations such as SCA3.

A limitation of this study is that all experiments were conducted at the *in vitro* level using human-derived cell lines, leaving potential concerns such as off-target effects, for which the analysis in this study was limited to predicted sites obtained using *in silico* prediction (http://www.rgenome.net/cas-offinder/) and did not include unbiased genome-wide profiling, immune responses, or delivery efficiency unresolved for *in vivo* or clinical applications. Additionally, the stability and potential toxicity of the La domain during long-term expression in cells require further investigation. In future studies, further development of split-Cas9 systems will likely be required to enable efficient *in vivo* editing using the optimized La-twin-PE module developed in this study, particularly through AAV-based delivery into patient-derived primary cells or mouse models. In addition, further optimization of twin-PE activity, improvement of delivery systems, and comprehensive long-term safety assessments of large-scale gene insertion will be required. Collectively, this study presents an improved strategy for large-scale genome editing through enhanced efficiency and accuracy of twin-PE. In particular, pegRNA design optimization and recombinase-based strategies applied to La-twin-PE-based twin-PE demonstrate strong potential as a core technology for application in genome engineering, disease model development, and gene therapy. These technological advancements are expected to support therapeutic strategies for diseases requiring large-scale gene modifications (e.g., hemophilia and sickle cell anemia) and facilitate research involving the insertion of large reporter genes.

## Methods

### Cloning of prime editor and pegRNA expression vectors

All protein expression vectors, including SpCas9(H840A)-RT, La-SpCas9(H840A)-RT, SpCas9(WT)-RT, and La-SpCas9(WT)-RT, were constructed under the control of the cytomegalovirus (CMV) promoter and subcloned using Gibson Assembly Master Mix (New England Biolabs, E2611L). Paired pegRNA expression plasmids encoding the target sequence, primer binding site (PBS), and RTT regions were constructed under the control of a U6 promoter (Table S1). Target sequences were inserted into the pegRNA backbone via Golden Gate cloning using T4 ligase (NEB, M0202L) and dsDNA fragments with overhangs complementary to BsaⅠ-digested sites in the backbone vector. Digestion was performed using BsaⅠ, followed by ligation of a target-containing ssDNA fragment synthesized from Macrogen and annealed via gradual cooling. PBS and RTT sequences were inserted by digesting the previously modified pegRNA backbone vector with BsmBⅠ and ligating dsDNA fragments containing the PBS and RTT regions using the same method.

### Mammalian cell culture and transfection

HEK293FT cells (R70007; Invitrogen) and HeLa cells (CCL-2; American Type Culture Collection) were cultured in Dulbecco’s modified Eagle’s medium (DMEM; Gibco, 11995065) supplemented with 10% fetal bovine serum (FBS; Gibco, 16000044) and 1 × penicillin-streptomycin (Welgene, LS202-02) at 37°C in a humidified incubator containing 5% CO_2_. Mutant HEK293FT-*ATXN3*-Q84 cells were maintained under identical conditions. HEK293FT and HEK293FT-*ATXN3*-Q84 were seeded in 48-well plates (Corning, 3548) under the same conditions as previously described. Transfection was performed at ∼60% confluency after 16–20 h of incubation. For twin-PE, 750 ng of SpCas9(H840A)-RT, La-SpCas9(H840A)-RT, SpCas9(WT)-RT, or La-SpCas9(WT)-RT and 125 ng of each pegRNA expression vector were used. For donor-mediated GFP insertion, 500 ng of SpCas9(H840A)-RT or La-SpCas9(H840A)-RT, 50 ng of each pegRNA vector, 200 ng of CMV-Bxb1 (Addgene #182142), and 200 ng of donor plasmid were co-transfected. All plasmids used in this study were purified using Nucleobond Xtra Midi Plus (MN740412.5). Transfections were performed using a mixture of 0.5 μL P3000 and 0.75 μL Lipofectamine 3000 (Thermo Fisher Scientific, L3000008) in 25 μL Opti-MEM (Gibco, 31985062) according to the manufacturer’s protocol.

### Genomic DNA extraction and amplicon preparation

Genomic DNA was extracted 72 h post-plasmid transfection using the AccuPrep Genomic DNA Extraction Kit (Bioneer, K-3032) following the manufacturer’s instructions. Target genomic loci were amplified via primary PCR using KOD One Master Mix (Toyobo, KMM-101) and primers listed in Table S2. PCR was conducted under the following cycling conditions: 98°C for 2 min; 30 cycles at 98°C for 10 s, 60°C for 10 s, 68°C for 10 s; and a final extension at 68°C for 5 min. Amplicons were further processed using nested PCR with Phusion High-Fidelity DNA (NEB, M0530L) to append Illumina sequencing adapters and barcodes (Table S2) under the following conditions: 98°C for 5 min; 30 cycles at 98°C for 10 s, 62°C for 10 s, 72°C for 10 s; and 72°C for 5 min. PCR products were verified via 2% agarose gel electrophoresis and purified using a QIAquick PCR Purification Kit (Qiagen, 28106).

### Targeted amplicon sequencing and data analysis

Cas-OFFinder^27^ (http://www.rgenome.net/cas-offinder/) predicted genome-wide candidate off-target sites derived from the target sequence (Table S3). To evaluate potential off-target mutations via twin-prime editing, PCR was performed on the on-target site and the top 10 prioritized candidate (allowing up to three mismatches) off-target genome sites. Amplification products containing mutations (on- or off-target) were analyzed via next-generation sequencing (NGS) with barcoded nested PCR (denaturation at 98°C for 30 s, primer annealing at 62°C for 30 s, and elongation at 72°C for 30 s, 35 cycles) using Phusion High-Fidelity DNA Polymerase (NEB, M0530L). Thereafter, the barcoded amplicon mixture was purified using the QIAquick PCR Purification Kit (Qiagen, 28106). Purified amplicons were loaded onto the MiniSeq analyzer (Illumina MiniSeq system, SY-420-1001), and targeted deep sequencing was performed according to the manufacturer’s protocol. The detection threshold (>0.1%) corresponded to the typical background error rate of amplicon sequencing, and the observed indel frequencies at predicted off-target sites were comparable to those detected in untreated control samples for each site. FASTQ data were analyzed using Cas-analyzer (http://www.rgenome.net/cas-analyzer/). Frequencies of precise twin-PE, indels only, and partial insertions (%) were calculated as a proportion of total allele frequency (frequency of twin-PE, indels only, and partial insertions [%]/total allele frequency [%]) and plotted using GraphPad Prism (v.10.4.1).

### Generation and characterization of the mutant HEK293FT-*ATXN3-*Q84 clonal cell line

HEK293FT cells (Invitrogen, R70007) were seeded in 24-well plates (Corning) and transfected at ∼60% confluency after 16–20 h. Transfection used 1 μg of a homemade Cas9-P2A-Blasticidin vector, 500 ng of each sgRNA1 (5′-TCCCAAAGTGCTGGGATTACAGG-3′) and sgRNA2 (5′-GTATGTCAGATAAAGTGTGAAGG-3′) vectors, and 1 μg donor DNA containing a Q84-expanded *ATXN3* exon 10 coding region flanked by 823 bp left and 807 bp right homology arms. Transfection was conducted via electroporation using an Amaxa electroporation kit (V4XC-2032; program: CM-130). After 3 days, the medium was replaced with fresh medium containing 10 μg/mL Blasticidin S, refreshed every 2 days. After 5 days of selection, surviving clones were subjected to limiting dilution and plated into 96-well plates for single-cell culture. Genomic DNA was extracted using the AccuPrep Genomic DNA Extraction Kit (Bioneer, K-3032), and successful insertion was confirmed via PCR and agarose gel electrophoresis.

### Triplet repeat-primed PCR

To detect CAG expansions in *ATXN3* exon 10, TP-PCR was performed following the method of Mulias et al. The 50 μL reaction included Phusion High-Fidelity DNA Polymerase (NEB, M0530L), 10 ng genomic DNA or 1 pg donor plasmid, 1 μM forward and tailing primers, and 0.5 μM reverse primer (Table S2). Cycling conditions were as follows: 95°C for 15 min; 40 cycles at 98°C for 45 s, 60°C for 1 min, and 72°C for 2 min, followed by 72°C for 5 min. PCR products were analyzed via fragment analysis at Macrogen and quantified using GeneMarker v.2.6.3 (SoftGenetics).

### Western blot analysis

To assess polyQ repeat expression from *ATXN3* exon 10, HEK293FT and HEK293FT-*ATXN3*-Q84 cells were harvested via scraping. Cell pellets were lysed in PRO-PREP buffer (Intron Biotechnology, 17081) following the manufacturer’s protocol. Equal amounts of total protein were resolved using 10% SDS-PAGE at 100 V for 2 h, transferred to nitrocellulose membranes (Bio-Rad, 1620115) at 0.3 A for 3 h, and blocked with 5% skim milk in blocking buffer (Biosesang, TR2005-100-74). Membranes were incubated overnight at 4°C with anti-SCA3 (Sigma-Aldrich, MAB5360) and anti-β-actin (Santa Cruz, sc-47778) primary antibodies. After washing with TBST (0.1% Tween 20 in TBS), the HRP-conjugated goat anti-mouse secondary antibody was applied and incubated overnight at 4°C. Detection was performed using enhanced chemiluminescence reagent (CYANGEN, Cod. XLS075,0100) and visualized with the Chemi DocXRS+ system (Bio-Rad, Model No. Universal Hood II). Densitometry was analyzed using Image Lab software v.3.0 (Bio-Rad, 12012931).

### RNA extraction and cDNA synthesis

Total RNA was extracted from HEK293FT-WT, HEK293FT-*ATXN3*-Q84, and HEK293FT-*ATXN3*-Q84 cells treated with La-twin-PE at 72 h post-transfection using the RNeasy Mini Kit (Qiagen, 74104) according to the manufacturer’s instructions. RNA quantity and purity were assessed using a NanoDrop One spectrophotometer (Thermo Fisher Scientific, Waltham, MA, USA, ND-ONE-C). For cDNA synthesis, 1 μg RNA from each sample was used with the PrimeScript RT reagent Kit with gDNA Eraser (Takara, RR047A).

### Immunocytochemistry

To investigate the intracellular distribution of polyQ inclusions and *ATXN3* protein in HEK293FT-WT, HEK293FT-*ATXN3*-Q84, and HEK293FT-*ATXN3*-Q84 cells treated with La-twin-PE, cells were seeded on poly-D-lysine-coated coverslips and incubated for 48 h. Thereafter, cells were washed with PBS and fixed with 4% paraformaldehyde for 2 h at room temperature. Fixed cells were subsequently washed with PBS (Biosesang, PR2004-100-72) and permeabilized using 0.25% PBST (PBS with 0.25% Triton X-100). Subsequently, each cell sample was blocked with PBST containing 1% BSA (MP Biomedicals, 160069) for 30 min to reduce non-specific binding of primary antibodies. To detect polyQ inclusions and *ATXN3* protein in each cell group, the samples were incubated overnight at 4°C with either the anti-Polyglutamine-Expansion Disease Marker (Sigma-Aldrich, clone 5TF1-1C2, MAB1574) or the anti-Spinocerebellar Ataxia Type 3 antibodies (Sigma-Aldrich, clone 1H9, MAB5360), diluted in 1% BSA. After removing the primary antibody, coverslips were washed with PBS and incubated for 1 h at room temperature with the secondary antibody, Goat Anti-mouse IgG H&L (Abcam, Alexa Fluor 488, ab150113). Following secondary antibody incubation, cells were washed with PBS and mounted using antifade mounting medium containing DAPI (VECTASHIELD, H-1200-10) for nuclear counterstaining. Images were acquired using a confocal microscope (Carl Zeiss, Meditec, Dublin, CA, USA, LSM-700), and 3D surface plots of polyQ inclusions were generated using ImageJ software (NIH, Bethesda, MD, USA).

## Data and code availability

All relevant data that support the study findings are available from the corresponding author upon request. All targeted amplicon sequencing data were deposited in the NCBI Sequence Read Archive database with accession numbers PRJNA1281332 and SUB15409413.

## Acknowledgments

This research was supported by grants from the 10.13039/501100001321National Research Foundation (NRF) funded by the 10.13039/501100004085Korean Ministry of Education, Science and Technology (RS-2025-00554011, RS-2025-02218918, and RS-2022-NR071772). This study was also supported by a grant of the Korea Health Technology R&D Project through the 10.13039/501100003710Korea Health Industry Development Institute (KHIDI), funded by the Ministry of Health & Welfare, Republic of Korea (grant number: RS-2024-00439579) and grants from the Korea Research Institute of Bioscience and Biotechnology (10.13039/501100003715KRIBB; Research Initiative Program KGM4562532 and KGM1072511).

## Author contributions

Conceptualization, L.W.G., J.B.S., Y.O., and S.H.L.; methodology, L.W.G., J.B.S., Y.O., K.-H.P., and S.H.L.; software, L.W.G., Y.O., K.-H.P., and S.H.L.; validation, L.W.G., J.B.S., Y.O., and S.H.L.; formal analysis, L.W.G. and S.H.L.; investigation, L.W.G. and S.H.L.; resources, L.W.G., J.B.S., Y.O., Y.-H.K., J.-w.H., Y.L., and S.H.L.; data curation, L.W.G. and S.H.L.; writing – original draft, L.W.G. and S.H.L.; writing – review and editing, L.W.G., Y.L., and S.H.L.; visualization, L.W.G. and S.H.L.; supervision and project administration, Y.L. and S.H.L.; funding acquisition, K.-H.P., Y.L., and S.H.L.

## Declaration of interests

The authors declare no competing interests.

## Supplemental information

Document S1. Figures S1–S10

Table S1. Nucleotide sequences of pegRNAs used for twin prime editing at each DNA target in this study This table is provided as a separate Excel file. (Table_S1.xlsx)

Table S2. Nucleotide sequences of DNA primers used in this study This table is provided as a separate Excel file. (Table_S2.xlsx)

Table S3. *In silico*-predicted off-target candidate sequences corresponding to each target DNA sequence analyzed in this study This table is provided as a separate Excel file. (Table_S3.xlsx)

Document S2. Article plus supplemental information

## References

[1] Pacesa M., Pelea O., Jinek M. (2024). Past, present, and future of CRISPR genome editing technologies. Cell.

[2] Jinek M., Chylinski K., Fonfara I., Hauer M., Doudna J.A., Charpentier E. (2012). A programmable dual-RNA-guided DNA endonuclease in adaptive bacterial immunity. Science.

[3] Anzalone A.V., Koblan L.W., Liu D.R. (2020). Genome editing with CRISPR-Cas nucleases, base editors, transposases and prime editors. Nat. Biotechnol..

[4] Anzalone A.V., Randolph P.B., Davis J.R., Sousa A.A., Koblan L.W., Levy J.M., Chen P.J., Wilson C., Newby G.A., Raguram A., Liu D.R. (2019). Search-and-replace genome editing without double-strand breaks or donor DNA. Nature.

[5] Chen P.J., Liu D.R. (2023). Prime editing for precise and highly versatile genome manipulation. Nat. Rev. Genet..

[6] Zeng H., Daniel T.C., Lingineni A., Chee K., Talloo K., Gao X. (2024). Recent advances in prime editing technologies and their promises for therapeutic applications. Curr. Opin. Biotechnol..

[7] Hew B.E., Gupta S., Sato R., Waller D.F., Stoytchev I., Short J.E., Sharek L., Tran C.T., Badran A.H., Owens J.B. (2024). Directed evolution of hyperactive integrases for site specific insertion of transgenes. Nucleic Acids Res..

[8] Pandey S., Gao X.D., Krasnow N.A., McElroy A., Tao Y.A., Duby J.E., Steinbeck B.J., McCreary J., Pierce S.E., Tolar J. (2025). Efficient site-specific integration of large genes in mammalian cells via continuously evolved recombinases and prime editing. Nat. Biomed. Eng..

[9] Yarnall M.T.N., Ioannidi E.I., Schmitt-Ulms C., Krajeski R.N., Lim J., Villiger L., Zhou W., Jiang K., Garushyants S.K., Roberts N. (2023). Drag-and-drop genome insertion of large sequences without double-strand DNA cleavage using CRISPR-directed integrases. Nat. Biotechnol..

[10] Shuto Y., Nakagawa R., Zhu S., Hoki M., Omura S.N., Hirano H., Itoh Y., Zhang F., Nureki O. (2024). Structural basis for pegRNA-guided reverse transcription by a prime editor. Nature.

[11] Chen P.J., Hussmann J.A., Yan J., Knipping F., Ravisankar P., Chen P.F., Chen C., Nelson J.W., Newby G.A., Sahin M. (2021). Enhanced prime editing systems by manipulating cellular determinants of editing outcomes. Cell.

[12] Doman J.L., Pandey S., Neugebauer M.E., An M., Davis J.R., Randolph P.B., McElroy A., Gao X.D., Raguram A., Richter M.F. (2023). Phage-assisted evolution and protein engineering yield compact, efficient prime editors. Cell.

[13] Yan J., Oyler-Castrillo P., Ravisankar P., Ward C.C., Levesque S., Jing Y., Simpson D., Zhao A., Li H., Yan W. (2024). Improving prime editing with an endogenous small RNA-binding protein. Nature.

[14] Nelson J.W., Randolph P.B., Shen S.P., Everette K.A., Chen P.J., Anzalone A.V., An M., Newby G.A., Chen J.C., Hsu A., Liu D.R. (2022). Engineered pegRNAs improve prime editing efficiency. Nat. Biotechnol..

[15] Anzalone A.V., Gao X.D., Podracky C.J., Nelson A.T., Koblan L.W., Raguram A., Levy J.M., Mercer J.A.M., Liu D.R. (2022). Programmable deletion, replacement, integration and inversion of large DNA sequences with twin prime editing. Nat. Biotechnol..

[16] Awan M.J.A., Ali Z., Amin I., Mansoor S. (2022). Twin prime editor: seamless repair without damage. Trends Biotechnol..

[17] Steinbeck B.J., Gao X.D., McElroy A.N., Pandey S., Doman J.L., Riddle M.J., Xia L., Chen W., Eide C.R., Lengert A.H. (2024). Twin Prime Editing Mediated Exon Skipping/Reinsertion for Restored Collagen VII Expression in Recessive Dystrophic Epidermolysis Bullosa. J. Invest. Dermatol..

[18] Tao R., Wang Y., Jiao Y., Hu Y., Li L., Jiang L., Zhou L., Qu J., Chen Q., Yao S. (2022). Bi-PE: bi-directional priming improves CRISPR/Cas9 prime editing in mammalian cells. Nucleic Acids Res..

[19] Choi J., Chen W., Suiter C.C., Lee C., Chardon F.M., Yang W., Leith A., Daza R.M., Martin B., Shendure J. (2022). Precise genomic deletions using paired prime editing. Nat. Biotechnol..

[20] Gwon L.W., Badon I.W., Lee Y., Kim H.J., Lee S.H. (2025). Advances in large-scale DNA engineering with the CRISPR system. Exp. Mol. Med..

[21] Han J.P., Kim M., Choi B.S., Lee J.H., Lee G.S., Jeong M., Lee Y., Kim E.A., Oh H.K., Go N. (2022). In vivo delivery of CRISPR-Cas9 using lipid nanoparticles enables antithrombin gene editing for sustainable hemophilia A and B therapy. Sci. Adv..

[22] Nguyen T.H., Anegon I. (2016). Successful correction of hemophilia by CRISPR/Cas9 genome editing in vivo: delivery vector and immune responses are the key to success. EMBO Mol. Med..

[23] Park C.Y., Kim D.H., Son J.S., Sung J.J., Lee J., Bae S., Kim J.H., Kim D.W., Kim J.S. (2015). Functional Correction of Large Factor VIII Gene Chromosomal Inversions in Hemophilia A Patient-Derived iPSCs Using CRISPR-Cas9. Cell Stem Cell.

[24] Ling S., Zhang X., Dai Y., Jiang Z., Zhou X., Lu S., Qian X., Liu J., Selfjord N., Satir T.M. (2025). Customizable virus-like particles deliver CRISPR-Cas9 ribonucleoprotein for effective ocular neovascular and Huntington's disease gene therapy. Nat. Nanotechnol..

[25] Ekman F.K., Ojala D.S., Adil M.M., Lopez P.A., Schaffer D.V., Gaj T. (2019). CRISPR-Cas9-Mediated Genome Editing Increases Lifespan and Improves Motor Deficits in a Huntington's Disease Mouse Model. Mol. Ther. Nucleic Acids.

[26] Adikusuma F., Lushington C., Arudkumar J., Godahewa G.I., Chey Y.C.J., Gierus L., Piltz S., Geiger A., Jain Y., Reti D. (2021). Optimized nickase- and nuclease-based prime editing in human and mouse cells. Nucleic Acids Res..

[27] Bae S., Park J., Kim J.S. (2014). Cas-OFFinder: a fast and versatile algorithm that searches for potential off-target sites of Cas9 RNA-guided endonucleases. Bioinformatics.

[28] Cui Z.T., Mao Z.T., Yang R., Li J.J., Jia S.S., Zhao J.L., Zhong F.T., Yu P., Dong M. (2024). Spinocerebellar ataxias: from pathogenesis to recent therapeutic advances. Front. Neurosci..

[29] Matos C.A., de Almeida L.P., Nóbrega C. (2019). Machado-Joseph disease/spinocerebellar ataxia type 3: lessons from disease pathogenesis and clues into therapy. J. Neurochem..

[30] Toonen L.J.A., Rigo F., van Attikum H., van Roon-Mom W.M.C. (2017). Antisense Oligonucleotide-Mediated Removal of the Polyglutamine Repeat in Spinocerebellar Ataxia Type 3 Mice. Mol. Ther. Nucleic Acids.

[31] Zhang L., Zuris J.A., Viswanathan R., Edelstein J.N., Turk R., Thommandru B., Rube H.T., Glenn S.E., Collingwood M.A., Bode N.M. (2021). AsCas12a ultra nuclease facilitates the rapid generation of therapeutic cell medicines. Nat. Commun..

[32] Kim H., Lee W.J., Kim C.H., Oh Y., Gwon L.W., Lee H., Song W., Hur J.K., Lim K.S., Jeong K.J. (2022). Highly specific chimeric DNA-RNA-guided genome editing with enhanced CRISPR-Cas12a system. Mol. Ther. Nucleic Acids.

[33] Kleinstiver B.P., Sousa A.A., Walton R.T., Tak Y.E., Hsu J.Y., Clement K., Welch M.M., Horng J.E., Malagon-Lopez J., Scarfò I. (2019). Engineered CRISPR-Cas12a variants with increased activities and improved targeting ranges for gene, epigenetic and base editing. Nat. Biotechnol..

