# Supplementary material for: Optimizing twin prime editing components for scalable genome editing and therapy in spinocerebellar ataxia type 3
Source: Mol Ther Nucleic Acids. 2026 Jun 17;37(3):102988. doi: 10.1016/j.omtn.2026.102988 (PMC13330521; doi:10.1016/j.omtn.2026.102988)
Supplement: Document S1. Figures S1–S10 [file mmc1.pdf]

OMTN, Volume *37*

Supplemental information

Optimizing twin prime editing components for scalable genome editing and therapy in spinocerebellar ataxia type 3

Lee Wha Gwon, Jung Bae Seong, Hyeon-Gu Yeo, Yeounsun Oh, Junghyung Park, Jinyoung Won, Sang Je Park, Young-Hyun Kim, Jae-won Huh, Aryun Kim, Kwang-Hyun Park, Youngjeon Lee, and Seung Hwan Lee

SUPPLEMENTAL FIGURES

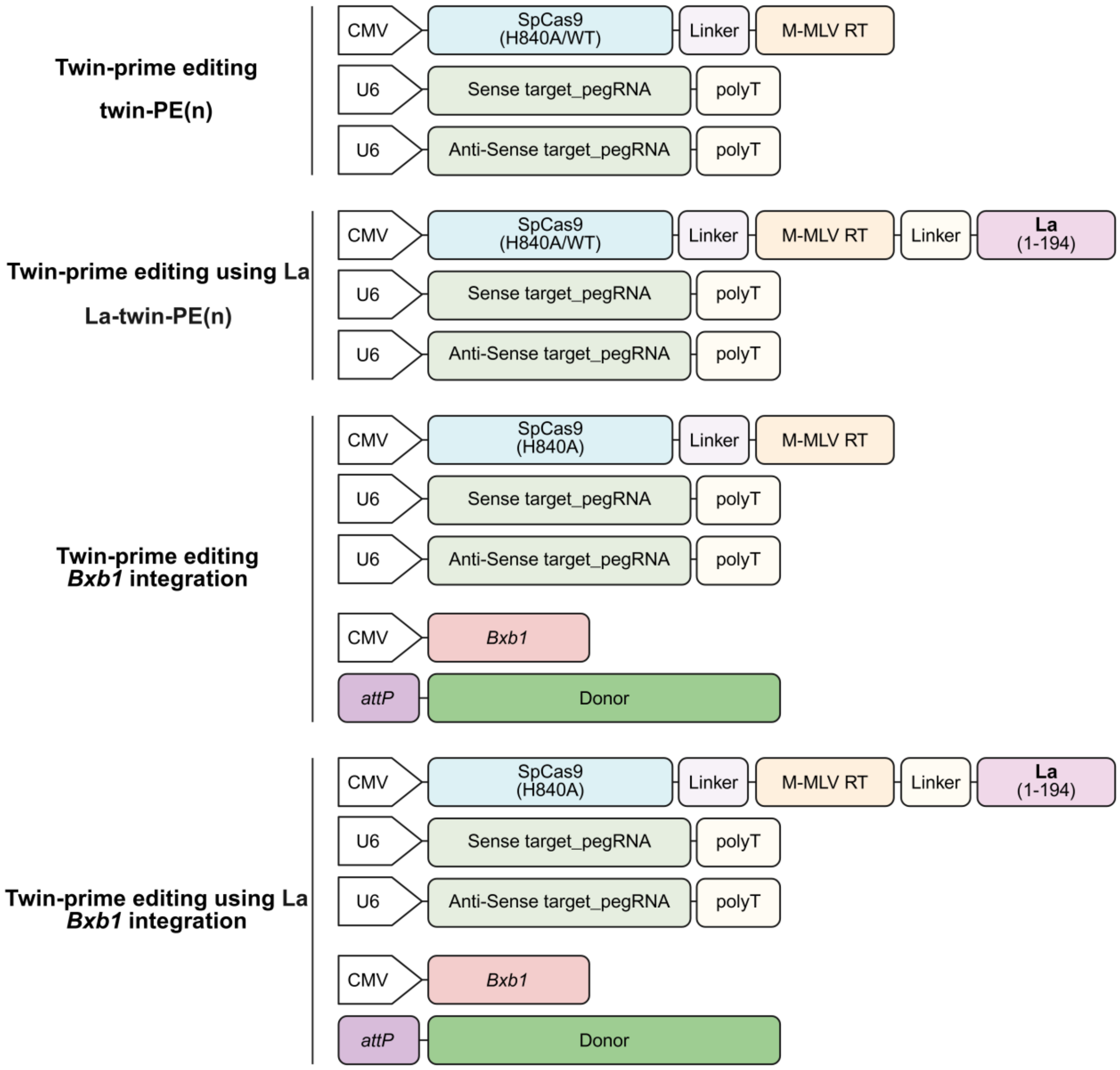

Figure S1. Method for inducing twin prime editing using various prime editor expression plasmid combinations. To induce target-specific twin prime editing in human-derived cells, expression vectors based on the CMV promoter were constructed for twin-PE(n) or La-twin-PE(n). For expression of sense and antisense pegRNAs with target DNA-specific PBS+RTT sequences, U6 promoter-based vectors were also used. For La-twin-PE(n) expression, a minimal N-terminus (1–194 aa) of the La protein was fused via a linker to the WT or nickase SpCas9 module. For

experiments involving *attB* insertion and subsequent donor DNA recombination via Bxb1 recombinase in human-derived cells, vectors expressing twin-PE(n) or La-twin-PE(n) were used with the same twin prime editing strategy, along with CMV promoter-based Bxb1 expression vectors and donor DNA vectors containing the *attP* recognition sequence. MMLV-RT: Reverse transcriptase domain from Moloney Murine Leukemia Virus; pegRNA: prime editing guide RNA; La (1–194): La protein N-terminal domain (1–194 aa); Bxb1: Bxb1 recombinase; *attB*/*P*: Bxb1 recognition DNA sequences.

AAVS1 target site

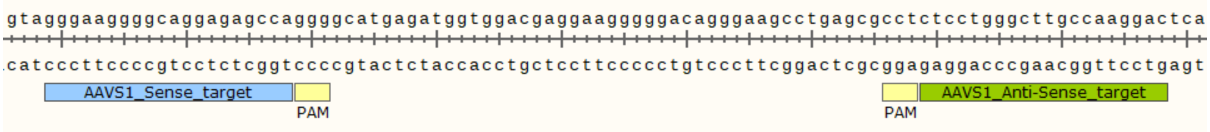

AAVS1 attB insertion

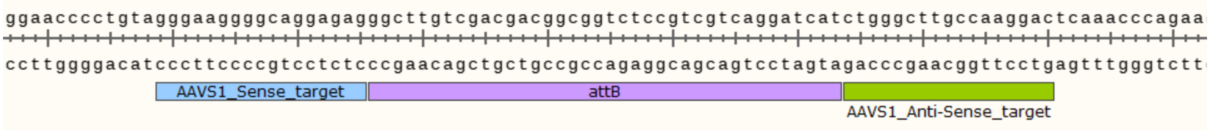

AAVS1 attP insertion

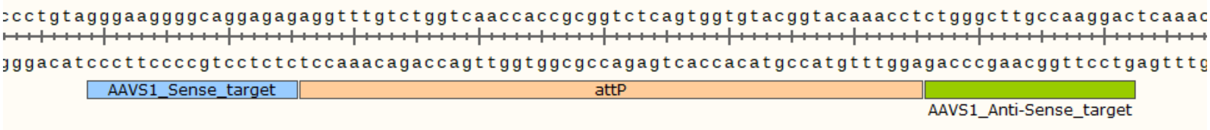

HEK3 target site

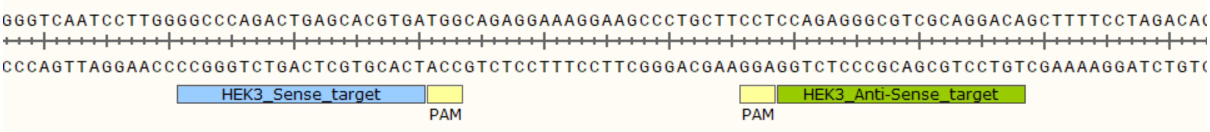

HEK3 attB insertion

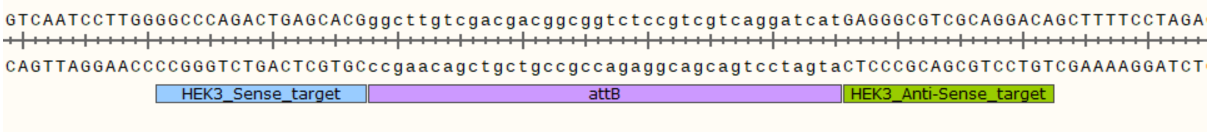

FANCF target site

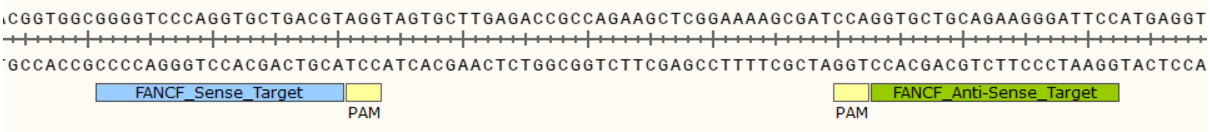

FANCF attB insertion

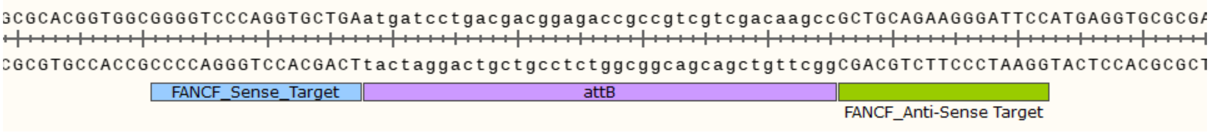

CCR5 target site

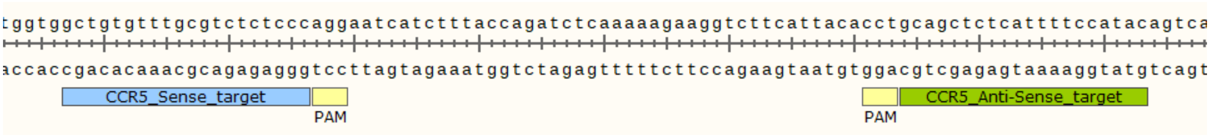

CCR5 attB insertion

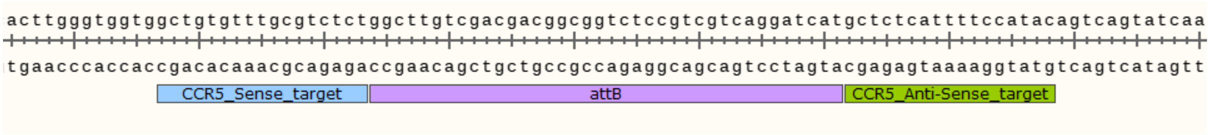

EMX1_1 target site

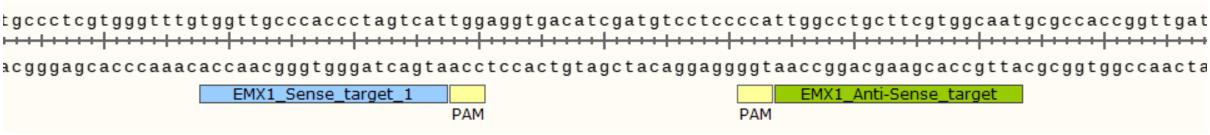

EMX1_1 attB insertion

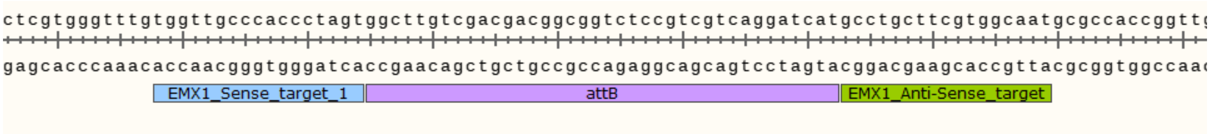

EMX1_2 target site

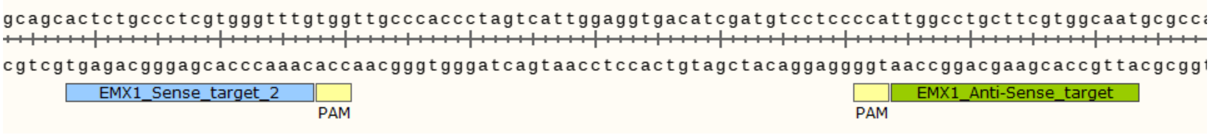

EMX1_2 attB insertion

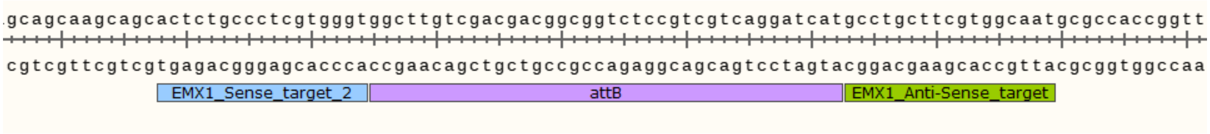

VEGFA target site

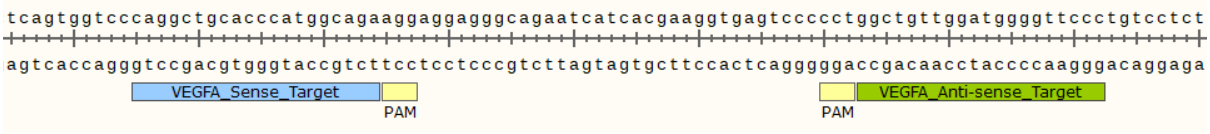

VEGFA attB insertion

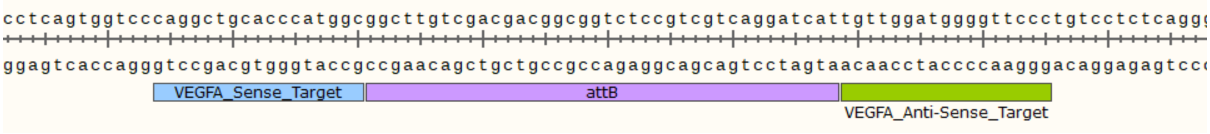

DAPK1 target site

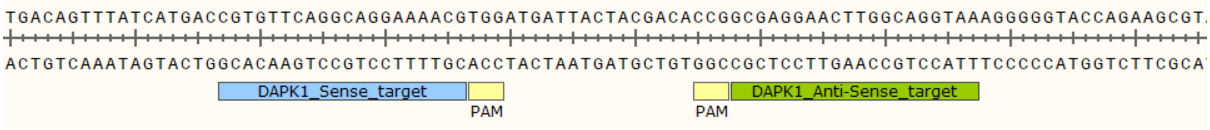

DAPK1 attB insertion

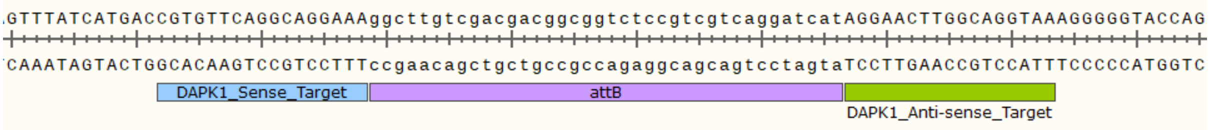

PIN1_1 target site

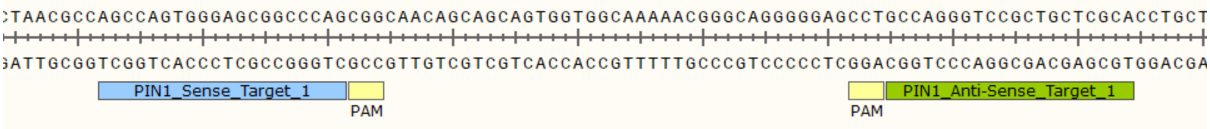

PIN1_1 attB insertion

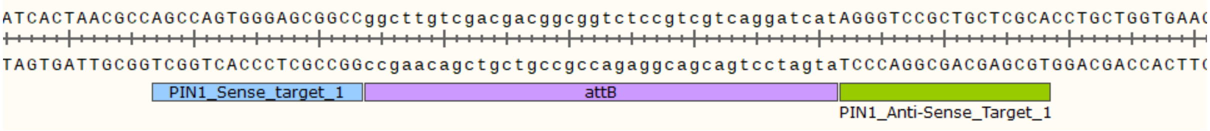

PIN1_2 target site

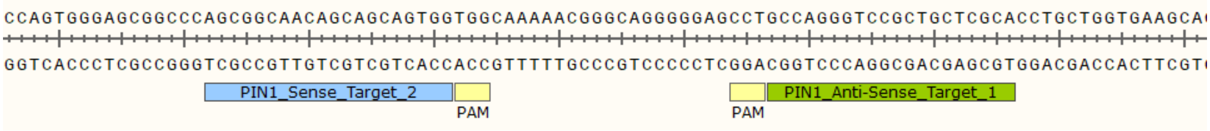

PIN1_2 attB insertion

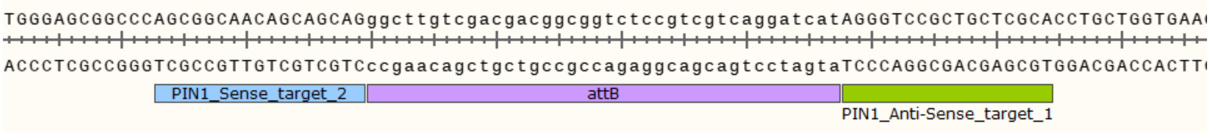

***PIN1_3* target site**

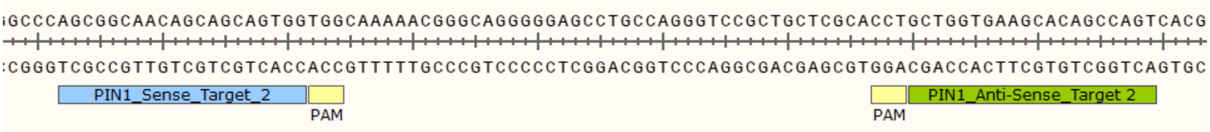

***PIN1_3 attB* insertion**

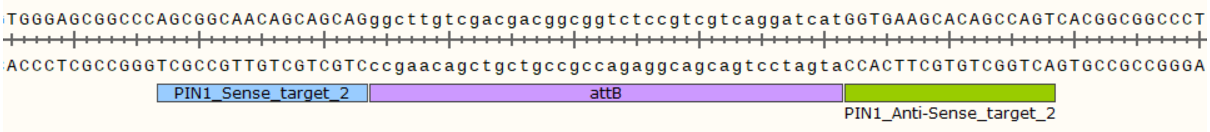

***GAPDH_1* target site**

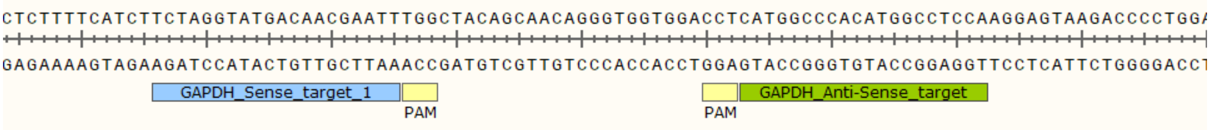

***GAPDH_1 attB* insertion**

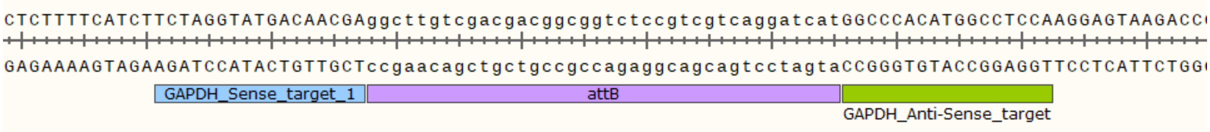

***GAPDH_2* target site**

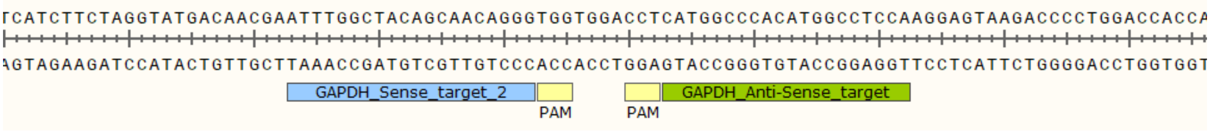

***GAPDH_2 attB* insertion**

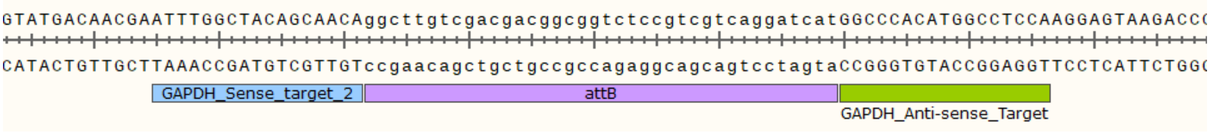

Figure S2. Twin prime editing strategy using paired pegRNAs based on SpCas9(H840A/WT)-RT or La-SpCas9(H840A/WT)-RT. Each DNA schematic illustrates the design of paired pegRNAs targeting specific sequences within the indicated genomic loci (*AAVS1, HEK3, FANCF, CCR5, EMX1, VEGFA, DAPK1, PIN1*, and *GAPDH*) and the precise insertion of *attB/attP* sequences achieved via twin-PE(n)

or La-twin-PE(n). Within the target DNA sequences, the protospacers and PAM (NGG) sequences corresponding to each sense and antisense pegRNA are colored in light blue, light green, and yellow, respectively.

AAVS1 Twin-prime editing pattern (*attB* insertion)

AAVS1

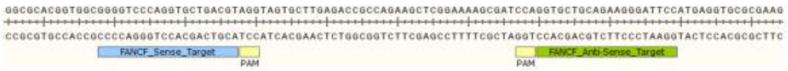

Insertion
Subtitution
Deletion : -----

Normal control GGGAAGGGGCAGGAGAGCCAGGGGCATGAGATGGTGGACGAGGAAGGGGGACAGGGAAGCCTGAGCGCCTCTCCTGGGC TTGCCAAGGAC

AAVS1 / twin-PE / Whole overlap (*attB* insertion)

Insertion
Substitution
Deletion : -----

Precise insertion GGGAAGGGGCAGGAGAGggcttgtcgacgacggcggtctccgtcgtcaggatcatCTGGGCTTGCCAAGGAC — 19.11 %
attB (38bp)

Pure indel
GGGAAGGGGCAGGAGAG--CTGGGCTTGCCAAGGAC — 0.29 %
GGGAAGGGGCAG-----------GAGATGGTGGACGAGGAAGGGGGACAGGGAAGCCTGAGCGCCTCTCCTGGGCTTGCCAAGGAC — 0.16 %
GGGA---------------AGGGGCATGAGATGGTGGACGAGGAAGGGGGACAGGGAAGCCTGAGCGCCTCTCCTGGGCTTGCCAAGGAC — 0.02 %

Partial insertion
GGGAAGGGGCAGGAGAGGGCTTggcttgtcgacgacggcggtctccgtcgtcaggatcatCTGGGCTTGCCAAGGAC — 0.26 %
GGGAAGGGGCAGGAGAGggcttgtcgacgacggcggtAC------------------------------TGGTGGACGAGGAAGGGGGACAGGGAAGCCTG AGCGCCTCTCCTGGGCTTGCCAAGGAC — 0.18 %
GGGAAGGGGCAGGAGAGCgcttgtcgacgacggcggtctccgtcgtcaggatcatCTGGGCTTGCCAAGGAC — 0.12 %
GGGAAGGGGCAGGAGAGCgcttgtcgacgacggcggtctccgtcgtcaggatcTCCTGGGCTTGCCAAGGAC — 0.12 %
GGGAAGGGGCAGGAGAGggcttgtcgacgacggcggtctccgtcgtcaggatc----------TGAGATGGTGGACGAGGAAGGGGGACAGGGAAG CCTGAGCGCCTCTCCTGGGCTTGCCAAGGAC — 0.12 %

AAVS1 / La-twin-PE / Whole overlap (*attB* insertion)

Insertion
Substitution
Deletion : -----

Precise insertion GGGAAGGGGCAGGAGAGggcttgtcgacgacggcggtctccgtcgtcaggatcatCTGGGCTTGCCAAGGAC — 24.10 %
attB (38bp)

Pure indel None

Partial insertion
GGGAAGGGGCAGGAGAGggcttgtcgacgacggc--------------------ATCTGGGCTTGCCAAGGAC — 0.55 %
GGGAAGGGGCAGGAGAG-gcttgtcgacgacggcggtctccgtcgtcaggatcatCTGGGCTTGCCAAGGAC — 0.24 %
GGGAAGGGGCAGGAGAGggcttgt---cgtcaggatcatCT GGGCTTGCCAAGGAC — 0.18 %
Sense insertion / Anti-Sense insertion
GGGAAGGGGCAGGAGAGCCAGGGGCA--GGAGAGggcttgtcgacgacggcggtctccgtcgtcaggatca tCTGGGCTTGCCAAGGAC — 0.18 %
GGGAAGGGGCAGGAGAGggcttgtcgacgac--------------------------GCATGAGATGGTGGACGAGGAAGGGGGACAGGGAAGCCTGAGC GCCTCTCCTGGGCTTGCCAAGGAC — 0.13 %

AAVS1 / twin-PEn / Whole overlap (*attB* insertion)

Insertion
Substitution
Deletion : -----

Precise insertion GGGAAGGGGCAGGAGAGggcttgtcgacgacggcggtctccgtcgtcaggatcatCTGGGCTTGCCAAGGAC — 10.35 %
attB (38bp)

Pure indel
GGGAAGGGGCAGGAGAG--CTGGGCTTGCCAAGGAC — 1.18 %
GGGAAGGGGCAGGA--TCATCTGGGCTTGCCAAGGAC — 1.57 %
GGGAAGGGGCAGGAGAG--CCTGGGCTTGCCAAGGAC — 0.52 %
GGGAAGGGGCAGGAGAGCCAGGGGCATGAGATGGTGGACGAGGAAGGGGGACAGGGAAGCCTGAGCGCCTCTCCATCTGGGC TTGCCAAGGAC — 0.48 %
GGGAAGGGGCAGGAGAGCCAGGGGCATGAGATGGTGGACGAGGAAGGGGGACAGGGAAGCCTGAGCGCCTCTCCCTGGGC TTGCCAAGGAC — 0.43 %

Partial insertion
GGGAAGGGGCAGGAGAGggcttgtcgacgacggcggtctccgtcgtcaggatcatCCAGGGGCATGAGATGGTGGACGAGGAAGGGGGACAG GGAAGCCTGAGCGCCTCTCCTGGGCTTGCCAAGGAC — 1.30 %
GGGAAGGGGCAGGAGAGggcttgtcgacgac--------------------------CCAGGGGCATGAGATGGTGGACGAGGAAGGGGGACAG GGAAGCCTGAGCGCCTCTCCTGGGCTTGCCAAGGAC — 0.73 %
GGGAAGGGGCAGGAGAGCCAGGGGCATGAGATGGTGGACGAGGAAGGGGGACAGGGAAGCCTGAGCGCCTCTCCggcttgtcgac gacggcggtctccgtcgtcaggatcatCTGGGCTTGCCAAGGAC — 0.53 %
GGGAAGGGGCAGGAGAGggcttg----------------------------------TCCTGGGCTTGCCAAGGAC — 0.41 %
GGGAAGGGGCAGGAGAggcttgtcgacgacggcggtctccgtcgtcaggatcatGCCAGGGGCATGAGATGGTGGACGAGGAAGGGGGACAG GGAAGCCTGAGCGCCTCTCCTGGGCTTGCCAAGGAC — 0.37 %

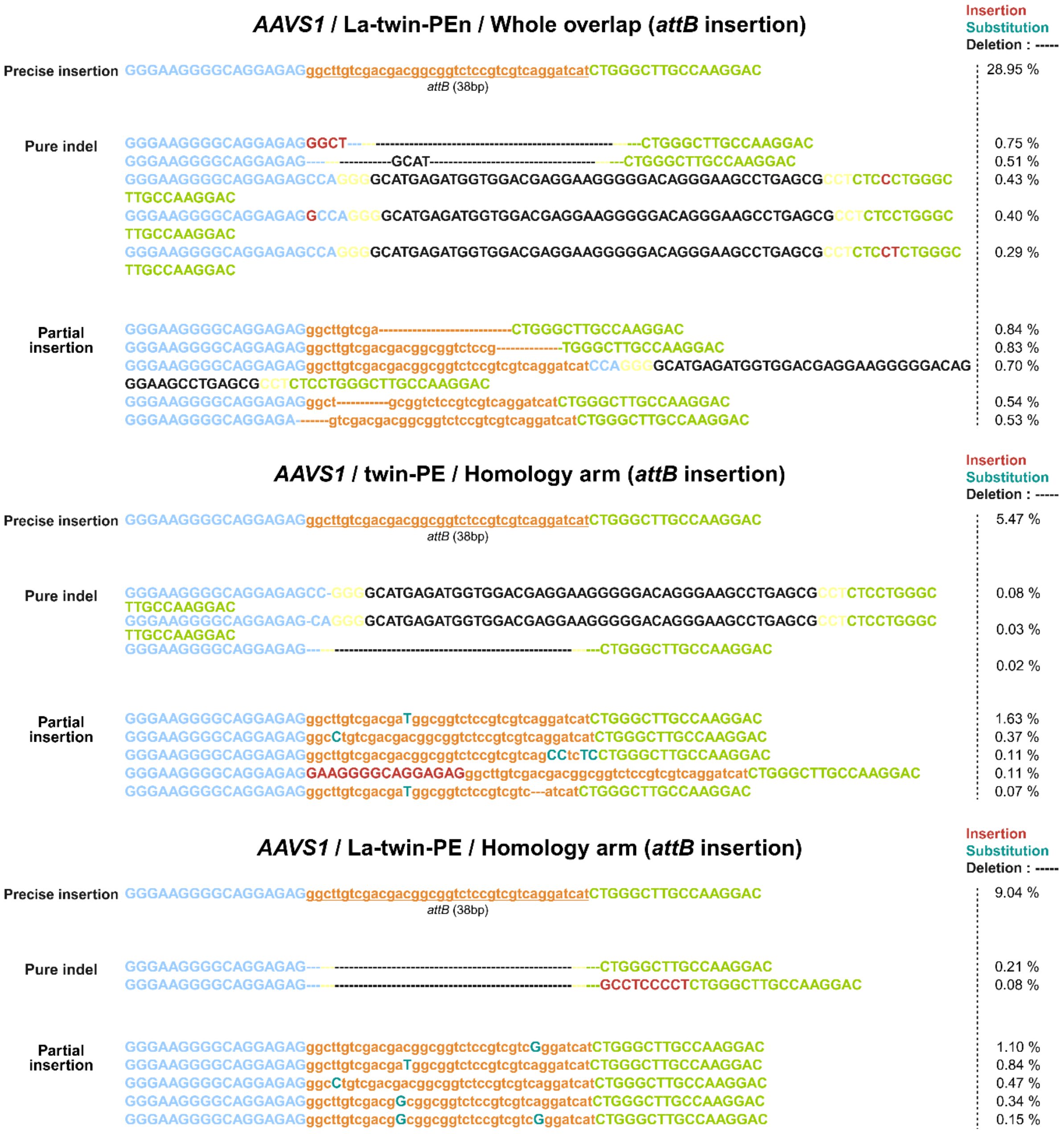
AAVS1 / La-twin-PEn / Whole overlap (attB insertion)
Insertion
Substitution
Deletion : -----
Precise insertion GGGAAGGGGCAGGAGAGggcttgtcgacgacggcggtctccgtcgtcaggatcatCTGGGCTTGCCAAGGAC 28.95 %
attB (38bp)
Pure indel
GGGAAGGGGCAGGAGAGGGCT---CTGGGCTTGCCAAGGAC 0.75 %
GGGAAGGGGCAGGAGAG--------------GCAT---CTGGGCTTGCCAAGGAC 0.51 %
GGGAAGGGGCAGGAGAGCCAGGGGCATGAGATGGTGGACGAGGAAGGGGGACAGGGAAGCCTGAGCGCCTCTCCCTGGGC TTGCCAAGGAC 0.43 %
GGGAAGGGGCAGGAGAGGCCAGGGGCATGAGATGGTGGACGAGGAAGGGGGACAGGGAAGCCTGAGCGCCTCTCCTGGGC TTGCCAAGGAC 0.40 %
GGGAAGGGGCAGGAGAGCCAGGGGCATGAGATGGTGGACGAGGAAGGGGGACAGGGAAGCCTGAGCGCCTCTCCTCTGGGC TTGCCAAGGAC 0.29 %
Partial insertion
GGGAAGGGGCAGGAGAGggcttgtcga----------------------------CTGGGCTTGCCAAGGAC 0.84 %
GGGAAGGGGCAGGAGAGggcttgtcgacgacggcggtctccg--------------TGGGCTTGCCAAGGAC 0.83 %
GGGAAGGGGCAGGAGAGggcttgtcgacgacggcggtctccgtcgtcaggatcatCCAGGGGCATGAGATGGTGGACGAGGAAGGGGGACAG GGAAGCCTGAGCGCCTCTCCTGGGCTTGCCAAGGAC 0.70 %
GGGAAGGGGCAGGAGAGggct-----------gcggtctccgtcgtcaggatcatCTGGGCTTGCCAAGGAC 0.54 %
GGGAAGGGGCAGGAGA-------gtcgacgacggcggtctccgtcgtcaggatcatCTGGGCTTGCCAAGGAC 0.53 %
AAVS1 / twin-PE / Homology arm (attB insertion)
Insertion
Substitution
Deletion : -----
Precise insertion GGGAAGGGGCAGGAGAGggcttgtcgacgacggcggtctccgtcgtcaggatcatCTGGGCTTGCCAAGGAC 5.47 %
attB (38bp)
Pure indel
GGGAAGGGGCAGGAGAGCC-GGGGCATGAGATGGTGGACGAGGAAGGGGGACAGGGAAGCCTGAGCGCCTCTCCTGGGC TTGCCAAGGAC 0.08 %
GGGAAGGGGCAGGAGAG-CAGGGGCATGAGATGGTGGACGAGGAAGGGGGACAGGGAAGCCTGAGCGCCTCTCCTGGGC TTGCCAAGGAC 0.03 %
GGGAAGGGGCAGGAGAG--CTGGGCTTGCCAAGGAC 0.02 %
Partial insertion
GGGAAGGGGCAGGAGAGggcttgtcgacgaTggcggtctccgtcgtcaggatcatCTGGGCTTGCCAAGGAC 1.63 %
GGGAAGGGGCAGGAGAGggcCtgtcgacgacggcggtctccgtcgtcaggatcatCTGGGCTTGCCAAGGAC 0.37 %
GGGAAGGGGCAGGAGAGggcttgtcgacgacggcggtctccgtcgtcagCCtcTCCTGGGCTTGCCAAGGAC 0.11 %
GGGAAGGGGCAGGAGAGGAAGGGGCAGGAGAGggcttgtcgacgacggcggtctccgtcgtcaggatcatCTGGGCTTGCCAAGGAC 0.11 %
GGGAAGGGGCAGGAGAGggcttgtcgacgaTggcggtctccgtcgtc---atcatCTGGGCTTGCCAAGGAC 0.07 %
AAVS1 / La-twin-PE / Homology arm (attB insertion)
Insertion
Substitution
Deletion : -----
Precise insertion GGGAAGGGGCAGGAGAGggcttgtcgacgacggcggtctccgtcgtcaggatcatCTGGGCTTGCCAAGGAC 9.04 %
attB (38bp)
Pure indel
GGGAAGGGGCAGGAGAG--CTGGGCTTGCCAAGGAC 0.21 %
GGGAAGGGGCAGGAGAG--GCCTCCCCTCTGGGCTTGCCAAGGAC 0.08 %
Partial insertion
GGGAAGGGGCAGGAGAGggcttgtcgacgacggcggtctccgtcgtcGggatcatCTGGGCTTGCCAAGGAC 1.10 %
GGGAAGGGGCAGGAGAGggcttgtcgacgaTggcggtctccgtcgtcaggatcatCTGGGCTTGCCAAGGAC 0.84 %
GGGAAGGGGCAGGAGAGggcCtgtcgacgacggcggtctccgtcgtcaggatcatCTGGGCTTGCCAAGGAC 0.47 %
GGGAAGGGGCAGGAGAGggcttgtcgacgGcggcggtctccgtcgtcaggatcatCTGGGCTTGCCAAGGAC 0.34 %
GGGAAGGGGCAGGAGAGggcttgtcgacgGcggcggtctccgtcgtcGggatcatCTGGGCTTGCCAAGGAC 0.15 %

AAVS1 / twin-PEn / Homology arm (*attB* insertion)

Insertion
Substitution
Deletion : -----

Precise insertion	GGGAAGGGGCAGGAGAGggcttgtcgacgacggcggtctccgtcgtcaggatcatCTGGGCTTGCCAAGGAC<br>*attB* (38bp)	5.18 %
Pure indel	GGGAAGGGGCAGGAGAG--CTGGGCTTGCCAAGGAC	7.56 %
	GGGAAGGGGCAGGAGAGCCAGGGGCATGAGATGGTGGACGAGGAAGGGGGACAGGGAAGCCTGAGCGCCTCTCCCTGGGCTTGCCAAGGAC	1.45 %
	GGGAAGGGGCAGGAGAGGCCAGGGGCATGAGATGGTGGACGAGGAAGGGGGACAGGGAAGCCTGAGCGCCTCTCCTGGGCTTGCCAAGGAC	1.04 %
	GGGAAGGGGCAGGAGA---CTGGGCTTGCCAAGGAC	0.55 %
	GGGAAGGGGCAGGAGAGAGCCAGGGGCATGAGATGGTGGACGAGGAAGGGGGACAGGGAAGCCTGAGCGCCTCTCCTGGGCTTGCCAAGGAC	0.52 %
Partial insertion	GGGAAGGGGCAGGAGAG-------------ggcggtctccgtcgtcaggatcatCTGGGCTTGCCAAGGAC	0.47 %
	GGGAAGGGGCAGGAGAGGAGggcttgtcgacgacggcggtctccgtcgtcaggatcatCTGGGCTTGCCAAGGAC	0.34 %
	GGGAAGGGGCAGGAGAGggcttgtcgacgacggcggtctccgtcgtca-------CTGGGCTTGCCAAGGAC	0.31 %
	GGGAAGGGGCAGGAGAGggctt---------------------------------CTGGGCTTGCCAAGGAC	0.30 %
	GGGAAGGGGCAGGAGAGggcttgtcgacgaTggcggtctccgtcgtcaggatcatCTGGGCTTGCCAAGGAC	0.29 %

AAVS1 / La-twin-PEn / Homology arm (*attB* insertion)

Insertion
Substitution
Deletion : -----

Precise insertion	GGGAAGGGGCAGGAGAGggcttgtcgacgacggcggtctccgtcgtcaggatcatCTGGGCTTGCCAAGGAC<br>*attB* (38bp)	4.14 %
Pure indel	GGGAAGGGGCAGGAGAG--CTGGGCTTGCCAAGGAC	18.25 %
	GGGAAGGGGCAGGAGAGGCCAGGGGCATGAGATGGTGGACGAGGAAGGGGGACAGGGAAGCCTGAGCGCCTCTCCTGGGCTTGCCAAGGAC	1.17 %
	GGGAAGGGGCAGGAGAGCCAGGGGCATGAGATGGTGGACGAGGAAGGGGGACAGGGAAGCCTGAGCGCCTCTCCCTGGGCTTGCCAAGGAC	0.86 %
	GGGAAGGGGCAGGAGAGAT--CTGGGCTTGCCAAGGAC	0.77 %
	GGGAAGGGGCAGGAGAGCCAG--CTGGGCTTGCCAAGGAC	0.63 %
Partial insertion	GGGAAGGGGCAGGAGAGggcttgtcg----------------------------CTGGGCTTGCCAAGGAC	0.82 %
	GGGAAGGGGCAGGAGAGggcttgtcgacgacggcggtctccgtcgtcag------CTGGGCTTGCCAAGGAC	0.42 %
	GGGAAGGGGCAGGAGAGggcttgtcgacgaTggcggtctccgtcgtcaggatcatCTGGGCTTGCCAAGGAC	0.24 %
	GGGAAGGGGCAGGAGAGGAAGGGGCAGGAGAGggcttgtcgacgacggcggtctccgtcgtcaggatcatCTGGGCTTGCCAAGGAC	0.18 %
	GGGAAGGGGCAGGAGAGAAGGGGCAGGAGAGggcttgtcgacgacggcggtctccgtcgtcaggatcatCTGGGCTTGCCAAGGAC	0.17 %

AAVS1 / twin-PE / Partial overlap (*attB* insertion)

Insertion
Substitution
Deletion : -----

Precise insertion	GGGAAGGGGCAGGAGAGggcttgtcgacgacggcggtctccgtcgtcaggatcatCTGGGCTTGCCAAGGAC<br>*attB* (38bp)	12.66 %
Pure indel	GGGAAGGGGCAGGAGAG--CTGGGCTTGCCAAGGAC	0.08 %
	GGGAAGGGGCAGGAGAGGGCTCCAGGGGCATGAGATGGTGGACGAGGAAGGGGGACAGGGAAGCCTGAGCGCCTCTCCTGGGCTTGCCAAGGAC	0.08 %
Partial insertion	GGGAAGGGGCAGGAGAGggcttgtcgacgacggcggtctc---cgtcaggatcatCTGGGCTTGCCAAGGAC	0.24 %
	GGGAAGGGGCAGGAGAGggcttgtcgacga------------tcgtcaggatcatCTGGGCTTGCCAAGGAC	0.22 %
	GGGAAGGGGCAGGAGAGggcttgt---cgacggcggtctccgtcgtcaggatcatCTGGGCTTGCCAAGGAC	0.19 %
	GGGAAGGGGCAGGAGAG-------------ggcggtctccgtcgtcaggatcatCTGGGCTTGCCAAGGAC	0.17 %
	GGGAAGGGGCAGGAGAGggcttgGcgacgacggcggtctccgtcgtcaggatcatCTGGGCTTGCCAAGGAC	0.16 %

AAVS1 / La-twin-PE / Partial overlap (*attB* insertion)

Insertion
Substitution
Deletion : -----

Precise insertion	GGGAAGGGGCAGGAGAGggcttgtcgacgacggcggtctccgtcgtcaggatcatCTGGGCTTGCCAAGGAC<br>*attB* (38bp)	23.77 %
Pure indel	None	
Partial insertion	GGGAAGGGGCAGGAGAGggcttgtcgacgacggcggtctc---cgtcaggatcatCTGGGCTTGCCAAGGAC	1.09 %
	GGGAAGGGGCAGGAGAGggcttgtcgacgacggcgGTCTCCgtctccgtcgtcaggatcatCTGGGCTTGCCAAGGAC	1.02 %
	GGGAAGGGGCAGGAGAGggcttgtcgacgacggcggtc--------------CCAGGGGCATGAGATGGTGGACGAGGAAGGGGGACAGGGAAGCCTGAGCGCCTCTCCTGGGCTTGCCAAGGAC	0.50 %
	GGGAAGGGGCAGGAGAGggcttgtcgacgacggcggtctccgtcgtGca-----------------------gtctccgtcgtcaggatcatCTGGGCTTGCCAAGGAC<br>Sense insertion / Anti-Sense insertion	0.30 %
	GGGAAGGGGCAGGAGAGggcttgGcgacgacggcggtctccgtcgtcaggatcatCTGGGCTTGCCAAGGAC	0.25 %

AAVS1 / twin-PEn / Partial overlap (*attB* insertion)

Insertion
Substitution
Deletion : -----

Precise insertion	GGGAAGGGGCAGGAGAGggcttgtcgacgacggcggtctccgtcgtcaggatcatCTGGGCTTGCCAAGGAC<br>*attB* (38bp)	8.21 %
Pure indel	GGGAAGGGGCAGGAGAG--CTGGGCTTGCCAAGGAC	0.79 %
	GGGAAGGGGCAGGAGAGCCAGGGGCATGAGATGGTGGACGAGGAAGGGGACAGGGAAGCCTGAGCGCCTCTCCCTGGGCTTGCCAAGGAC	0.49 %
	GGGAAGGGGCAGGAGAGCCAGGGGCATGAGATGGTGGACGAGGAAGGGGACAGGGAAGCCTGAGCGCCTCTCCCATCTGGGCTTGCCAAGGAC	0.30 %
	GGGAAGGGGCAGGAGAGCCAGGGGCATGAGATGGTGGACGAGGAAGGGGACAGGGAAGCCTGAGCGCCTCTCATCTGGGCTTGCCAAGGAC	0.19 %
	GGGAAGGGGCAGGAGAGCCAGGGGCATGAGATGGTGGACGAGGAAGGGGACAGGGAAGCCTGAGCGCCTCTCCTCTGGGCTTGCCAAGGAC	0.17 %
Partial insertion	GGGAAGGGGCAGGAGAGggcttgtcgacgacggcggtctccgtcgt---------CCAGGGGCATGAGATGGTGGACGAGGAAGGGGACAGGGAAGCCTGAGCGCCTCTCCTGGGCTTGCCAAGGAC	1.22 %
	GGGAAGGGGCAGGAGAG-------------------tctccgtcgtcaggatcatCTGGGCTTGCCAAGGAC	0.84 %
	GGGAAGGGGCAGGAGAGggcttgtcgacgacggcggt-----------------CCAGGGGCATGAGATGGTGGACGAGGAAGGGGACAGGGAAGCCTGAGCGCCTCTCCTGGGCTTGCCAAGGAC	0.79 %
	GGGAAGGGGCAGGAGAGCCAGGGGCATGAGATGGTGGACGAGGAAGGGGACAGGGAAGCCTGAGCGCCTCTC--------CacgacggcggtctccgtcgtcaggatcatCTGGGCTTGCCAAGGAC	0.51 %
	GGGAAGGGGCAGGAGAGggcttgtcgacgacggcggtctc--------------CCAGGGGCATGAGATGGTGGACGAGGAAGGGGACAGGGAAGCCTGAGCGCCTCTCCTGGGCTTGCCAAGGAC	0.49 %

AAVS1 / La-twin-PEn / Partial overlap (*attB* insertion)

Insertion
Substitution
Deletion : -----

Precise insertion	GGGAAGGGGCAGGAGAGggcttgtcgacgacggcggtctccgtcgtcaggatcatCTGGGCTTGCCAAGGAC<br>*attB* (38bp)	7.32 %
Pure indel	GGGAAGGGGCAGGAGAGGAT---CTGGGCTTGCCAAGGAC	0.98 %
	GGGAAGGGGCAGGAGAG---CTGGGCTTGCCAAGGAC	0.94 %
	GGGAAGGGGCAGGAGAG--TCATCTGGGCTTGCCAAGGAC	0.72 %
	GGGAAGGGGCAGGAGAG---------------------GAGGA-------------------TCATCTGGGCTTGCCAAGGAC	0.69 %
Partial insertion	GGGAAGGGGCAGGAGAGggcttgtcgacgacggcggtctccgtcgt---------CCAGGGGCATGAGATGGTGGACGAGGAAGGGGACAGGGAAGCCTGAGCGCCTCTCCTGGGCTTGCCAAGGAC	0.86 %
	GGGAAGGGGCAGGAGAGggcttgtcgacgacggcggtctccgtcg---------tGCTGCAGAAGGGATTCC	0.61 %
	GGGAAGGGGCAGGAGAGggcttgtcgacgacggcggt---cgtcaggatcatGCTGCAGAAGGGATTCC	0.53 %
	GGGAAGGGGCAGGAGAG--------------------------Ccgtcaggatcat GCTGCAGAAGGGATTCC	0.41 %
	GGGAAGGGGCAGGAGAGggcttgt---------cggtctccgtcgtcaggatcatCTGGGCTTGCCAAGGAC	0.39 %

AAVS1 Twin-prime editing pattern (*attP* insertion)

AAVS1

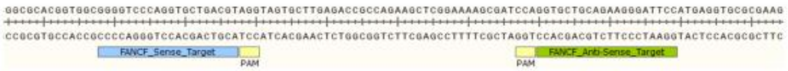

Insertion
Subtitution
Deletion : -----

Normal control	GGGAAGGGGCAGGAGAGCCAGGGGCATGAGATGGTGGACGAGGAAGGGGACAGGGAAGCCTGAGCGCCTCTCCTGGGCTTGCCAAGGAC

AAVS1 / twin-PE / Whole overlap (*attP* insertion)

Insertion
Substitution
Deletion : -----

Precise insertion	GGGAAGGGGCAGGAGAGaggtttgtctggtcaaccaccgcggtctcagtggtgtacggtacaaacctCTGGGCTTGCCAAGGAC<br>*attP* (50bp)	13.41 %
Pure indel	GGGAAGGGGCAGGAGAG-------------------------------GAA-------------------------------CTGGGCTTGCCAAGGAC	0.55 %
	GGGA---------------AGGGGCATGAGATGGTGGACGAGGAAGGGGACAGGGAAGCCTGAGCGCCTCTCCTGGGCTTGCCAAGGAC	0.10 %
Partial insertion	GGGAAGGGGCAGGAGAGCggtttgtctggtcaaccaccgcggtctcagtggtgtacggtacaaacctCTGGGCTTGCCAAGGAC	0.30 %
	GGGAAGGGGCAGGAGAG--------------TccaccgcggtctcagtggtgtacggtacaaacctCTGGGCTTGCCAAGGAC	0.17 %
	GGGAAGGGGCAGGAGAGaggtttgtctggtcGaccaccgcggtctcagtggtgtacggtacaaacctCTGGGCTTGCCAAGGAC	0.15 %
	GGGAAGGGGCAGGAGTGaggtttgtctggtcaaccaccgcggtctcagtggtgtacggtacaaacctCTGGGCTTGCCAAGGAC	0.10 %
	GGGAAGGGGCAGGAGAGaggtttgtctggtcaaccCccgcggtctcagtggtgtacggtacaaacctCTGGGCTTGCCAAGGAC	0.08 %

AAVS1 / La-twin-PE / Whole overlap (*attP* insertion)

Insertion
Substitution
Deletion : -----

	Sequence	%
Precise insertion	GGGAAGGGGCAGGAGAGaggtttgtctggtcaaccaccgcggtctcagtggtgtacggtacaaacctCTGGGCTTGCCAAGGAC (*attP* (50bp))	20.72 %
Pure indel	None	
Partial insertion	GGGAAGGGGCAGGAGTGaggtttgtctggtcaaccaccgcggtctcagtggtgtacggtacaaacctCTGGGCTTGCCAAGGAC	0.12 %
	GGGAAGGGGCAGGAGAGaggtttgtctggtcaaccaccgcggtctcagtggtgtacggtac---CAGGGAAGCCTGAGCGCCTCTCCTGGGCTTGCCAAGGAC	0.12 %
	GGGAAGGGGCAGGAGAGaggtttgtctggtcaaccaccgcggtctcagtggtgt------------------------------ACGAGGAAGGGGGACAGGGAAGCCTGAGCGCCTCTCCTGGGCTTGCCAAGGAC	0.09 %
	GGGAAGGGGCAGGAGAGaggtttgtctggtcaaccCccgcggtctcagtggtgtacggtacaaacctCTGGGCTTGCCAAGGAC	0.07 %
	GGGAAGGGGCAGGAGAGaggtttgtctggtcaaccaccgcggtctcagtggtgtacggtacaaCcctCTGGGCTTGCCAAGGAC	0.07 %

AAVS1 / twin-PEn / Whole overlap (*attP* insertion)

Insertion
Substitution
Deletion : -----

	Sequence	%
Precise insertion	GGGAAGGGGCAGGAGAGaggtttgtctggtcaaccaccgcggtctcagtggtgtacggtacaaacctCTGGGCTTGCCAAGGAC (*attP* (50bp))	9.55 %
Pure indel	GGGAAGGGGCAGGAGAGCCAGGGGCATGAGATGGTGGACGAGGAAGGGGGACAGGGAAGCCTGAGCGCCTCTCCTCTGGGCTTGCCAAGGAC	2.45 %
	GGGAAGGGGCAGGAGAG---CTGGGCTTGCCAAGGAC	1.23 %
	GGGAAGGGGCAGGAGAGCCAGGGGCATGAGATGGTGGACGAGGAAGGGGGACAGGGAAGCCTGAGCGCCTCTCTCTGGGCTTGCCAAGGAC	0.75 %
	GGGAAGGGGCAGGAGAGCCAGGGGCATGAGATGGTGGACGAGGAAGGGGGACAGGGAAGCCTGAGCGCCTCTCCTTCTGGGCTTGCCAAGGAC	0.41 %
	GGGAAGGGGCAGGAGAGGAGGCCAGGGGCATGAGATGGTGGACGAGGAAGGGGGACAGGGAAGCCTGAGCGCCTCTCCTGGGCTTGCCAAGGAC	0.29 %
Partial insertion	GGGAAGGGGCAGGAGAGaggtttgtctggtcaaccaccgcggtctcagt------------------CTGGGCTTGCCAAGGAC	0.76 %
	GGGAAGGGGCAGGAGAGaggtttgtctggtcaacca-------------------------------CTGGGCTTGCCAAGGAC	0.67 %
	GGGAAGGGGCAGGAGAGaggtttgtctggtca-----------------------------------CTGGGCTTGCCAAGGAC	0.59 %
	GGGAAGGGGCAGGAGAGaggtttgtctggt-------------------------------------CTGGGCTTGCCAAGGAC	0.48 %
	GGGAAGGGGCAGGAGAGaggtttgtctggtcaaccaccgcggtctcagtggtgtacg---gtacggtacaaacctCTGGGCTTGCCAAGGAC (Sense insertion / Anti-Sense insertion)	0.43 %

AAVS1 / La-twin-PEn / Whole overlap (*attP* insertion)

Insertion
Substitution
Deletion : -----

	Sequence	%
Precise insertion	GGGAAGGGGCAGGAGAGaggtttgtctggtcaaccaccgcggtctcagtggtgtacggtacaaacctCTGGGCTTGCCAAGGAC (*attP* (50bp))	5.77 %
Pure indel	GGGAAGGGGCAGGAGAGCCAGGGGCATGAGATGGTGGACGAGGAAGGGGGACAGGGAAGCCTGAGCGCCTCTCCTCTGGGCTTGCCAAGGAC	2.38 %
	GGGAAGGGGCAGGAGAG---CTCTGGGCTTGCCAAGGAC	0.93 %
	GGGAAGGGGCAGGAGAG---CTGGGCTTGCCAAGGAC	0.45 %
	GGGAAGGGGCAGGAGAG--GCCAC--CTGGGCTTGCCAAGGAC	0.29 %
	GGGAAGGGGCAGGAGAG---CCTGGGCTTGCCAAGGAC	0.28 %
Partial insertion	GGGAAGGGGCAGGAGAGaggtttg---CTGGGCTTGCCAAGGAC	0.56 %
	GGGAAGGGGCAGGAGAGaggtttgtctgT--------------------------------------CTGGGCTTGCCAAGGAC	0.33 %
	GGGAAGGGGCAGGAGAGaggtttgtctg---------------------------------------CTGGGCTTGCCAAGGAC	0.29 %
	GGGAAGGGGCAGGAGAGaggtttgtctggtcaaccaccgcggtctcagtggtgtacggtacaa----CTGGGCTTGCCAAGGAC	0.29 %
	GGGAAGGGGCAGGAGAGaggtttgtctggtcaaccaccgcggtctcagtggtgtacggtacaaacctCCAGGGGCATGAGATGGTGGACGAGGAAGGGGGACAGGGAAGCCTGAGCGCCTCTCCTGGGCTTGCCAAGGAC	0.28 %

AAVS1 / twin-PE / Homology arm (*attP* insertion)

Insertion
Substitution
Deletion : -----

Precise insertion	GGGAAGGGGCAGGAGAGaggtttgtctggtcaaccaccgcggtctcagtggtgtacggtacaaacctCTGGGCTTGCCAAGGAC<br>*attP* (50bp)	1.93 %
Pure indel	GGGAAGGGGCAGGAGAG---CTGGGCTTGCCAAGGAC	0.04 %
	GGGAAGGGGCAGGAGA-CCAGGGGCATGAGATGGTGGACGAGGAAGGGGGACAGGGAAGCCTGAGCGCCTCTCCTGGGCTTGCCAAGGAC	0.02 %
Partial insertion	GGGAAGGGGCAGGAGAGaggtttgtctggtcaaccaccgcggtctcag---------------------------------TGGTGGACGAGGAAGGGGGACAGGGAAGCCTGAGCGCCTCTCCTGGGCTTGCCAAGGAC	0.20 %
	GGGAAGGGGCAGGAGAGaggtttgtctggtcaaccaccgcggtctcagtggtgtacggtacaaacctCTGGGCTTGCCAAGG---------------------------------------GCCTGAGCGCCTCTCCTGGGCTTGCCAAGGAC	0.06 %
	GGGAAGGGGCAGGAGAGaggtttgtctggtcaaccaccgcggCctcagtggtgCacggtacaaacct----------------------------------GGCA-CC-GAGCGCCTGAGCGCCTCTCCTGGGCTTGCCAAGGAC	0.06 %
	GGGAAGGGGCAGGAGAGaggtttgtcCggtcaaccaccgcggtctcagtggtgtacggtacaaacctCTGGGCTTGCCAAGGAC	0.04 %
	GGGAAGGGGCAGGAGAGaggtttgtctggtcaaccaccgcggtctcagtggtgCacggtacaaacctCTGGGCTTGCCAAGGAC	0.04 %

AAVS1 / La-twin-PE / Homology arm (*attP* insertion)

Insertion
Substitution
Deletion : -----

Precise insertion	GGGAAGGGGCAGGAGAGaggtttgtctggtcaaccaccgcggtctcagtggtgtacggtacaaacctCTGGGCTTGCCAAGGAC<br>*attP* (50bp)	0.76 %
Pure indel	GGGAAGGGGCAGGAGAG---CTGGGCTTGCCAAGGAC	0.26 %
	GGGAAGGGGCAG--------------GAGATGGTGGACGAGGAAGGGGGACAGGGAAGCCTGAGCGCCTCTCCTGGGCTTGCCAAGGAC	0.22 %
	GGGAAGGGGCAGGA---GGAAGCCTGAGCGCCTCTCCTGGGCTTGCCAAGGAC	0.11 %
	GGGA--------------AGGGGCATGAGATGGTGGACGAGGAAGGGGGACAGGGAAGCCTGAGCGCCTCTCCTGGGCTTGCCAAGGAC	0.03 %
	GGGAAGGGGCAGGAGAG-CAGGGGCATGAGATGGTGGACGAGGAAGGGGGACAGGGAAGCCTGAGCGCCTCTCCTGGGCTTGCCAAGGAC	0.02 %
Partial insertion	GGGAAGGGGCAGGAGAGaggtttgtctggtcaaccacc--GCCTGAGCGCCTCTCCTGGGCTTGCCAAGGAC	0.12 %
	GGGAAGGGGCAGGAGAGaggtttgtcCggtcaaccaccgcggtctcagtggtgCacggCacaaacctCTGGGCTTGCCAAGGAC	0.10 %
	GGGAAGGGGCAGGAGAGaggtttgtcCggtcaaccaccgcggtctcagtggtgtacggtacaaacctCTGGGCTTGCCAAGGAC	0.08 %
	GGGAAGGGGCAGGAGAGaggtttgtctggtcaaccaccgcggtctcagtggtgtacggtGcaaacctCTGGGCTTGCCAAGGAC	0.07 %
	GGGAAGGGGCAGGAGAGaggCttgtctggtcaaccaccgcggtcCcagtggCgtacggCacaaacctCTGGGCTTGCCAAGGAC	0.07 %

AAVS1 / twin-PEn / Homology arm (*attP* insertion)

Insertion
Substitution
Deletion : -----

Precise insertion	GGGAAGGGGCAGGAGAGaggtttgtctggtcaaccaccgcggtctcagtggtgtacggtacaaacctCTGGGCTTGCCAAGGAC<br>*attP* (50bp)	0.78 %
Pure indel	GGGAAGGGGCAGGAGAG---CTGGGCTTGCCAAGGAC	19.14 %
	GGGAAGGGGCAGGAGAGCCAGGGGCATGAGATGGTGGACGAGGAAGGGGGACAGGGAAGCCTGAGCGCCTCTCCCTGGGCTTGCCAAGGAC	2.15 %
	GGGAAGGGGCAGGAGAGGCCAGGGGCATGAGATGGTGGACGAGGAAGGGGGACAGGGAAGCCTGAGCGCCTCTCCTGGGCTTGCCAAGGAC	1.10 %
	GGGAAGGGGCAGGAGAGCCAGGGGCATGAGATGGTGGACGAGGAAGGGGGACAGGGAAGCCTGAGCGCCTCTCCTCTGGGCTTGCCAAGGAC	0.85 %
	GGGAAGGGGCAGGAGAG---ACTGGGCTTGCCAAGGAC	0.68 %
Partial insertion	GGGAAGGGGCAGGAGAGaggttt---CCAGGGGCATGAGATGGTGGACGAGGAAGGGGGACAGGGAAGCCTGAGCGCCTCTCCTGGGCTTGCCAAGGAC	0.16 %
	GGGAAGGGGCAGGAGAGaggtttgtctggtcaaccaccgc--------------------------CCAGGGGCATGAGATGGTGGACGAGGAAGGGGGACAGGAAGCCTGAGCGCCTCTCCTGGGCTTGCCAAGGAC	0.15 %
	GGGAAGGGGCAGGAGAGaggtttgtctggtcaaccaccgcggtctcagtggtgtacggtacaaacctCTGGGCTTGCTGGGCTTGCCAAGGAC	0.08 %
	GGGAAGGGGCAGGAGAGaggtttgtctggtcaaccaccgcggtctcagtggtgtacggtacaaacctCTGCTGGGCTTGCCAAGGAC	0.03 %
	GGGAAGGGGCAGGAGAaggtttgtctggtcaaccaccgcggtctcagtggtgtacggtacaaacctCTGGGCTTGCCAGGGGCATGAGATGGTGGACGAGGAAGGGGGACAGGGAAGCCTGAGCGCCTCTCCTGGGCTTGCCAAGGAC	0.03 %

Insertion
Substitution
Deletion : -----

AAVS1 / La-twin-PEn / Homology arm (*attP* insertion)

	Sequence	%
Precise insertion	GGGAAGGGGCAGGAGAGaggtttgtctggtcaaccaccgcggtctcagtggtgtacggtacaaacctCTGGGCTTGCCAAGGAC<br>*attP* (50bp)	0.61 %
Pure indel	GGGAAGGGGCAGGAGAG---CTGGGCTTGCCAAGGAC	12.89 %
	GGGAAGGGGCAGGAGAGCCAGGGGCATGAGATGGTGGACGAGGAAGGGGGACAGGGAAGCCTGAGCGCCTCTCCCTGGGCTTGCCAAGGAC	2.05 %
	GGGAAGGGGCAGGAGAGCCAGGGGCATGAGATGGTGGACGAGGAAGGGGGACAGGGAAGCCTGAGCGCCTCTCCTCTGGGCTTGCCAAGGAC	0.74 %
	GGGAAGGGGCAGGAGAGGCCAGGGGCATGAGATGGTGGACGAGGAAGGGGGACAGGGAAGCCTGAGCGCCTCTCCTGGGCTTGCCAAGGAC	0.68 %
	GGGAAGGGGCAGGAGA--TCTGGGCTTGCCAAGGAC	0.61 %
Partial insertion	GGGAAGGGGCAGGAGAGaggtttgtctggtcaaccaccgcggtctcagtggtgtacggtaca-----CTGGGCTTGCCAAGGAC	0.13 %
	GGGAAGGGGCAGGAGAGaggtttgtctggtcaaccaccgc--------------------------CCAGGGGCATGAGATGGTGGACGAGGAAGGGGGACAGGGAAGCCTGAGCGCCTCTCCTGGGCTTGCCAAGGAC	0.05 %
	GGGAAGGGGCAGGAGAaggtttgtctggtcaaccaccgcggtctcagtggtgtacggtacaaacctCTGGGCTTGCCAAGGGCCAGGGGCATGAGATGGTGGACGAGGAAGGGGGACAGGGAAGCCTGAGCGCCTCTCCTGGGCTTGCCAAGGAC	0.04 %
	GGGAAGGGGCAGGAGAGaggtttgtctggtcaaccaccgcggtctcagtggtgtacggtacaaacctCTCTGGGCTTGCCAAGGAC	0.04 %
	GGGAAGGGGCAGGAGAGaggtttgtctggtcaaccaccgcggtctc--------------------CTGGGCTTGCCAAGGAC	0.04 %

Insertion
Substitution
Deletion : -----

AAVS1 / twin-PE / Partial overlap (*attP* insertion)

	Sequence	%
Precise insertion	GGGAAGGGGCAGGAGAGaggtttgtctggtcaaccaccgcggtctcagtggtgtacggtacaaacctCTGGGCTTGCCAAGGAC<br>*attP* (50bp)	6.39 %
Pure indel	GGGA--------------AGGGGCATGAGATGGTGGACGAGGAAGGGGGACAGGGAAGCCTGAGCGCCTCTCCTGGGCTTGCCAAGGAC	0.08 %
	GGGAAGGGGCAGGAGAG-CAGGGGCATGAGATGGTGGACGAGGAAGGGGGACAGGGAAGCCTGAGCGCCTCTCCTGGGCTTGCCAAGGAC	0.02 %
Partial insertion	GGGAAGGGGCAGGAGAG------------AtcaaccaccgcggtctcagtggtgtacggtacaaacctCTGGGCTTGCCAAGGAC	0.10 %
	GGGAAGGGGCAGGAGAGaggtttgtctggt-------cgcggtctcagtggtgtacggtacaaacctCTGGGCTTGCCAAGGAC	0.09 %
	GGGAAGGGGCAGGAGAGaggtttgtctggtcaaccaccgc-------------------------------------gtcaaccaccgcggtctcagtggtgtacggtacaaacctCTGGGCTTGCCAAGGAC<br>Sense insertion / Anti-Sense insertion	0.06 %
	GGGAAGGGGCAGGAGAGCCAGGGGCATGAGATGGTGGACGAGGAAGGGGGACAGGGAAGC-----------------------gtcaaccaccgcggtctcagtggtgtacggtacaaacctCTGGGCTTGCCAAGGAC	0.05 %
	GGGAAGGGGCAGGAGAGaggtttgtctggtcaaccaccgcggtctcagtggtgtacggtacaaacctCTGGGCT----------------------------------GAAGCCTGAGCGCCTCTCCTGGGCTTGCCAAGGAC	0.04 %

Insertion
Substitution
Deletion : -----

AAVS1 / La-twin-PE / Partial overlap (*attP* insertion)

	Sequence	%
Precise insertion	GGGAAGGGGCAGGAGAGaggtttgtctggtcaaccaccgcggtctcagtggtgtacggtacaaacctCTGGGCTTGCCAAGGAC<br>*attP* (50bp)	11.79 %
Pure indel	None	
Partial insertion	GGGAAGGGGCAGGAGAG--------ctgCgtcaaccaccgcggtctcagtggtgtacggtacaaacctCTGGGCTTGCCAAGGAC	0.18 %
	GGGAAGGGGCAGGAGAGaggtttgtctggtcaaccaccgc---------ggtgtacggtacaaacctCTGGGCTTGCCAAGGAC	0.11 %
	GGGAAGGGGCAGGAGAGaggtttgtctCgtcaaccaccgcggtctcagtggtgtacggtacaaacctCTGGGCTTGCCAAGGAC	0.08 %
	GGGAAGGGGCAGGAGAGaggtttgtctggtcaaccaccgcggtctcagCggt-----------------------------------gcggtctcagtggtgtacggtacaaacctCTGGGCTTGCCAAGGAC<br>Sense insertion / Anti-Sense insertion	0.07 %
	GGGAAGGGGCAGGAGAGaggtttgtctggtcaaccaccgcggtctcagtggt---gtggtgtacggtacaaacctCTGGGCTTGCCAAGGAC<br>Sense insertion / Anti-Sense insertion	0.07 %

AAVS1 / twin-PEn / Partial overlap (*attP* insertion)

Insertion
Substitution
Deletion : -----

	Sequence	%
Precise insertion	GGGAAGGGGCAGGAGAGaggtttgtctggtcaaccaccgcggtctcagtggtgtacggtacaaacctCTGGGCTTGCCAAGGAC<br>*attP* (50bp)	3.74 %
Pure indel	GGGAAGGGGCAGGAGAG---CTGGGCTTGCCAAGGAC	2.90 %
	GGGAAGGGGCAGGAGAGGCCAGGGGCATGAGATGGTGGACGAGGAAGGGGGACAGGGAAGCCTGAGCGCCTCTCCTGGGCTTGCCAAGGAC	1.71 %
	GGGAAGGGGCAGGAGAGCCAGGGGCATGAGATGGTGGACGAGGAAGGGGGACAGGGAAGCCTGAGCGCCTCTCCCTGGGCTTGCCAAGGAC	0.35 %
	GGGAAGGGGCAG-------------GAGATGGTGGACGAGGAAGGGGGACAGGGAAGCCTGAGCGCCTCTCCTGGGCTTGCCAAGGAC	0.27 %
	GGGAAGGGGCAGGAGAGCCAGGGGCATGAGATGGTGGACGAGGAAGGGGGACAGGGAAGCCTGAGCGCCTCTCCTCTGGGCTTGCCAAGGAC	0.21 %
Partial insertion	GGGAAGGGGCAGGAGAG----------CgtcaaccaccgcggtctcagtggtgtacggtacaaacctCTGGGCTTGCCAAGGAC	0.35 %
	GGGAAGGGGCAGGAGAGaggtttgtctggtcaaccaccgcggtctcagtggtgtacg----------CTGGGCTTGCCAAGGAC	0.33 %
	GGGAAGGGGCAGGAGAGaggtttgtctggtcaaccaccgcggtctcagtg-----------------CTGGGCTTGCCAAGGAC	0.30 %
	GGGAAGGGGCAGGAGAGaggtttgtctggtcaaccaccgcg--------------------------CTGGGCTTGCCAAGGAC	0.30 %
	GGGAAGGGGCAGGAGAG--------c--gtcaaccaccgcggtctcagtggtgtacggtacaaacctCTGGGCTTGCCAAGGAC	0.20 %

AAVS1 / La-twin-PEn / Partial overlap (*attP* insertion)

Insertion
Substitution
Deletion : -----

	Sequence	%
Precise insertion	GGGAAGGGGCAGGAGAGaggtttgtctggtcaaccaccgcggtctcagtggtgtacggtacaaacctCTGGGCTTGCCAAGGAC<br>*attP* (50bp)	5.12 %
Pure indel	GGGAAGGGGCAGGAGAG---CTGGGCTTGCCAAGGAC	1.51 %
	GGGAAGGGGCAGGAGAGAG---CTGGGCTTGCCAAGGAC	0.74 %
	GGGAAGGGGCAGGAGAGCCAGGGGCATGAGATGGTGGACGAGGAAGGGGGACAGGGAAGCCTGAGCGCCTCTCTCTGGGCTTGCCAAGGAC	0.23 %
	GGGAAGGGGCAGGAGAGCCAGGGGCATGAGATGGTGGACGAGGAAGGGGGACAGGGAAGCCTGAGCGCCTCTCCTCTGGGCTTGCCAAGGAC	0.13 %
	GGGAAGGGGCAGGAGAGAGGTCCAGGGGCATGAGATGGTGGACGAGGAAGGGGGACAGGGAAGCCTGAGCGCCTCTCCTGGGCTTGCCAAGGAC	0.03 %
Partial insertion	GGGAAGGGGCAGGAGAGaggtttgtctgg-------------------------------------CTGGGCTTGCCAAGGAC	0.27 %
	GGGAAGGGGCAGGAGAGaggtttgtctggtcaa---------------------------------CCTGGGCTTGCCAAGGAC	0.26 %
	GGGAAGGGGCAGGAGAGaggtttgtctggtcaa---------------------------------CTGGGCTTGCCAAGGAC	0.20 %
	GGGAAGGGGCAGGAGAGaggtttgtctggtcaaccaccgc------------------------------CCAGGGGCATGAGATGGTGGACGAGGAAGGGGGACAGGGAAGCCTGAGCGCCTCTCCTGGGCTTGCCAAGGAC	0.20 %
	GGGAAGGGGCAGGAGAGaggtttgtctggtcaaccaccgcggtctcagtggtgtac-----------CCAGGGGCATGAGATGGTGGACGAGGAAGGGGGACAGGGAAGCCTGAGCGCCTCTCCTGGGCTTGCCAAGGAC	0.17 %

HEK3 Twin-prime editing pattern (*attB* insertion)

HEK3

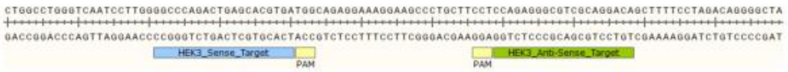

Insertion
Substitution
Deletion : -----

Normal control GGCCCAGACTGAGCACGTGATGGCAGAGGAAAGGAAGCCCTGCTTCCTCCAGAGGGCGTCGCAGGACA

HEK3 / twin-PE / Whole overlap (*attB* insertion)

Insertion
Substitution
Deletion : -----

	Sequence	%
Precise insertion	GGCCCAGACTGAGCACGggcttgtcgacgacggcggtctccgtcgtcaggatcatGAGGGCGTCGCAGGACA<br>*attB* (38bp)	24.24 %
Pure indel	None	
Partial insertion	GGCCCAGACTGAGCACGggcttgtcgacgacggcggtctccgtcgtcaggatcatGCCAGAGGGCGTCGCAGGACA	0.43 %
	GGCCCAGACTGAGCACGTgcttgtcgacgacggcggtctccgtcgtcaggatcatGAGGGCGTCGCAGGACA	0.43 %
	GGCCCAGACTGAGCACG-----------------------------caggatcatGAGGGCGTCGCAGGACA	0.31 %
	GGCCCAGACTGAGCACGTGATGGCAAAGGATggcttgtcgacgacggcggtctccgtcgtcaggatcatGAGGGCGTCGCAGGACA	0.31 %
	GGCCCAGACTGAGCACGTGATGGCAAAGGAAAGGggcttgtcgacgacggggtctccgtcgtcaggatcatGAGGGCGTCGCAGGACA	0.25 %

HEK3 / La-twin-PE / Whole overlap (*attB* insertion)

Insertion
Substitution
Deletion : -----

Precise insertion	GGCCCAGACTGAGCACGggcttgtcgacgacggcggtctccgtcgtcaggatcatGAGGGCGTCGCAGGACA<br>*attB* (38bp)	49.59 %
Pure indel	None	
Partial insertion	GGCCCAGACTGAGCACGggctt-----------------------------------GAGGGCGTCGCAGGACA	0.43 %
	GGCCCAGACTGAGCACGTgcttgtcgacgacggcggtctccgtcgtcaggatcatGAGGGCGTCGCAGGACA	0.39 %
	GGCCCAGACTGAGCACGggcttgtcgacgacggcggtctccgtcgtcaggatcat----GCGTCGCAGGACA	0.33 %
	GGCCCAGACTGAGCACGggcttgtcgacgacggcggtctccgtcgtcaggatcatGCAGGGCGTCGCAGGACA	0.33 %
	GGCCCAGACTGAGCACGggcttgtcgacgacggcggtctccgtcgtcagga-----------GGGCGTCGCAGGACA	0.24 %

HEK3 / twin-PEn / Whole overlap (*attB* insertion)

Insertion
Substitution
Deletion : -----

Precise insertion	GGCCCAGACTGAGCACGggcttgtcgacgacggcggtctccgtcgtcaggatcatGAGGGCGTCGCAGGACA<br>*attB* (38bp)	18.21 %
Pure indel	GGCCCAGACTGAGCACG--------------------------------------GAGGGCGTCGCAGGACA	12.29 %
	GGCCCAGACTGAGCAC---------------------------------------GAGGGCGTCGCAGGACA	0.80 %
	GGCCCAGACTGAGCACG-GAT----------------------------------catGAGGGCGTCGCAGGACA	0.73 %
	GGCCCAGACTGAGCACGGTGATGGCAGAGGAAAGGAAGCCCTGCTTCCTCCAGAGGGCGTCGCAGGACA	0.50 %
	GGCCCAGACTGAGCACGAGTGATGGCAGAGGAAAGGAAGCCCTGCTTCCTCCAGAGGGCGTCGCAGGACA	0.28 %
Partial insertion	GGCCCAGACTGAGCAC-ggcttgtcgacgacggcggtctccgtcgtcaggatcatGAGGGCGTCGCAGGACA	1.68 %
	GGCCCAGACTGAGCACGTGATGGCAGAGGAAAGGAAGCCCTGCTTCCTCCAggcttgtcgacgacggcggtctccgtcgtcaggatcatGAGG<br>GCGTCGCAGGACA	0.83 %
	GGCCCAGACTGAGCACG--------gacgacggcggtctccgtcgtcaggatcatGAGGGCGTCGCAGGACA	0.66 %
	GGCCCAGACTGAGCACGggcttgtcg------------------------------GAGGGCGTCGCAGGACA	0.58 %
	GGCCCAGACTGAGCACGggcttgtcgacgacggcggtctccg------------tGAGGGCGTCGCAGGACA	0.49 %

HEK3 / La-twin-PEn / Whole overlap (*attB* insertion)

Insertion
Substitution
Deletion : -----

Precise insertion	GGCCCAGACTGAGCACGggcttgtcgacgacggcggtctccgtcgtcaggatcatGAGGGCGTCGCAGGACA<br>*attB* (38bp)	25.00 %
Pure indel	GGCCCAGACTGAGCACG--------------------------------------GAGGGCGTCGCAGGACA	6.26 %
	GGCCCAGACTGAGCAC---------------------------------------GAGGGCGTCGCAGGACA	1.58 %
	GGCCCAGACTGAGCA---------AGGAAAGGAAGCCCTGCTTCCTCCAGAGGGCGTCGCAGGACA	0.36 %
	GGCCCAGACTGAGCACGGTGATGGCAGAGGAAAGGAAGCCCTGCTTCCTCCAGAGGGCGTCGCAGGACA	0.21 %
	GGCCCAGACTGAGCACGCTGATGGCAGAGGAAAGGAAGCCCTGCTTCCTCCAGAGGGCGTCGCAGGACA	0.21 %
Partial insertion	GGCCCAGACTGAGCACGggcttgtcgacgacggcggtctccgtcgtcaggatc--GAGGGCGTCGCAGGACA	1.24 %
	GGCCCAGACTGAGCACGggcttgtcgacgacggcggtctccgtcgtcaggatcatTGATGGCAGAGGAAAGGAAGCCCTGCTTCCTCCAGAGG<br>GCGTCGCAGGACA	1.16 %
	GGCCCAGACTGAGCACGggcttgtcgacgacggcggtctc---------------GAGGGCGTCGCAGGACA	0.99 %
	GGCCCAGACTGAGCACGggcttgtcgacgacggcgg-------------------GAGGGCGTCGCAGGACA	0.79 %
	GGCCCAGACTGAGCACGggcttgtcgacgacggcggtctccg-T-----------GAGGGCGTCGCAGGACA	0.79 %

HEK3 / twin-PE / Homology arm (*attB* insertion)

Insertion
Substitution
Deletion : -----

Precise insertion	GGCCCAGACTGAGCACGggcttgtcgacgacggcggtctccgtcgtcaggatcatGAGGGCGTCGCAGGACA<br>*attB* (38bp)	10.24 %
Pure indel	None	
Partial insertion	GGCCCAGACTGAGCACGCTGAGCACGggcttgtcgacgacggcggtctccgtcgtcaggatcatGAGGGCGTCGCAGGACA	0.47 %
	GGCCCAGACTGAGCACGCggcttgtcgacgaGCACGggcttgtcgacgacggcggtctccgtcgtcaggatcatGAGGGCGTCGCAGGACA<br>Sense insertion / Anti-Sense insertion	0.29 %
	GGCCCAGACTGAGCACGCgcttgtcgacgacggcggtctccgtcgtcaggatcat-----------GAGGGC-GTCTCCGTCGTCAGGATCATGAGGGC<br>GTCGCAGGACA<br>Sense insertion / Anti-Sense insertion	0.20 %
	GGCCCAGACTGAGCACGCggcttgtcgacgacggcggtctccgtcgtcaggatcatGAAGGCGTCGCAGGACA	0.03 %
	GGCCCAGACTGAGCACGCggcttgtcgacgacggcgCtctccgtcgtcaggatcatGAGGGCGTCGCAGGACA	0.02 %

HEK3 / La-twin-PE / Homology arm (*attB* insertion)

Insertion
Substitution
Deletion : -----

	Sequence	Frequency
Precise insertion	GGCCCAGACTGAGCACGggcttgtcgacgacggcggtctccgtcgtcaggatcatGAGGGCGTCGCAGGACA<br>*attB* (38bp)	7.31 %
Pure indel	None	
Partial insertion	GGCCCAGACTGAGCACGCggcttgtcgacgacggcggtctccgtcgtcaggatcatGAGGGCGTGAGGGCGTCGCAGGACA	0.32 %
	GGCCCAGACTGAGCACGCggcttgtcgacgacggcggtctccgtcgtcaggatcatGAGGGCGTCGCAGGGAGGGCGTCGCAGGACA	0.31 %
	GGCCCAGACTGAGCACGCggcttgtcgacgacggcggtctccgtcgtcaggatcCtGAGGGCGTCGCAGGACA	0.05 %
	GGCCCAGACTGAGCACGCggcttgtcgacgacggTggtctccgtcgtcaggatcatGAGGGCGTCGCAGGACA	0.01 %
	GGCCCAGACTGAGCACGCggcttgtcgacgacggcggCctccgtcgtcaggatcatGAGGGCGTGAGGGCGTCGCAGGACA	0.01 %

HEK3 / twin-PEn / Homology arm (*attB* insertion)

Insertion
Substitution
Deletion : -----

	Sequence	Frequency
Precise insertion	GGCCCAGACTGAGCACGggcttgtcgacgacggcggtctccgtcgtcaggatcatGAGGGCGTCGCAGGACA<br>*attB* (38bp)	3.52 %
Pure indel	GGCCCAGACTGAGCACG--------------------------------------GAGGGCGTCGCAGGACA	34.10 %
	GGCCCAGACTGAGCAC---------------------------------------GAGGGCGTCGCAGGACA	0.57 %
	GGCCCAGACTGA-----------GCAGAGGAAAGGAAGCCCTGCTTCCTCCAGAGGGCGTCGCAGGACA	0.55 %
	GGCCCAGAC-----------TGGCAGAGGAAAGGAAGCCCTGCTTCCTCCAGAGGGCGTCGCAGGACA	0.38 %
	GGCCCAGACTGAGC-----ATGGCAGAGGAAAGGAAGCCCTGCTTCCTCCAGAGGGCGTCGCAGGACA	0.34 %
Partial insertion	GGCCCAGACTGAGCACGGCACGggcttgtcgacgacggcggtctccgtcgtcaggatcatGAGGGCGTCGCAGGACA	0.39 %
	GGCCCAGACTGAGCACG-----------gacggcggtctccgtcgtcaggatcatGAGGGCGTCGCAGGACA	0.30 %
	GGCCCAGACTGAGCACG--------------------ctccgtcgtcaggatcatGAGGGCGTCGCAGGACA	0.26 %
	GGCCCAGACTGAGCACG---------acgacggcggtctccgtcgtcaggatcatGAGGGCGTCGCAGGACA	0.19 %
	GGCCCAGACTGAGCACGCCCAGACTGAGCACGggcttgtcgacgacggcggtctccgtcgtcaggatcatGAGGGCGTCGCAGGACA	0.17 %

HEK3 / La-twin-PEn / Homology arm (*attB* insertion)

Insertion
Substitution
Deletion : -----

	Sequence	Frequency
Precise insertion	GGCCCAGACTGAGCACGggcttgtcgacgacggcggtctccgtcgtcaggatcatGAGGGCGTCGCAGGACA<br>*attB* (38bp)	3.57 %
Pure indel	GGCCCAGACTGAGCACG--------------------------------------GAGGGCGTCGCAGGACA	36.12 %
	AGCCCTGGCCT--GGACA	4.02 %
	GGCCCAGACTGAGCAGCGTGATGGCAGAGGAAAGGAAGCCCTGCTTCCTCCAGAGGGCGTCGCAGGACA	0.45 %
	GGCCCAGACTGAGCAC-TGATGGCAGAGGAAAGGAAGCCCTGCTTCCTCCAGAGGGCGTCGCAGGACA	0.31 %
	GGCCCAGACTGAGCACGTGATGGCAGAGGAAAGGAAGCCCTGCTTCCTCCAGGAGGGCGTCGCAGGACA	0.29 %
Partial insertion	GGCCCAGACTGAGCACGggcttgtcgacgacggcggtctccgtcgtcaggatcatGAGGGCGTCGCAGGAGCGAGGGCGTCGCAGGACA	1.36 %
	GGCCCAGACTGAGCACGGCACggcttgtcgacgacggcggtctccgtcgtcaggatcatGAGGGCGTCGCAGGACA	0.42 %
	GGCCCAGACTGAGACTGAGCACGggcttgtcgacgacggcggtctccgtcgtcaggatcatGAGGGCGTCGCAGGACA	0.34 %
	GGCCCAGACTGAGCACGggcttgtTGATGGCAGAGGAAAGGAAGCCCTGCTTCCTCCAGAGGGCGTCGCAGGACA	0.24 %
	GGCCCAGACTGAGCACGggcttgtcgacgacggcggtctccgtcg----------TGATGGCAGAGGAAAGGAAGCCCTGCTTCCTCCAGAGGGC GTCGCAGGACA	0.22 %

HEK3 / twin-PE / Partial overlap (*attB* insertion)

Insertion
Substitution
Deletion : -----

	Sequence	Frequency
Precise insertion	GGCCCAGACTGAGCACGggcttgtcgacgacggcggtctccgtcgtcaggatcatGAGGGCGTCGCAGGACA<br>*attB* (38bp)	15.95 %
Pure indel	None	
Partial insertion	GGCCCAGACTGAGCACGggcttgtcgacgacggcggtctcc--cCtc-----ca-GAGGGCGTCGCAGGACA	0.21 %
	GGCCCAGACTGAGCACGggcttgtCGAcgacgacggcggtctccgtcgtcaggatcatGAGGGCGTCGCAGGACA	0.19 %
	GGCCCAGACTGAGCACGggcttgtcgacgacggcg---------gtcgtcaggatcatGAGGGCGTCGCAGGACA	0.19 %
	GGCCCAGACTGAGCACGggcttgtcgacgacggcggtctcCGTCGTGcgtcgtcaggatcatGAGGGCGTCGCAGGACA	0.18 %
	GGCCCAGACTGAGCACG--------------ggcggtctccgtcgtcaggatcatGAGGGCGTCGCAGGACA	0.17 %

HEK3 / La-twin-PE / Partial overlap (*attB* insertion)

Insertion
Substitution
Deletion : -----

Precise insertion GGCCCAGACTGAGCACGggcttgtcgacgacggcggtctccgtcgtcaggatcatGAGGGCGTCGCAGGACA 47.51 %

attB (38bp)

Pure indel None

Partial insertion

GGCCCAGACTGAGCACGggcttgtcgacgacggcggtctccgtcgt--g---ca-GAGGGCGTCGCAGGACA 1.00 %
GGCCCAGACTGAGCACGggcttgtcgacgac------------------------GAGGGCGTCGCAGGACA 0.96 %
GGCCCAGACTGAGCACGggcttgtcgacgac---GGACA 0.81 %
GGCCCAGACTGAGCACGggcttgtcgacgacggcggtctc---------------------------cggcggtctccgtcgtcaggatcatGAGGGCGTCGCAGGACA 0.69 %

Sense insertion Anti-Sense insertion

GGCCCAGAC------------------------------------gtcgtcaggatcatGAGGGCGTCGCAGGACA 0.51 %

HEK3 / twin-PEn / Partial overlap (*attB* insertion)

Insertion
Substitution
Deletion : -----

Precise insertion GGCCCAGACTGAGCACGggcttgtcgacgacggcggtctccgtcgtcaggatcatGAGGGCGTCGCAGGACA 13.78 %

attB (38bp)

Pure indel

GGCCCAGACTGAGCACG---------------------------------CATGAGGGCGTCGCAGGACA 2.59 %
GGCCCAGAC--------TGATGGCAGAGGAAAGGAAGCCCTGCTTCCTCCAGAGGGCGTCGCAGGACA 1.20 %
GGCCCAGACTGAGCACGTCAT-----------------------------------GAGGGCGTCGCAGGACA 0.94 %
GGCCCAGACTGAGCACG---TGGCAGAGGAAAGGAAGCCCTGCTTCCTCCAGAGGGCGTCGCAGGACA 0.75 %
GGCCCAGACTGAGCACGCGTGATGGCAGAGGAAAGGAAGCCCTGCTTCCTCCAGAGGGCGTCGCAGGACA 0.31 %

Partial insertion

GGCCCAGACTGAGCACGggcttgtcgac------------------------GAGGGCGTCGCAGGACA 2.71 %
GGCCCAGACTGAGCACGggcttgtcgacgacggcgCt------------------GAGGGCGTCGCAGGACA 2.39 %
GGCCCAGACTGAGCACGggcttgtcgacgacggcggtctccgtcgt---------GAGGGCGTCGCAGGACA 1.98 %
GGCCCAGACTGAGCACGggcttgtcgacgacg------------------------GAGGGCGTCGCAGGACA 1.61 %
GGCCCAGACTGAGCACG---------------------tccgtcgtcaggatcatGAGGGCGTCGCAGGACA 1.60 %

HEK3 / La-twin-PEn / Partial overlap (*attB* insertion)

Insertion
Substitution
Deletion : -----

Precise insertion GGCCCAGACTGAGCACGggcttgtcgacgacggcggtctccgtcgtcaggatcatGAGGGCGTCGCAGGACA 14.82 %

attB (38bp)

Pure indel

GGCCCAGACTGAGCACG------------------------------------GAGGGCGTCGCAGGACA 2.49 %
GGCCCAGACTGAGCACGTGATGGCAGAGGAAAGGAAGCCCTGCTTCCTCCAAGAGGGCGTCGCAGGACA 0.54 %
GGCCCAGACTGAGCACGTGATGGCAGAGGAAAGGAAGCCCTGCTTCCTCCA----GCGTCGCAGGACA 0.32 %
GGCCCAGACTGAGCACGTGATGGCAGAGGAAAGGAAGCCCTGCTTCCTCCATGAGGGCGTCGCAGGACA 0.26 %
GGCCCAGACTGAGCACGTGATGGCAGAGGAAAGGAAGCCCTGCTTCCTCCATCATGAGGGCGTCGCAGGACA 0.18 %

Partial insertion

GGCCCAGACTGAGCA---------------cgacggcggtctccgtcgtcaggatcatGAGGGCGTCGCAGGACA 4.13 %
GGCCCAGACTGAGCACGggcttgtcgacgacggcggtct---------------GAGGGCGTCGCAGGACA 2.52 %
GGCCCAGACTGAGCACGggcttgtcgacgacggcggtctccgtcgt---------GAGGGCGTCGCAGGACA 2.37 %
GGCCCAGACTGAGCACGggcttgtcgacgacggcggtctccgtcgt---------TGATGGCAGAGGAAAGGAAGCCCTGCTTCCTCCAGAGGGC GTCGCAGGACA 2.21 %
GGCCCAGACTGAGCACGggcttgtcgacgacggcggtct---------------GAGGGCGTCGCAGGACA 1.78 %

FANCF Twin-prime editing pattern (*attB* insertion)

FANCF

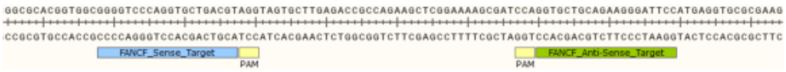

Insertion
Subtitution
Deletion : -----

Normal control GGGGTCCCAGGTGCTGACGTAGGTAGTGCTTGAGACCGCCAGAAGCTCGGAAAAGCGATCCAGGTGCTGCAGAAGGGATTCC

FANCF / twin-PE / Whole overlap (*attB* insertion)

Insertion
Substitution
Deletion : -----

Precise insertion	GGGGTCCCAGGTGCTGAatgatcctgacgacggagaccgccgtcgtcgacaagccGCTGCAGAAGGGATTCC<br>*attB* (38bp)	23.31 %
Pure indel	GGGGTCCCAGGTGCTGACGT----------------------AGCTCGGAAAAGCGATCCAGGTGCTGCAGAAGGGATTCC	0.56 %
Partial insertion	GGGGTCCCAGGTGCTGACGTAGGTAGTGCTTGAGACCGCC---gccgtcgtcgacaagccGCTGCAGAAGGGATTCC	1.88 %
	GGGGTCCCAGGTGCTGAatgatcctgacgacg-------------------------------------GAGACCGCCAGAAGCTCGGAAAAGCGATCCAGGTGCTGCAGAAGGGATTCC	0.48 %
	GGGGTCCCAGGTGCTGA-----------------------------CcaagccGCTGCAGAAGGGATTCC	0.44 %
	GGGGTCCCAGGTGCTGA---------------gagaccgccgtcgtcgacaagccGCTGCAGAAGGGATTCC	0.22 %
	GGGGTCCCAGGTG----CatgatcctgacgacggagaccgccgtcgtcgacaagccGCTGCAGAAGGGATTCC	0.22 %

FANCF / La-twin-PE / Whole overlap (*attB* insertion)

Insertion
Substitution
Deletion : -----

Precise insertion	GGGGTCCCAGGTGCTGAatgatcctgacgacggagaccgccgtcgtcgacaagccGCTGCAGAAGGGATTCC<br>*attB* (38bp)	36.23 %
Pure indel	None	0.56 %
Partial insertion	GGGGTCCCAGGTGCTGACGTAGGTAGTGCTTGAGACCGCC---gtcgtcgacaagccGCTGCAGAAGGGATTCC	1.25 %
	GGGGTCCCAGGTGCTGAatgatcctgacgacggagaccgc---------------atgatcctgacgacggagaccgccgtcgtcgacaagccGCTGCAGAAGGGATTCC	0.27 %
	GGGGTCCCAGGTGCTGAatgatcctgacgacggagaccgccgtcgtc--------------ctgacgacggagaccgccgtcgtcgacaagccGCTGCAGAAGGGATTCC<br>Sense insertion / Anti-Sense insertion	0.11 %
	GGGGTCCCAGGTGCTGAatgatcctgacgacg------------------------GAGACCGCCAGAAGCTCGGAAAAGCGATCCAGGTGCTGCAGAAGGGATTCC	0.11 %
	GGGGTCCCAGGTGCTGAatgatcctgacgacggagaccgccgtcgtcgacaagGTGCTGCAGAAGGGATTCC	0.08 %

FANCF / twin-PEn / Whole overlap (*attB* insertion)

Insertion
Substitution
Deletion : -----

Precise insertion	GGGGTCCCAGGTGCTGAatgatcctgacgacggagaccgccgtcgtcgacaagccGCTGCAGAAGGGATTCC<br>*attB* (38bp)	16.16 %
Pure indel	GGGGTCCCAGGTGCTGA---GCTGCAGAAGGGATTCC	7.01 %
	GGGGTCCCAGGTGCTGA---GCCGCTGCAGAAGGGATTCC	1.15 %
	GGGGTC----------ACGTAGGTAGTGCTTGAGACCGCCAGAAGCTCGGAAAAGCGATCCAGGTGCTGCAGAAGGGATTCC	0.63 %
	GGGGTCCCAGGTGCTGAATCGTAGGTAGTGCTTGAGACCGCCAGAAGCTCGGAAAAGCGATCCAGGTGCTGCAGAAGGGATTCC	0.37 %
	GGGGTCCC--------ACGTAGGTAGTGCTTGAGACCGCCAGAAGCTCGGAAAAGCGATCCAGGTGCTGCAGAAGGGATTCC	0.35 %
Partial insertion	GGGGTCCCAGGTGCTGAatgatcctgacgacggagaccgccgtcgtcgacaagccCGTAGGTAGTGCTTGAGACCGCCAGAAGCTCGGAAAAGCGATCCAGGTGCTGCAGAAGGGATTCC	0.88 %
	GGGGTCCCAGGTGCTGACGTAGGTAGTGCTTGAGACCGCC---gtcgtcgacaagccGCTGCAGAAGGGATTCC	0.77 %
	GGGGTCCCAGGTGCTG-atgatcctgacgacggagaccgccgtcgtcgacaagccGCTGCAGAAGGGATTCC	0.61 %
	GGGGTCCCAGGTGCTGAatgatcctgacg-------------------------CGTAGGTAGTGCTTGAGACCGCCAGAAGCTCGGAAAAGCGATCCAGGTGCTGCAGAAGGGATTCCGCTGCAGAAGGGATTCC	0.54 %
	GGGGTCCCAGGTGCTGAatgatcctgacgacggagaccgccgtcgtcgacaagc--CTGCAGAAGGGATTCC	0.46 %

FANCF / La-twin-PEn / Whole overlap (*attB* insertion)

Insertion
Substitution
Deletion : -----

Precise insertion	GGGGTCCCAGGTGCTGAatgatcctgacgacggagaccgccgtcgtcgacaagccGCTGCAGAAGGGATTCC<br>*attB* (38bp)	26.09 %
Pure indel	GGGGTCCCAGGTGCTGA---GCTGCAGAAGGGATTCC	5.06 %
	GGGGTCCCAGGTGCTGA---AGCTGCAGAAGGGATTCC	1.14 %
	GGGGTCCCAGGTGCTGACGTAGGTAGTGCTTGAGACCGCCAGAAGCTCGGAAAAGCGATCCAGGTGGCTGCAGAAGGGATTCC	0.14 %
Partial insertion	GGGGTCCCAGGTGCTGACGTAGGTAGTGCTTGAGACCGCC---gtcgtcgacaagccGCTGCAGAAGGGATTCC	0.91 %
	GGGGTCCCAGGTGCTGACGTAGGTAGTGCTTGAGACCGCCAGAAGCTCGGAAAAGCGATCCAGGTatgatcctgacgacggagaccgccgtcgtcgacaagccGCTGCAGAAGGGATTCC	0.67 %
	GGGGTCCCAGGTGCTGAatgatcctgacgacggagaccgccgtcgtcgacaa---GCTGCAGAAGGGATTCC	0.62 %
	GGGGTCCCAGGTGCTGAatgatcctg-----------------------------GCTGCAGAAGGGATTCC	0.55 %
	GGGGTCCCAGGTGCTGAatgatcctgacgacggagaccgccgtcgtcgacaagccAGCTGCAGAAGGGATTCC	0.49 %

FANCF / twin-PE / Homology arm (*attB* insertion)

Insertion
Substitution
Deletion : -----

Precise insertion	GGGGTCCCAGGTGCTGAatgatcctgacgacggagaccgccgtcgtcgacaagccGCTGCAGAAGGGATTCC<br>*attB* (38bp)	7.61 %
Pure indel	GGGGTCCCAGGTGCTGAAGTA-GTAGTGCTTGAGACCGCCAGAAGCTCGGAAAAGCGATCCAGGTGCTGCAGAAGGGATTCC	0.04 %
	GGGGTCCCAGGTGCTGACGTAGGTAGTGCTTGAGACCGCCAGAAGCTCGG----GCGATCCCAGGTGCTGCAGAAGGGATTCC	0.03 %
Partial insertion	GGGGTCCCAGGTGCTGACGTAGGTAGTGCTTGAGACCGCC---gtcgtcgacaagccGCTGCAGAAGGGATTCC	0.77 %
	GGGGTCCCAGGTGCTGAGtgatcctgacgacggagaccgccgtcgtcgacaagccGCTGCAGAAGGGATTCC	0.42 %
	GGGGTCCCAGGTGCTGAatgatcctgGcgacggagaccgccgtcgtcgacaagccGCTGCAGAAGGGATTCC	0.40 %
	GGGGTCCCAGGTGCTGACGTAGGTAGTGCTTGAGACCGC------------------------------------gagaccgccgtcgtcgacaagccGCTGCAGAAGGGATTCC	0.33 %
	GGGGTCCCAGGTGCTGAatgGtcctgacgacggagaccgccgtcgtcgacaagccGCTGCAGAAGGGATTCC	0.23 %

FANCF / La-twin-PE / Homology arm (*attB* insertion)

Insertion
Substitution
Deletion : -----

Precise insertion	GGGGTCCCAGGTGCTGAatgatcctgacgacggagaccgccgtcgtcgacaagccGCTGCAGAAGGGATTCC<br>*attB* (38bp)	9.87 %
Pure indel	GGGGTCCCAGGTGCTGACGTAGGTAGTGC--------------------------GCGATCCCAGGTGCTGCAGAAGGGATTCC	0.04 %
	GGGGTCCCAGGTGCTGA---GGCTCCAGGTGCTGCAGAAGGGATTCC	0.01 %
Partial insertion	GGGGTCCCAGGTGCTGAatgatcctgacgacggagaccgccgtcgtcgacaGgccGCTGCAGAAGGGATTCC	0.99 %
	GGGGTCCCAGGTGCTGAatgatccCgacgacggagaccgccgtcgtcgacaagccGCTGCAGAAGGGATTCC	0.75 %
	GGGGTCCCAGGTGCTGAGtgatcctgacgacggagaccgccgtcgtcgacaagccGCTGCAGAAGGGATTCC	0.34 %
	GGGGTCCCAGGTGCTGAatgGtcctgacgacggagaccgccgtcgtcgacaagccGCTGCAGAAGGGATTCC	0.31 %
	GGGGTCCCAGGTGCTGAatgatcctgGcgacggagaccgccgtcgtcgacaagccGCTGCAGAAGGGATTCC	0.28 %

FANCF / twin-PEn / Homology arm (*attB* insertion)

Insertion
Substitution
Deletion : -----

Precise insertion	GGGGTCCCAGGTGCTGAatgatcctgacgacggagaccgccgtcgtcgacaagccGCTGCAGAAGGGATTCC	2.72 %
Pure indel	GGGGTCCCAGGTGCTGAC--GCTGCAGAAGGGATTCC	63.87 %
	GGGGTCCCAGGTGCTGA---GCTGCAGAAGGGATTCC	1.23 %
	G--------------------------------GACCGCCAGAAGCTCGGAAAAGCGATCCAGGTGCTGCAGAAGGGATTCC	0.70 %
	GGGGTCCCAGGTGCTGAATGCGTAGGTAGTGCTTGAGACCGCCAGAAGCTCGGAAAAGCGATCCAGGTGCTGCAGAAGGGATTCC	0.27 %
	GGGGTCCCAGGTGCTGAATCGTAGGTAGTGCTTGAGACCGCCAGAAGCTCGGAAAAGCGATCCAGGTGCTGCAGAAGGGATTCC	0.25 %
Partial insertion	GGGGTCCCAGGTGCTGAatgatcctgac--------------------------GCTGCAGAAGGGATTCC	0.33 %
	GGGGTCCCAGGTGCTGAatgatcctgacgacggagaccgccgtcgtcgacaagccCGCTGGCTGCAGAAGGGATTCC	0.23 %
	GGGGTCCCAGGTGCTGAatgatcc------------------------------GCTGCAGAAGGGATTCC	0.23 %
	GGGGTCCCAGGTGCTGACGTAGGTAGTGCTTGAGACCGCC---gtcgtcgacaagccGCTGCAGAAGGGATTCC	0.16 %
	GGGGTCCCAGGTGCTGAatgatcctgacgacggagaccgccgtcgtcgacaagccGCTGCAGAAGGGATTCGTAGGTAGTGCTTGAGACCGCCAGAAGCTCGGAAAAGCGATCCAGGTGCTGCAGAAGGGATTCC	0.16 %

Insertion
Substitution
Deletion : -----

FANCF / La-twin-PEn / Homology arm (*attB* insertion)

	Sequence	%
Precise insertion	GGGGTCCCAGGTGCTGAatgatcctgacgacggagaccgccgtcgtcgacaagccGCTGCAGAAGGGATTCC	2.21 %
Pure indel	GGGGTCCCAGGTGCTGA--GCTGCAGAAGGGATTCC	46.04 %
	GGGGTCCCAGGTGCTGAC--GCTGCAGAAGGGATTCC	0.55 %
	GGGGTCCCAGGTGCTGAGTGAG--GCTGCAGAAGGGATTCC	0.52 %
	GGGGTCCCAGGTGCTGAGCC--GCTGCAGAAGGGATTCC	0.49 %
	GGGGTCCCAGGTGCTGAA--GCTGCAGAAGGGATTCC	0.42 %
Partial insertion	GGGGTCCCAGGTGCTGAatgatcctgacgacggagaccgccgtcgtcgacaagccGCTGCAGAAGGGATTGCTGCAGAAGGGATTCC	0.45 %
	GGGGTCCCAGGTGCTGAatgatcctgacgacggagaccgccgtcgtcgacaagccGCTGCAGAAGCTGCAGAAGGGATTCC	0.24 %
	GGGGTCCCAGGTGCTGAatgatcctgacgac------------------------GCTGCAGAAGGGATTCC	0.22 %
	GGGGTCCCAGGTGCTGAatgatcctgacgacggagaccgccgtcgtcgacaagccGCTGCAGCTGCAGAAGGGATTCC	0.15 %
	GGGGTCCCAGGTGCTGACGTAGGTAGTGCTTGAGACCGCC---gtcgtcgacaagccGCTGCAGAAGGGATTCC	0.14 %

Insertion
Substitution
Deletion : -----

FANCF / twin-PE / Partial overlap (*attB* insertion)

	Sequence	%
Precise insertion	GGGGTCCCAGGTGCTGAatgatcctgacgacggagaccgccgtcgtcgacaagccGCTGCAGAAGGGATTCC	18.33 %
Pure indel	None	
Partial insertion	GGGGTCCCAGGTGCTGACGTAGGTAGTGCTTGAGACCGCC---gtcgtcgacaagccGCTGCAGAAGGGATTCC	1.31 %
	GGGGTCCCAGGTGCTGAatgatcctgacgacggagac-----------------------------cgacggagaccgccgtcgtcgacaagccGCTGCAGAAGGGATTCC (Sense insertion / Anti-Sense insertion)	0.51 %
	GGGGTCCCAGGTGCTGAatgatcctgacgacggagaccgcCGTcgtcgtcgacaagccGCTGCAGAAGGGATTCC	0.44 %
	GGGGTCCCAGGTGCTGAatgatcct---gacggagaccgccgtcgtcgacaagccGCTGCAGAAGGGATTCC	0.40 %
	GGGGTCCCAGGTGCTGAatgatcctgacgacggagaccgccgtG-------------------------cggagaccgccgtcgtcgacaagccGCTGCAGAAGGGATTCC (Sense insertion / Anti-Sense insertion)	0.35 %

Insertion
Substitution
Deletion : -----

FANCF / La-twin-PE / Partial overlap (*attB* insertion)

	Sequence	%
Precise insertion	GGGGTCCCAGGTGCTGAatgatcctgacgacggagaccgccgtcgtcgacaagccGCTGCAGAAGGGATTCC	31.20 %
Pure indel	GGGGTCCCAGGTGCTGACGTAGGTAGTGCTTGAGACCGCCAGAAGCTCGGAAAAGCGATCCAGAAGCTGCTGCAGAAGGGATTCC	0.03 %
	GGGGTCCCAGGTGCTGACG-----------GAGACCGCCAGAAGCTCGGAAAAGCGATCCAGGTGCTGCAGAAGGGATTCC	0.01 %
Partial insertion	GGGGTCCCAGGTGCTGACGTAGGTAGTGCTTGAGACCGCC---gtcgtcgacaagccGCTGCAGAAGGGATTCC	1.59 %
	GGGGTCCCAGGTGCTGA-------------cggagaccgccgtcgtcgacaagccGCTGCAGAAGGGATTCC	0.85 %
	GGGGTCCCAGGTGCTGAatgatcct---gacggagaccgccgtcgtcgacaagccGCTGCAGAAGGGATTCC	0.74 %
	GGGGTCCCAGGTGCTGAatgatcctgacgacggagaccgccg----------------------------gagaccgccgtcgtcgacaagccGCTGCAGAAGGGATTCC (Sense insertion / Anti-Sense insertion)	0.47 %
	GGGGTCCCAGGTGCTGAatgatcctgacgacggagaccgccgtcg-------------------------cggagaccgccgtcgtcgacaagccGCTGCAGAAGGGATTCC (Sense insertion / Anti-Sense insertion)	0.35 %

Insertion
Substitution
Deletion : -----

FANCF / twin-PEn / Partial overlap (*attB* insertion)

	Sequence	%
Precise insertion	GGGGTCCCAGGTGCTGAatgatcctgacgacggagaccgccgtcgtcgacaagccGCTGCAGAAGGGATTCC	7.91 %
Pure indel	GGGGTCCCAGGTGCTGA--GCTGCAGAAGGGATTCC	5.78 %
	GGGGTCCCAGGTGCTGAATCGTAGGTAGTGCTTGAGACCGCCAGAAGCTCGGAAAAGCGATCCAGGTGCTGCAGAAGGGATTCC	0.71 %
	GGGGTCCCAGGTGCTGACGTAGGTAGTGCTTGAGACCGCCAGAAGCTCG-------------GCTGCAGAAGGGATTCC	0.35 %
	GGGGTCCCAG-------------GTGCTTGAGACCGCCAGAAGCTCGGAAAAGCGATCCAGGTGCTGCAGAAGGGATTCC	0.33 %
	GGGGTCCCAGGTGCTGAACGTAGGTAGTGCTTGAGACCGCCAGAAGCTCGGAAAAGCGATCCAGGTGCTGCAGAAGGGATTCC	0.31 %
Partial insertion	GGGGTCCCAGGTGCTGA---------acgacggagaccgccgtcgtcgacaagccGCTGCAGAAGGGATTCC	1.59 %
	GGGGTCCCAGGTGCTGAatgatcct---gacggagaccgccgtcgtcgacaagccGCTGCAGAAGGGATTCC	1.50 %
	GGGGTCCCAGGTGCTGAatgatcctgacg--------------------------CGTAGGTAGTGCTTGAGACCGCCAGAAGCTCGGAAAAGCGATCCAGGTGCTGCAGAAGGGATTCC	1.17 %
	GGGGTCCCAGGTGCTGA-------------------ccgTcgtGgtcgacaagccGCTGCAGAAGGGATTCC	1.13 %
	GGGGTCCCAGGTGCTGAatgatc--------------------------------GCTGCAGAAGGGATTCC	0.96 %

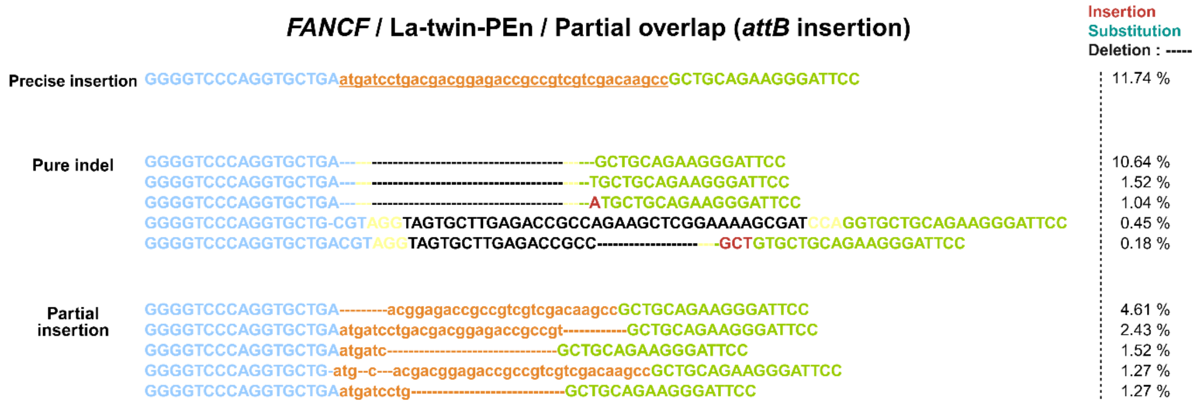

Figure S3. Commonly edited target sequences and corresponding NGS analysis results mediated by SpCas9(H840A/WT)-RT or La-SpCas9(H840A/WT)-RT. For each gene, the target sequences of the pegRNA pairs used with twin-PE(n) or La-twin-PE(n), along with their respective PAM (NGG) sequences, are shown. Within the target DNA sequences, the protospacers and PAM (NGG) sequences corresponding to each sense and antisense pegRNA are colored in light blue, light green, and yellow, respectively. In the edited products, the frequencies (%) of precise insertions, indels (insertions/deletions), and substitutions, as determined by NGS analysis, are indicated to the right of each sequence. In the sequencing results, red nucleotides represent insertions, dashed lines indicate deletions, and blue nucleotides denote substitutions. Orange highlights correspond to precisely or partially inserted *attB*/*attP* sequences.

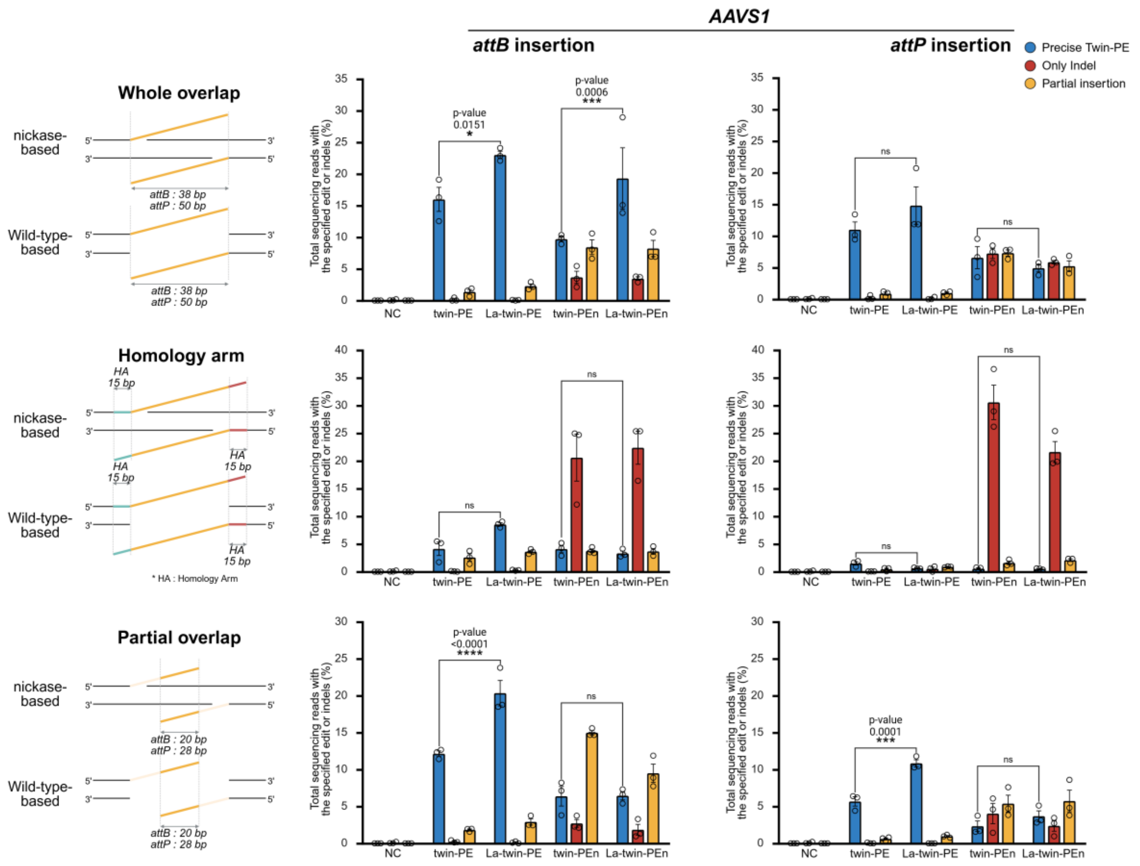

Figure S4. Optimization of twin prime editing components and insertion of various sequences (*attB*/*attP*) at the *AAVS1* locus in human-derived cells. Comparison of twin prime editing efficiency (%) for insertion of a 38 bp *attB* or a 50 bp *attP* Bxb1 recognition sequence at the *AAVS1* locus in human-derived cells (HEK293FT) using various pegRNA overlap strategies. A comparative analysis was conducted using prime editors based on either nickase or wild-type SpCas9 (twin-PE(n)), and those fused with the La domain (La-twin-PE(n)). The pegRNA designs were categorized into three types: (i) Whole overlap pairs, in which the entire insertion sequence is fully complementary in both pegRNAs; (ii) Homology arm pairs, where each pegRNA includes the intended insertion sequence along with flanking homology arms; and (iii) Partial overlap pairs, which share only a 20 bp segment of the insertion sequence between the two pegRNAs. Editing outcomes were categorized as precise twin-PE (%), only indel (%), and partial insertion (%) **(Figure S3)**. Each histogram represents mean ± SEM from three independent experiments. P-values were calculated by two-way ANOVA and Dunnett's test (ns: not significant, *P = 0.0332, **P

= 0.0021, ***P = 0.0002, ****P < 0.0001). RT: reverse transcriptase, La: La domain, NC: negative control; twin-PE: SpCas9(H840A)-RT-based twin-PE; La-twin-PE: La-SpCas9(H840A)-RT-based twin-PE; twin-PEn: SpCas9(WT)-RT-based twin-PE; La-twin-PEn: La-SpCas9(WT)-RT-based twin-PE.

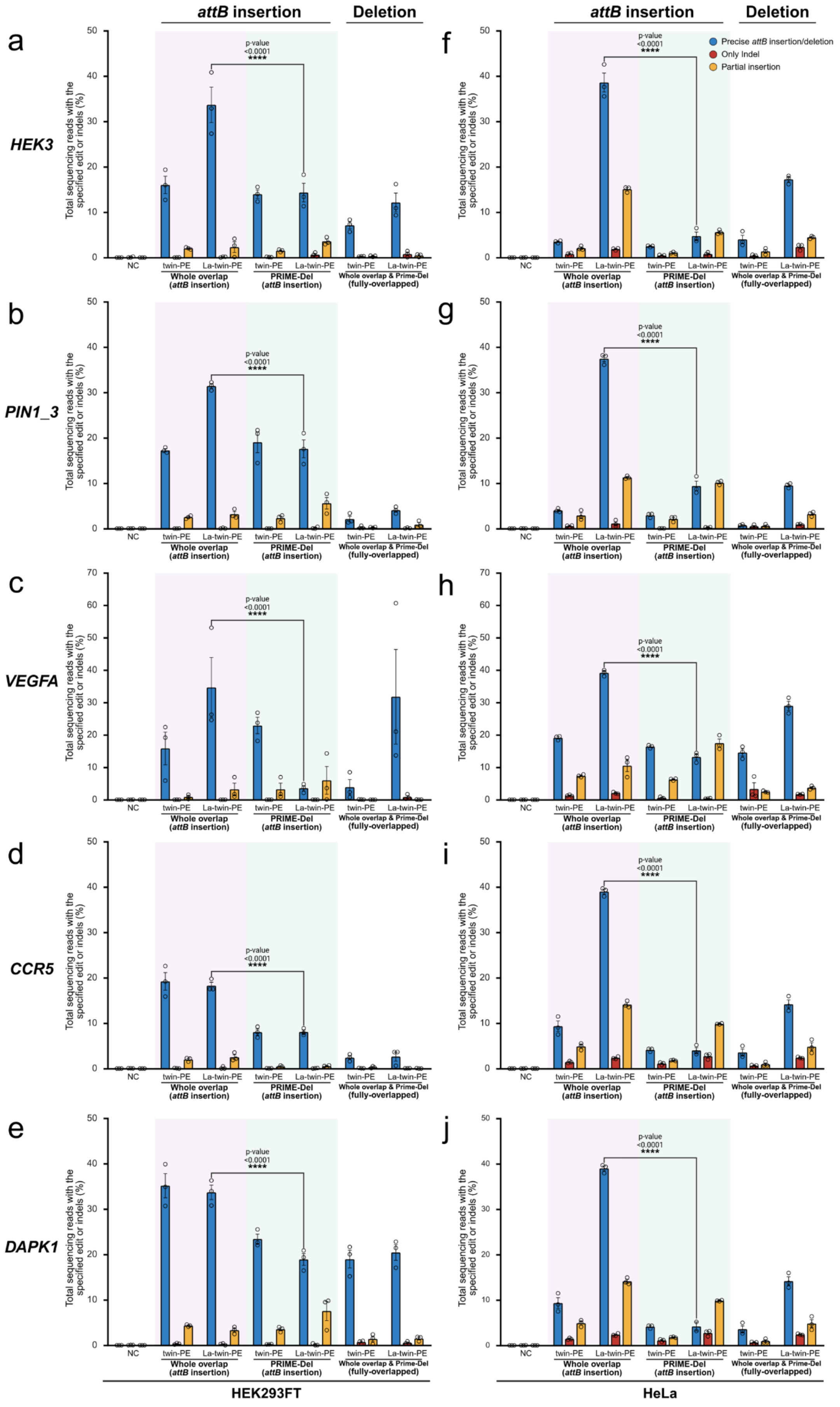

attB insertion
Deletion
a
b
c
d
e
f
g
h
i
j
HEK3
PIN1_3
VEGFA
CCR5
DAPK1
Total sequencing reads with the specified edit or indels (%)
p-value <0.0001 ****
Precise attB insertion/deletion
Only Indel
Partial insertion
NC
twin-PE
La-twin-PE
Whole overlap (attB insertion)
PRIME-Del (attB insertion)
Whole overlap & Prime-Del (fully-overlapped)
HEK293FT
HeLa

Figure S5. Comparative evaluation of PRIME-Del and La-fused twin prime editing for sequence insertion and deletion.

Comparison of twin prime editing efficiency (%) for insertion of a 38 bp *attB* Bxb1 recognition sequence at the *HEK3* locus (a, f), *PIN1* (b, g), *VEGFA* (c, h), *CCR5* (d, i) and *DAPK1* (e, j) loci in HEK293FT (a–e) or HeLa (f–j) cells using a whole overlap design, and PRIME-Del for *attB* insertion or deletion with fully overlapping pegRNA design. Light purple regions indicate *attB* insertion induced by twin-PE, whereas light green regions indicate *attB* insertion induced by PRIME-Del. Uncolored regions indicate deletion induced by fully overlapping pegRNA design. Editing outcomes were categorized as precise twin-PE (%) or deletion (%), only indel (%), and partial insertion (%) **(Figure S3)**. Each histogram represents mean ± SEM from three independent experiments. P-values were calculated by two-way ANOVA and Dunnett's test (ns: not significant, *P = 0.0332, **P = 0.0021, ***P = 0.0002, ****P < 0.0001). NC: negative control; twin-PE: SpCas9(H840A)-RT-based twin-PE; La-twin-PE: La-SpCas9(H840A)-RT-based twin-PE.

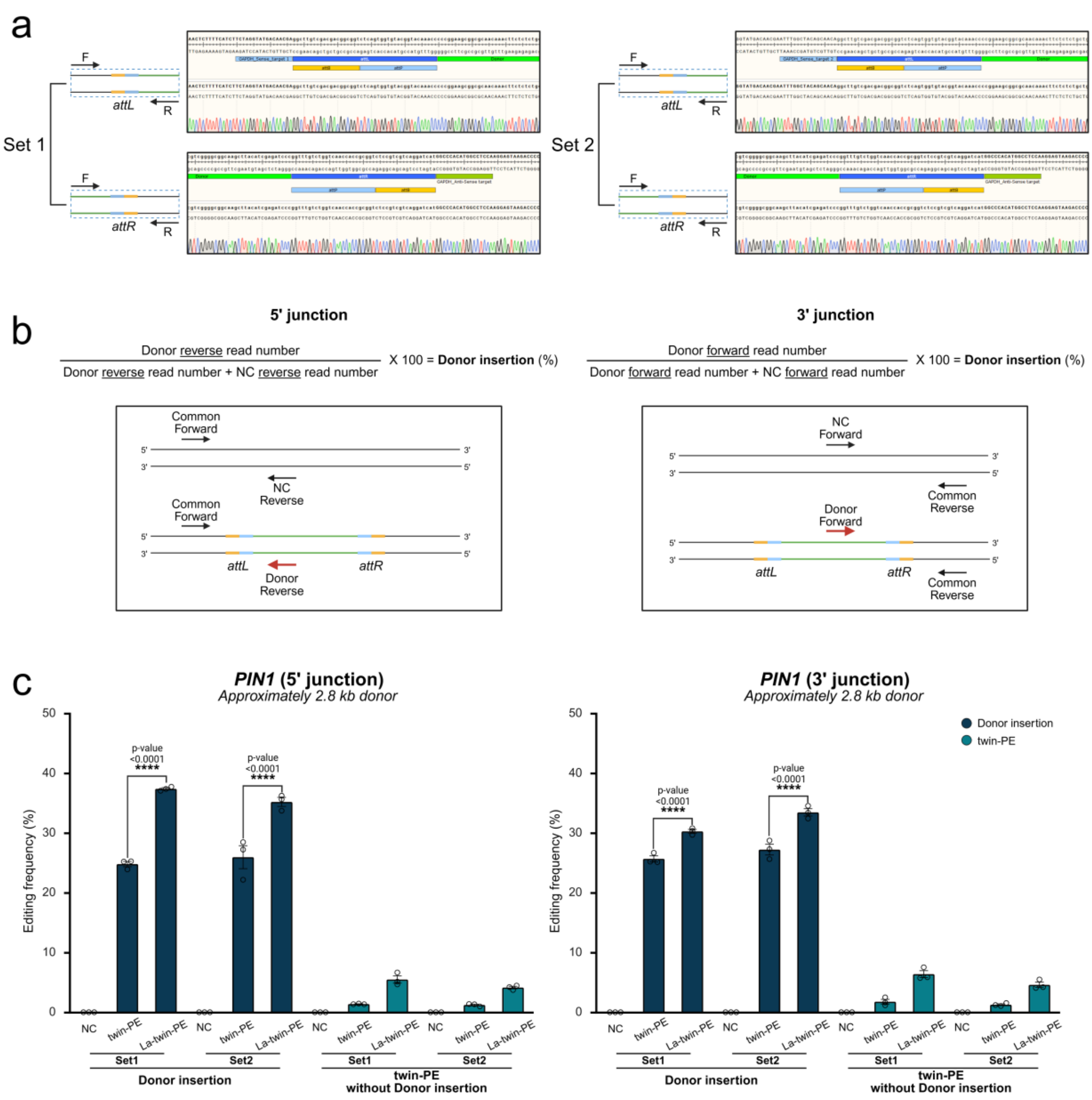

Figure S6. Validation of large gene insertion using optimized twin prime editing in human-derived cells.

(a) Sanger sequencing results from each 5′ and 3′ junction shown in Figure 5, confirming precise donor DNA insertion. The recombined *attL* and *attR* sequences generated by *attB-attP* recombination at the *GAPDH* locus were identified and categorized according to pegRNA pairs (Set 1, Set 2). (b) Schematic illustration of the analysis strategy and calculation method for NGS-based quantification of donor insertion and remaining non-converted twin-PE products at the 5′ and 3′ junctions. (c)

Comparison of donor DNA insertion efficiency following sequential twin prime editing and Bxb1-mediated recombination at *PIN1* locus. *PIN1* Set 1 and Set 2 correspond to *PIN1*_1 and *PIN1*_2 shown in Figure 3a, respectively. NGS-based quantification of donor insertion and remaining non-converted twin-PE products at the 5′ and 3′ junctions of *PIN1* locus in cells treated with each prime editor and the Bxb1 recombinase. donor DNA insertion (%) = donor-specific read count / (donor-specific read count + NC read count) × 100. Each histogram represents mean ± SEM from three independent experiments. P-values were calculated by two-way ANOVA and Dunnett's test (ns: not significant, *P = 0.0332, **P = 0.0021, ***P = 0.0002, ****P < 0.0001). NC: negative control; twin-PE: SpCas9(H840A)-RT-based twin-PE; La-twin-PE: La-SpCas9(H840A)-RT-based twin-PE.

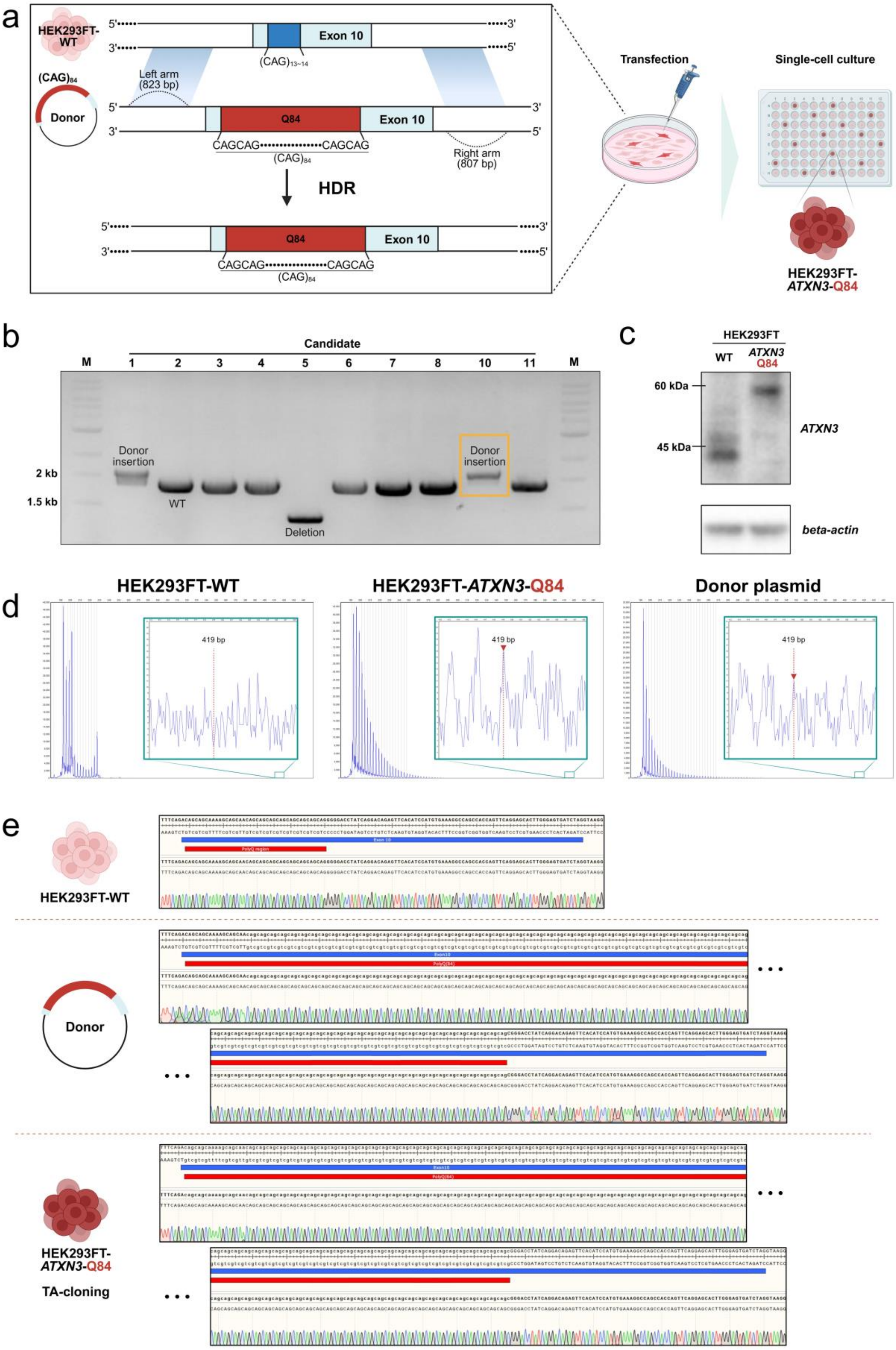
a
HEK293FT-WT
(CAG)84
Donor
5'
3'
Exon 10
(CAG)13~14
Left arm (823 bp)
Q84
CAGCAG••••••••••••••••CAGCAG
Right arm (807 bp)
HDR
Transfection
Single-cell culture
HEK293FT-ATXN3-Q84
b
Candidate
M
1
2
3
4
5
6
7
8
10
11
Donor insertion
2 kb
1.5 kb
WT
Deletion
c
HEK293FT
WT
ATXN3 Q84
60 kDa
45 kDa
ATXN3
beta-actin
d
HEK293FT-WT
HEK293FT-ATXN3-Q84
Donor plasmid
419 bp
e
HEK293FT-WT
Donor
HEK293FT-ATXN3-Q84
TA-cloning

Figure S7. Construction and validation of a human-derived mutant HEK293FT-*ATXN3*-Q84 cell line. (a) Schematic of HDR strategy for inserting 84 CAG repeats into exon 10 of the *ATXN3* gene in HEK293FT cells to generate a human-derived SCA3 model cell line. Donor DNA containing the 84 CAG repeats and left/right homology arms was co-delivered with a SpCas9 and sgRNA expression vector. (b) Gel image showing *ATXN3* PCR amplicons from single-cell clones. Only clones with precise 84 CAG repeat insertion at exon 10 were selected for subculture. Clone in lane 10 (orange box) was selected as the mutant HEK293FT-*ATXN3*-Q84 cell line. (c) Western blot data comparing *ATXN3* protein expression between wild-type and mutant HEK293FT-*ATXN3*-Q84 cell lines using anti-*ATXN3* and anti-*beta-actin* antibodies. (d) TP-PCR analysis comparing CAG repeat expansion in wild-type and mutant HEK293FT cells. PCR results were compared with those of the donor plasmid. (e) Sequencing of the polyQ region in wild-type HEK293FT, donor plasmid, and mutant HEK293FT-*ATXN3*-Q84 cells confirmed the precise 84 CAG repeat insertion in exon 10 of *ATXN3*.

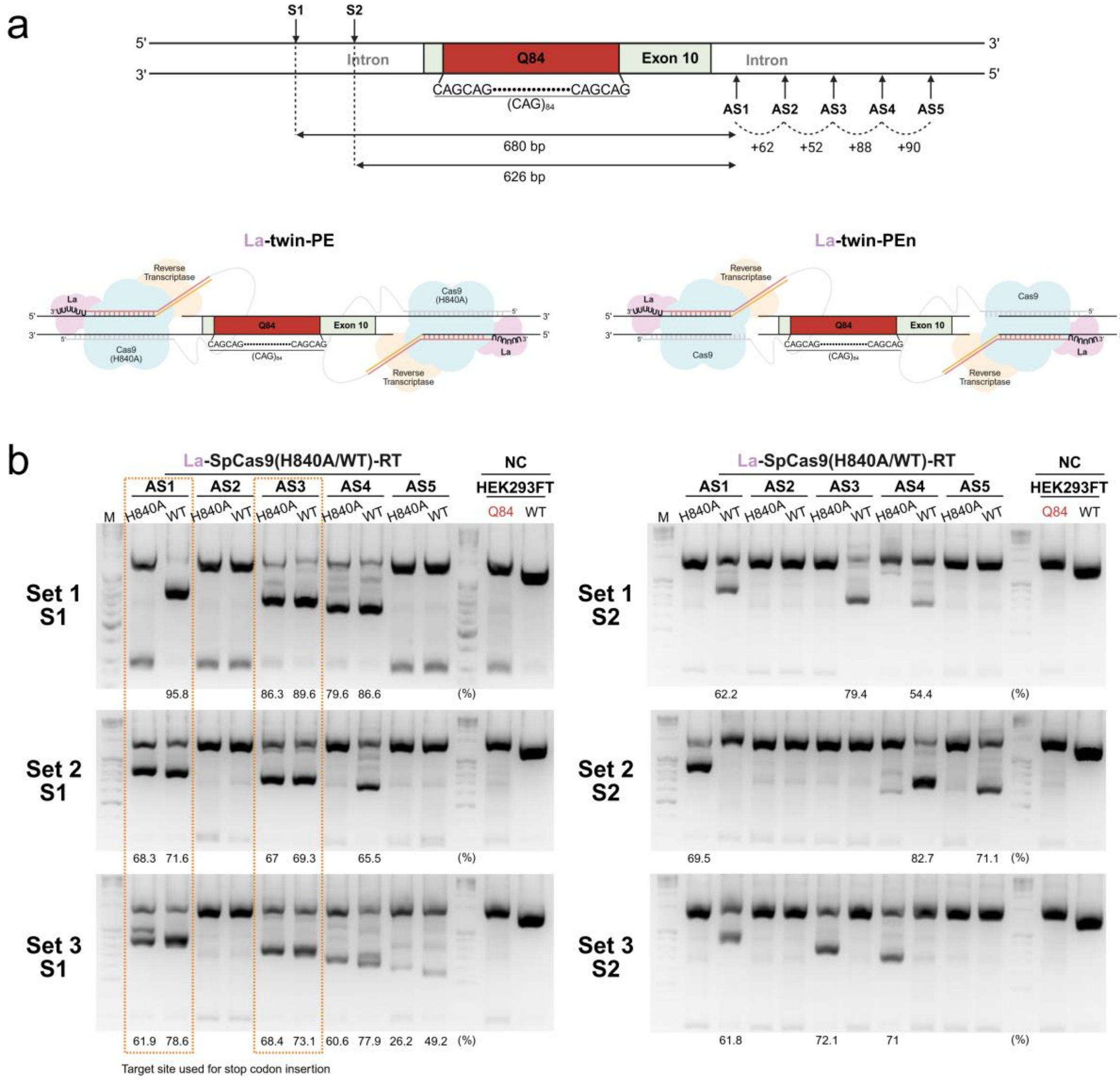

Figure S8. Selection of targeting sites for efficient twin prime editing in human-derived mutant HEK293FT-*ATXN3*-Q84 cells. (a) Schematic of targeting designs for pegRNA pairs (S1+AS1–AS5, S2+AS1–AS5) in upstream and downstream regions of *ATXN3* exon 10 in the mutant HEK293FT-*ATXN3*-Q84 cell line. Candidate pegRNA combinations using La-twin-PE or La-twin-PEn were tested to induce high-efficiency twin prime editing around the polyQ repeats. (b) Gel image comparing PCR amplicons from the *ATXN3* exon 10 region after applying various pegRNA combinations. Lower bands indicate *attB* sequence insertion or simple deletion of exon 10. Orange boxes indicate pegRNA pairs (S1-AS1, S1-AS3) selected for high-efficiency twin prime

editing. Set 1, Set 2, and Set 3 represent independent biological replicates. NC: negative control; H840A: La-twin-PE; WT: La-twin-PEn.

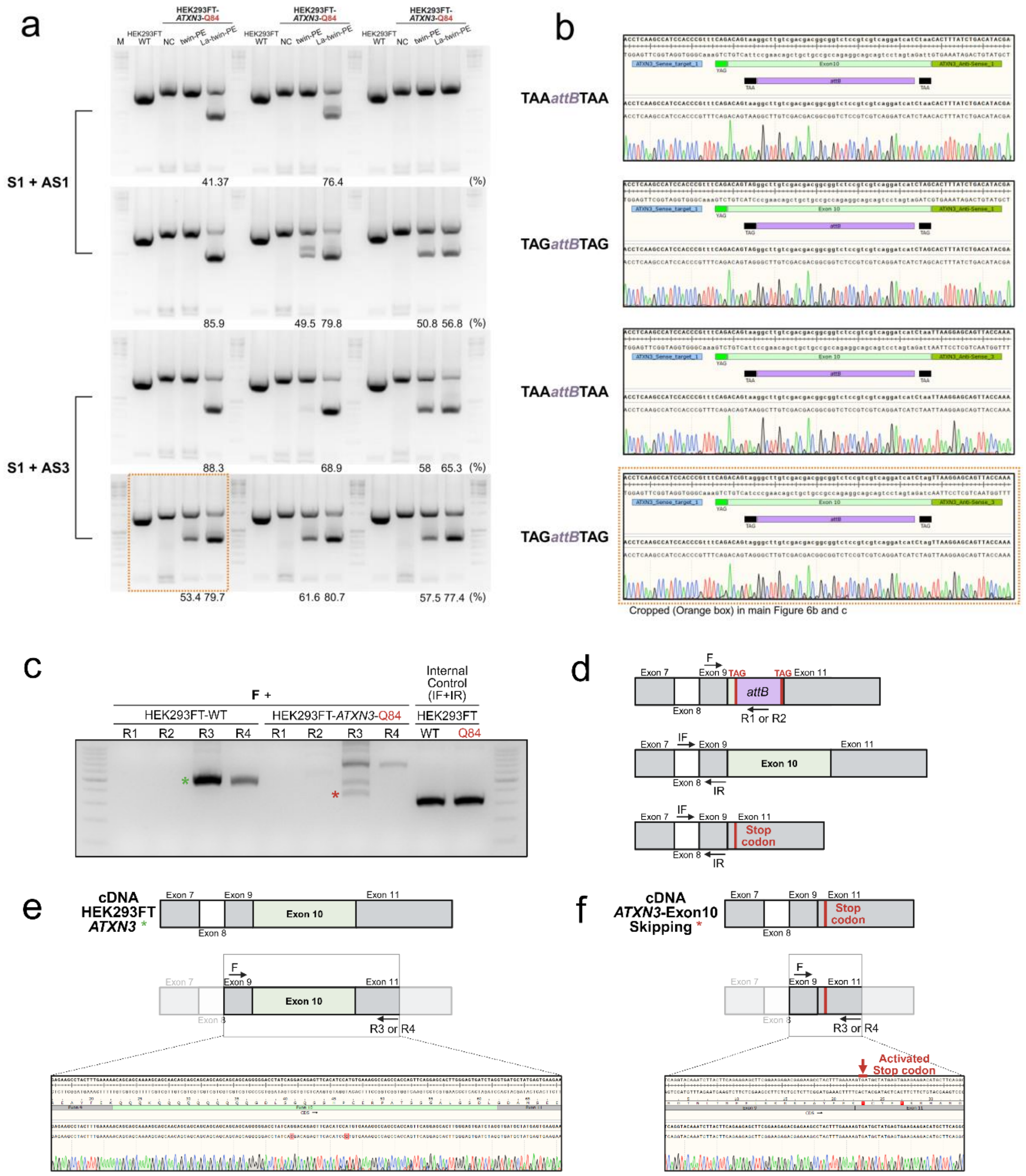

Figure S9. Verification of polyQ removal and early stop codon insertion using optimized twin prime editing in human-derived mutant HEK293FT-*ATXN3*-Q84 cells. (a) Efficiency analysis of polyQ removal and early stop codon insertion by twin prime editing targeting *ATXN3* exon 10 using twin-PE or La-twin-PE. Lower gel bands represent deletion of exon 10 and stop codon insertion. The best pegRNA combinations [S1+AS1 (top), S1+AS3 (bottom)] from Figure S8 were used. Stop

codon insertion efficiency (%) was calculated as size-normalized bottom band intensity / total band intensity. The data highlighted in the orange box are presented in Figure 6. (b) Sanger sequencing results confirming correct stop codon insertion from the shifted bands in (a). (c) Representative gel image illustrating the comparative analysis of cDNA synthesized from transcriptomes derived from wild-type HEK293FT cells and La-twin-PE-treated mutant HEK293FT-*ATXN3*-Q84 cells. The green and red asterisks denote the cDNA amplicons derived from wild-type HEK293FT cells and La-twin-PE-treated mutant HEK293FT-*ATXN3*-Q84 cells, respectively. F and R (R1–R4) indicate the combinations of PCR primers used for cDNA sequencing, where F represents the forward primer and R indicates the reverse primers. IF and IR denote the forward and reverse primers, respectively, used for internal control amplification. (d) Each schematic illustrates the binding positions of DNA primers targeting specific regions of the expected cDNA species shown in panel (c). (e–f) PolyQ repeat sequencing results of *ATXN3* cDNA (green and red asterisks) synthesized from transcriptomes derived from wild-type HEK293FT cells (e) and mutant HEK293FT-*ATXN3*-Q84 cells treated with La-twin-PE (f). The DNA sequencing results show the junction regions of Exon 9–Exon 10, Exon 10–Exon 11 and Exon 9–Exon 11 within the cDNA, respectively. NC: negative control; twin-PE: SpCas9(H840A)-RT-based twin-PE; La-twin-PE: La-SpCas9(H840A)-RT-based twin-PE.

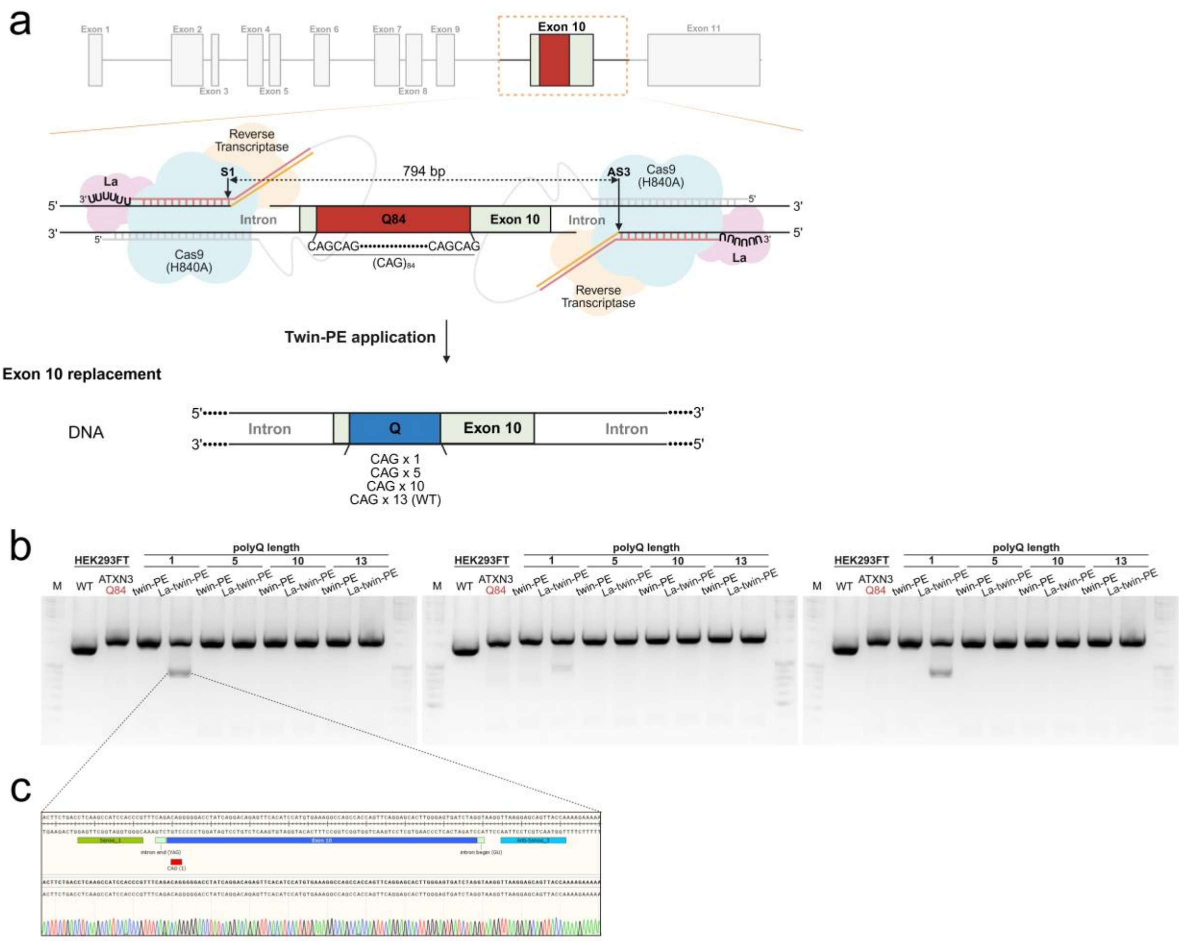

Figure S10. Verification of abnormal polyQ-containing exon 10 replacement using optimized twin prime editing in human-derived mutant HEK293FT-*ATXN3*-Q84 cells. (a) Schematic diagram illustrating the strategy for replacement of abnormal exon 10 with shortened polyQ-containing exon 10 in the *ATXN3* gene of a human-derived SCA3 disease model cell line using twin prime editing. (b) Efficiency analysis of expanded exon 10 replacement with a shortened polyQ-containing exon 10 by twin prime editing targeting *ATXN3* exon 10 using twin-PE or La-twin-PE. Lower gel bands represent replacement with shortened polyQ-containing exon 10. The best pegRNA combinations (S1+AS3) from Figure S8 were used. (c) Sanger sequencing results confirming exon 10 replacement from the shifted bands in (b). NC: negative control; twin-PE: SpCas9(H840A)-RT-based twin-PE; La-twin-PE: La-SpCas9(H840A)-RT-based twin-PE.

SUPPLEMENTAL TABLES

Table S1. Nucleotide sequences of pegRNAs used for twin prime editing at each DNA target in this study. This table is provided as a separate Excel file. (Table_S1.xlsx)

Table S2. Nucleotide sequences of DNA primers used in this study. This table is provided as a separate Excel file. (Table_S2.xlsx)

Table S3. *In silico*–predicted off-target candidate sequences corresponding to each target DNA sequence analyzed in this study. This table is provided as a separate Excel file. (Table_S3.xlsx)
